# Synthesis, Characterization, DFT Mechanistic Study, Antimicrobial Activity, Molecular Modeling, and ADMET Properties of Novel Pyrazole-isoxazoline Hybrids

**DOI:** 10.1021/acsomega.2c05788

**Published:** 2022-12-08

**Authors:** Mohammed Chalkha, Hassan Nour, Khalid Chebbac, Asmae Nakkabi, Lahoucine Bahsis, Mohamed Bakhouch, Mohamed Akhazzane, Mohamed Bourass, Samir Chtita, Yousef A. Bin Jardan, Maria Augustyniak, Mohammed Bourhia, Mourad A.M. Aboul-Soud, Mohamed El Yazidi

**Affiliations:** 1 Engineering Laboratory of Organometallic, Molecular, Materials and Environment, Faculty of Sciences Dhar EL Mahraz, Sidi Mohamed Ben Abdellah University, P.O. Box 1796, 30000 Fez, Morocco; 2 Laboratory of Analytical and Molecular Chemistry, Faculty of Sciences Ben M’Sik, Hassan II University of Casablanca, P.O. Box 7955, Casablanca, Morocco; 3 Laboratory of Biotechnology Conservation and Valorisation of Natural Resources, Faculty of Sciences Dhar El Mahraz, Sidi Mohammed Ben Abdallah University, P.O. Box 1796, Fez 30000, Morocco; 4 Laboratory of Analytical and Molecular Chemistry, Polydisciplinary Faculty, Cadi Ayyad University, P.O. Box 4162, Safi 46000, Morocco; 5 Department of Chemistry, Faculty of Sciences of El Jadida, Chouaïb Doukkali University, P.O. Box 20, El Jadida 24000, Morocco; 6 Laboratory of Bioorganic Chemistry, Department of Chemistry, Faculty of Sciences, Chouaïb Doukkali University, P.O. Box 24, El Jadida 24000, Morocco; 7 Cité de l’innovation, Université Sidi Mohamed Ben Abdellah, Route Immouzer, P.O. Box 2626, 30000 Fez, Morocco; 8 Université de Bordeaux, CNRS, Bordeaux INP, ISM, UMR 5255, 351 Cours de la Libération, F-33405 Talence, Cédex France; 9 Department of Pharmaceutics, College of Pharmacy, King Saud University, 11451 Riyadh, Saudi Arabia; 10 Institute of Biology, Biotechnology and Environmental Protection, Faculty of Natural Sciences, University of Silesia in Katowice, Bankowa 9, 40-007 Katowice, Poland; 11 Higher Institute of Nursing Professions and Technical Health, Laayoune 70000, Morocco; 12 Department of Clinical Laboratory Sciences, College of Applied Medical Sciences, King Saud University, P.O. Box 10219, Riyadh 11433, Saudi Arabia

## Abstract

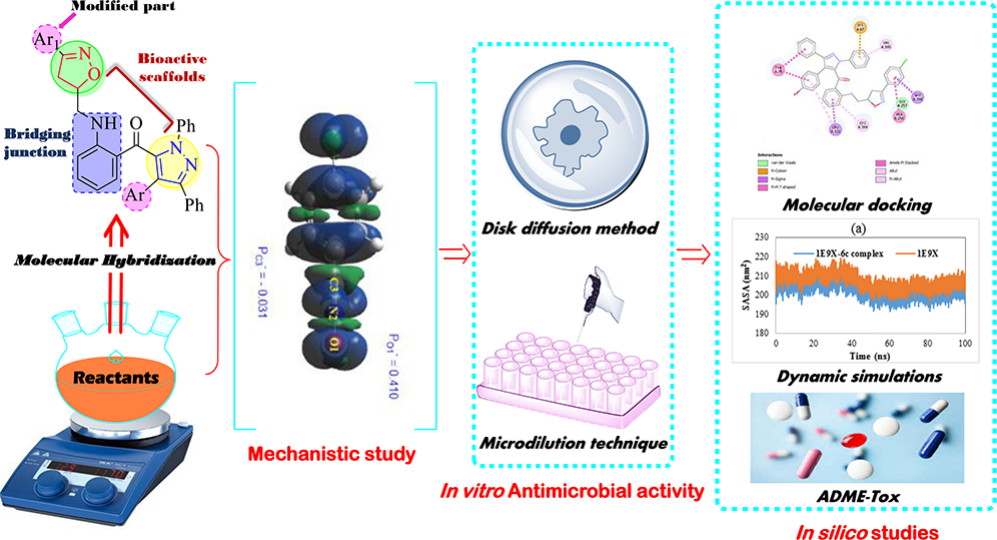

A series of new heterocycle hybrids incorporating pyrazole and isoxazoline rings was successfully synthesized, characterized, and evaluated for their antimicrobial responses. The synthesized compounds were obtained utilizing *N*-alkylation and 1,3-dipolar cycloaddition reactions, as well as their structures were established through spectroscopic methods and confirmed by mass spectrometry. To get more light on the regioselective synthesis of new hybrid compounds, mechanistic studies were performed using DFT calculations with B3LYP/6-31G(d,p) basis set. Additionally, the results of the preliminary screening indicate that some of the examined hybrids showed potent antimicrobial activity, compared to standard drugs. The results confirm that the antimicrobial activity is strongly dependent on the nature of the substituents linked pyrazole and isoxazoline rings. Furthermore, molecular docking studies were conducted to highlight the interaction modes between the investigated hybrid compounds and the *Escherichia coli* and *Candida albicans* receptors. Notably, the results demonstrate that the investigated compounds have strong protein binding affinities. The stability of the formed complexes by the binding between the hybrid compound **6c**, and the target proteins was also confirmed using a 100 ns molecular dynamics simulation. Finally, the prediction of ADMET properties suggests that almost all hybrid compounds possess good pharmacokinetic profiles and no signs of observed toxicity, except for compounds **6e**, **6f**, and **6g**.

## 1 Introduction

Infections caused by parasites or microbes are among the most serious and recurrent diseases in the living world.^1−3^ Unfortunately, despite the progress recorded in the production of anti-infectious drugs, it is limited by the pharmaco-resistance developed by infectious germs.^4,5^ This situation makes the fight against these pathologies an essential issue for public health and food security.^6^ In such a context, it is necessary to constantly develop new drugs for effective and efficient anti-infectious chemotherapy.^7^

To overcome the phenomenon of drug resistance developed by infectious agents, a new approach is conceived in the design of biomolecules endowed with high efficiency and low toxicity.^8−12^ This concept is based on the combination of two or more bioactive molecules in order to produce new hybrid structures, retaining or not the properties of the starting molecules.^10,13−15^ A single-molecule drug that acts on multiple targets is much more attractive in terms of therapeutic efficacy and economic efficiency.^16,17^ Most significantly, molecular hybridization as a novel approach has proven to be very helpful in creating new drugs with increased and specific affinity. These hybrid drugs could be able to act on two or more targets as well as to reduce undesirable side effects. Moreover, this kind of drugs could be considered as a magic solution to overcome the emergence of drug resistance.^9,18−20^ In addition, the synergistic and additive effect of a hybrid drug against the same target provides a beneficial pathway for the treatment of a variety of complex diseases, including cancer, malaria, bacterial infections, etc.^8−11,19−21^

**Figure 1:**
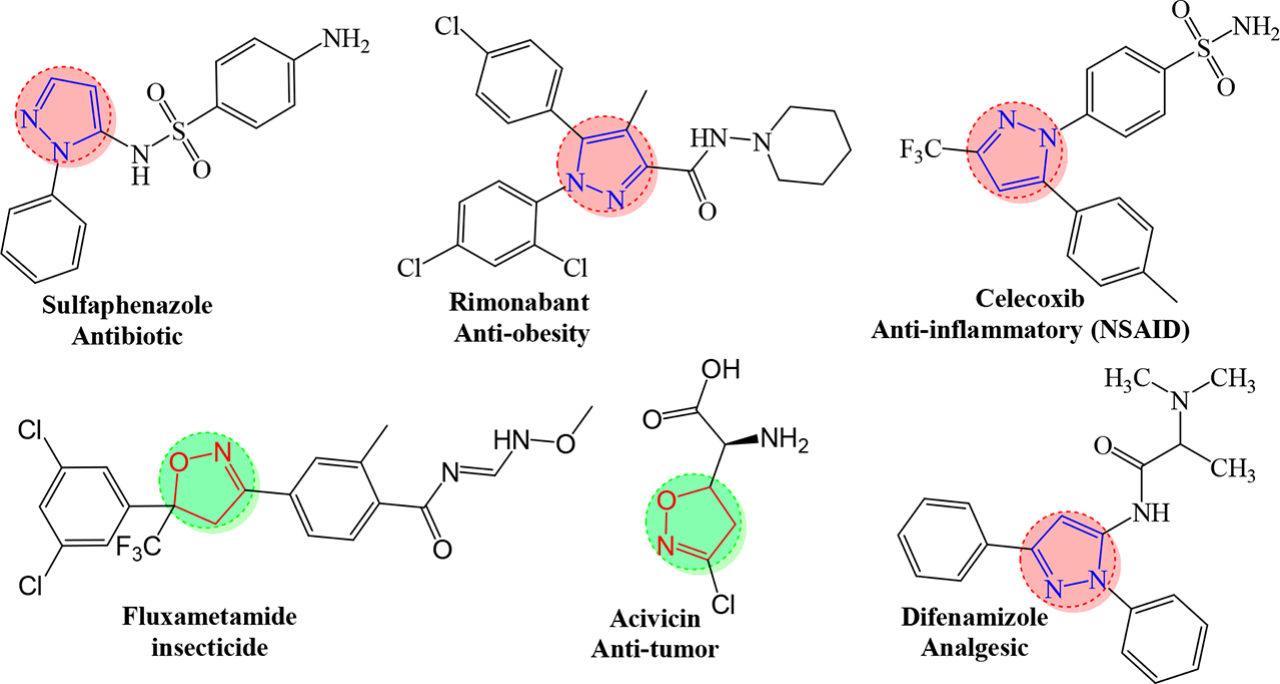
Selected therapeutic agents encompassing an isoxazoline and pyrazole rings.

Heterocycles are among the organic compounds that are widely found in the component of anti-infective treatments.^13,14,22−24^ They have become increasingly involved in the discovery of new classes of medicines with new mechanisms of action.^25,26^ Heterocycles containing pyrazole or isoxazoline pharmacophores occupy an important place among the classes of compounds known for their therapeutic and pharmacological activities.^27−29^ These pharmacophores are in fact the basic structure of many natural and synthetic molecules with anticancer, anti-inflammatory, antimicrobial, analgesic, and corrosion inhibitor activities (Figure 1).^27,28,30^ They are also ubiquitous in several pesticides such as herbicides, fungicides, and insecticides.^31,32^ Indeed, these two entities constitute excellent pharmacophores for the development of many drug candidates.^30,32^ The combination of these two pharmacophores in a single hybrid structure can result in new molecules with high chemical stability and good biological profile.

In continuation of our previous efforts in the development of new aza-heterocyclic antimicrobial candidates,^33−36^ we report in the present work the synthesis, structural identification, and biological activity assessment of new poly heterocyclic molecules incorporating both pyrazole and isoxazoline cores. The synthesis of these heterocycles is carried out from aza-aurones by N-alkylation and 1,3-dipolar cycloaddition reactions. The structural identification of the studied compounds is established by spectroscopic techniques, and high-resolution mass spectrometry. The DFT study is performed to explain the experimental results and to understand precisely the regioselectivity outcome of the reaction between nitrile oxides and allylated pyrazole **4** used as a dipolarophile. In addition, the antimicrobial activity of the synthesized compounds is evaluated against four microbial species and compared to that of the standard drugs. The molecular docking study was carried out on the studied compounds toward *Escherichia coli* and *Candida albicans* receptors, to support the *in vitro* results and to determine the likely interactions that occur between the hybrid ligands and the targeted proteins. Finally, molecular dynamics simulations are executed to confirm the stability of the ligand–protein complexes resulting from molecular docking.

## 2 Results and Discussion

### 2.1 Synthesis and Spectral Analysis

**Scheme 1:**
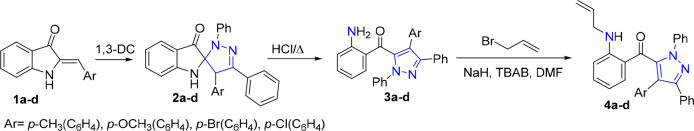
Synthetic Methods of Dipolarophiles **4a**–**d**

**Scheme 2:**
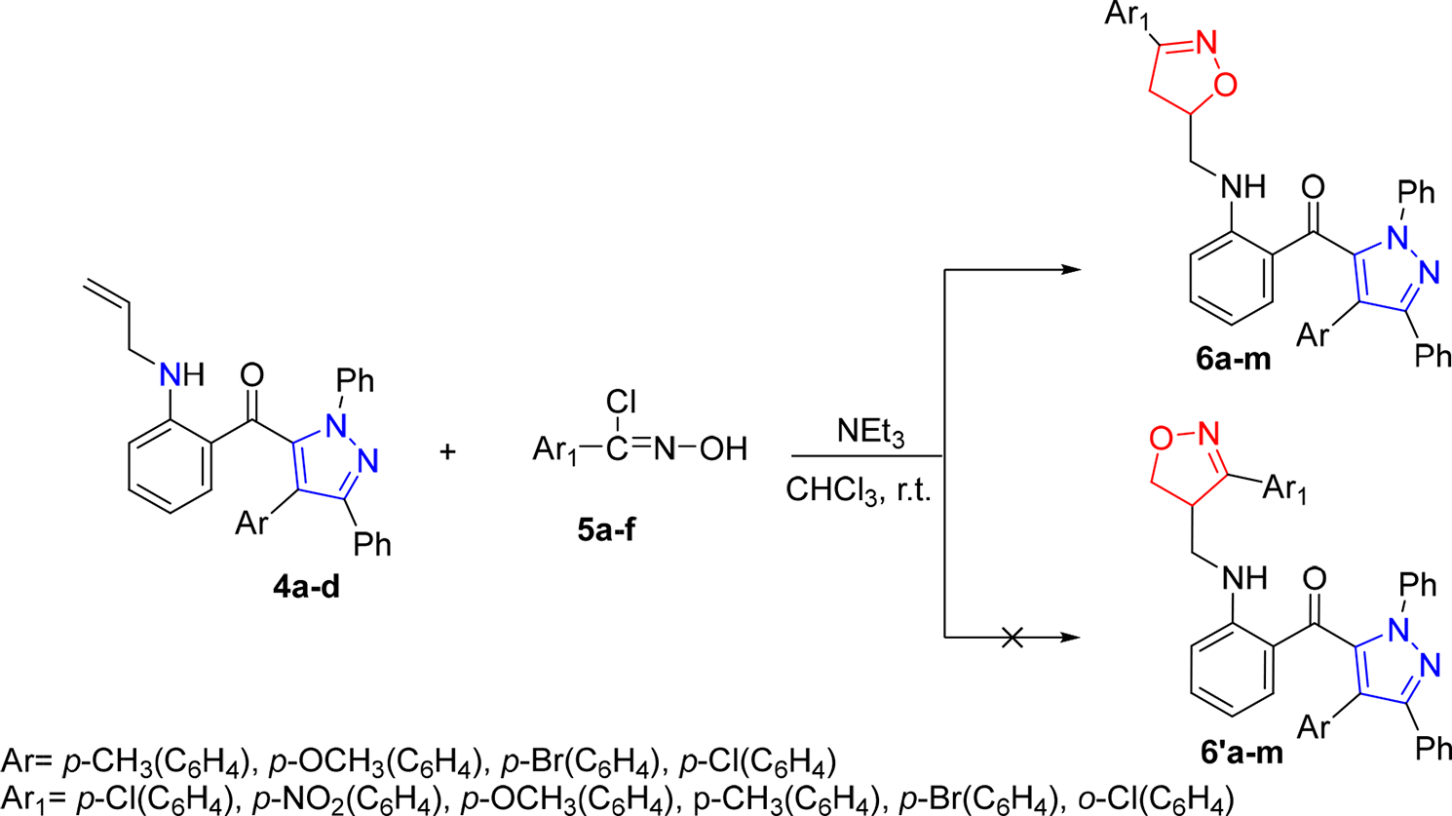
Synthetic Pathway of Hybrid Molecules **6a**–**m**

The synthetic pathways employed to prepare the new target molecules are outlined in Schemes 1 and 2. In a multistep process, the intermediates 5-(2-aminobenzoyl)-4-aryl-1,3-diphenylpyrazoles **3a**–**d** are synthesized by the 1,3-dipolar cycloaddition reaction between aza-aurones and nitrilimines, followed by a ring opening of spirocycloadduct as described in our previous work.^35^ Then, the obtained intermediates incorporating a free amino group are subjected to the action of allyl bromide in DMF in the presence of sodium hydride (NaH) during the necessary time at room temperature (Scheme 1).^33^ The treatment and purification of the reaction mixture allowed us to isolate the corresponding N-allylated pyrazole compounds **4a**–**d**, in good yields. The identification of the structure of the obtained **4a**–**d** products is established by usual spectroscopic methods, such as infrared (IR), proton, and carbon 13 NMR, and mass spectrometry.

In the IR spectra of the products **4a**–**d**, we observe an absorption band around 3350 cm^–1^ characteristics of the vibration of the NH bond of the secondary amine; which highlights the substitution of the primary amine. The ^1^H NMR spectra of the allylated pyrazoles **4a**–**d** display a multiplet signal between 5.18 and 5.25 ppm belongs to the two protons of the allyl group (=C**H**_**2**_), a multiplet signal between 5.86 and 5.99 ppm attributable to the internal vinylic proton (H_2_C=C**H**−) and the presence of an another multiplet signal between 3.89 and 3.94 ppm corresponding to the two protons of the methylene group linked to the nitrogen atom (−C**H**_**2**_–N). The proton of the N**H** group resonates around 8.96 ppm as a triplet signal. In addition, on their ^13^C NMR spectra, we note in particular the existence of a signal at 44 ppm corresponds to the carbon of the methylene group (**C**H_2_–N). A signal at 116 ppm relative to the terminal vinylic carbon (=**C**H_2_), a signal at 133 ppm attributable to the internal vinylic carbon =**C**H–, the carbon of the carbonyl group resonates at 190 ppm. The structure and purity of the synthesized products were confirmed by high resolution mass spectrometry (LC-MS-MS). The HRMS data of all the synthesized products are in good agreement with the proposed structures and with the calculated values for the molecular ions [M–H]^+^.

The obtained N-allylated pyrazoles **4a**–**d** are then used as dipolarophiles for the synthesis of new hybrid aza-heterocyclic compounds **6a**–**m** via the 1,3-dipolar cycloaddition with nitrile oxides. The nitrile oxides are generated *in situ* from chlorinated aldoximes^37,38^**5a**–**f** by the action of triethylamine in chloroform (CHCl_3_). The reaction results in the formation of the new hybrid cycloadducts **6a**–**m**. The skeleton of the new cycloadducts is formed by isoxazolinic and pyrazolic nuclei linked to each other by a methylamino benzoyl moiety (Scheme 2). The structural elucidation of the newly synthesized hybrid heterocycles is realized by FTIR, ^1^H NMR, ^13^C NMR, Homonuclear (^1^H–^1^H), and Heteronuclear (^1^H–^13^C) 2D-NMR spectra. The obtained products are also confirmed by high-resolution mass spectrometry (HRMS). The complete spectral data and physical properties of the synthesized compounds are described in the Supporting Information.

**Figure 2:**
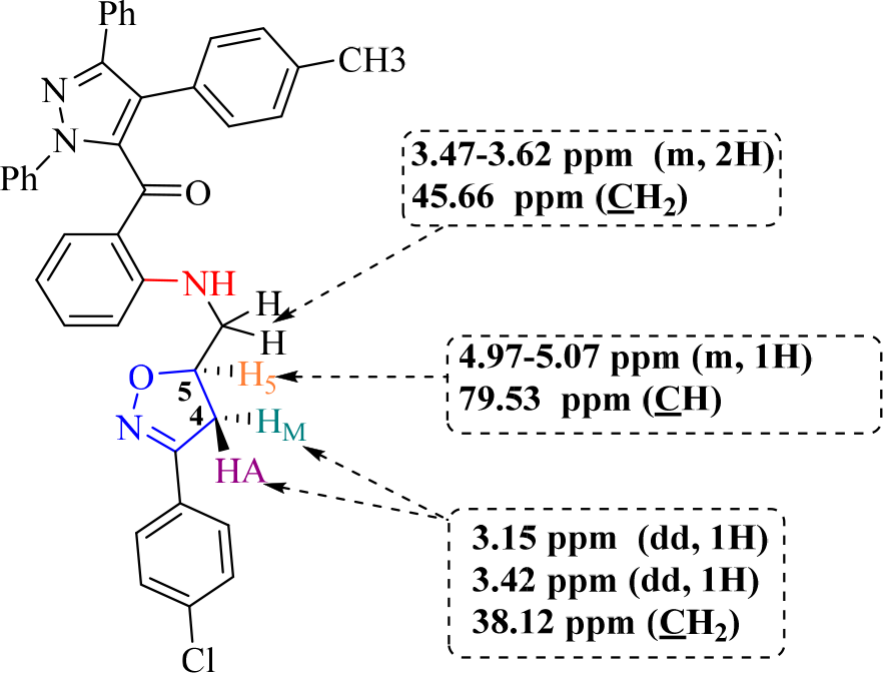
Characteristic signals in the ^1^H and ^13^C NMR spectra of compound **6a**.

The 1,3-dipolar cycloaddition reactions between allylated pyrazoles **4a**–**d** and nitrile oxides **5a**–**e**, resulted in a single regioisomer **6** in the absence of any trace of the regioisomer **6′** (Scheme 2). This result is in perfect agreement with literature data on the regiochemistry of the 1,3-dipolar cycloaddition of nitrile oxides with allyl compounds controlled by both steric and electronic factors.^39−42^ The regiochemistry of the obtained isoxazolines **6a**–**m** is proposed on the basis of the chemical shifts of the protons (H_4_ and H_5_) and carbons (C_4_ and C_5_) of the isoxazolinic ring with reference to the analogous compounds (Figure 2).^39−42^

The NMR data of hybrid compound **6a** are discussed as a representative compound of this study. Indeed, the ^1^H NMR spectrum of hybrid compound **6a** show the existence of a multiplet between 4.98 and 5.07 ppm corresponding to the proton of the methine group (C**H**_5_) of the stereogenic center of the isoxazoline ring. This resulting chemical shift value agrees with the chemical shift of the proton H_5_ of the 3,5-disubstituted isoxazoline regioisomer.^39−42^ The two diastereotopic hydrogen atoms linked to the methylene groups adjacent to the stereogenic center of the isoxazoline ring C_4_**H**_A_**H**_M_ resonate as two doublet-of-doublets at 3.15 ppm (*J* = 7.3, 16.69 Hz) and 3.43 ppm (*J* = 10.47, 16.69 Hz), and forms an AB system due to geminal and vicinal proton–proton coupling (Figure 2). The chemical shift values of protons H_5_ and H_4_ in the isoxazoline ring confirm the formation of 3,5-disubstituted isoxazoline as a single regioisomer.^39−42^ It also shows the presence of a multiplet signal between 3.50 and 3.57 ppm attributable to the two methylene protons linked to the nitrogen atom (C**H**_2_–N), and a triplet signal located at 8.99 ppm corresponds to the proton of −NH group.

Furthermore, on the ^13^C NMR spectrum of the same compound recorded in CDCl_3_, we note the presence of a signal located at 38 ppm attributable to the methylene carbon (**C**_4_H_A_H_M_) of isoxazoline ring, and the asymmetric carbon of the methine group (**C**_5_H) resonates at 79 ppm (Figure 2). Moreover, a signal at 45 ppm relative to the carbon of the methylene group linked to the nitrogen atom (N–**C**H_2_), another signal located at 190 ppm corresponding to the carbon of the carbonyl group.

**Figure 3:**
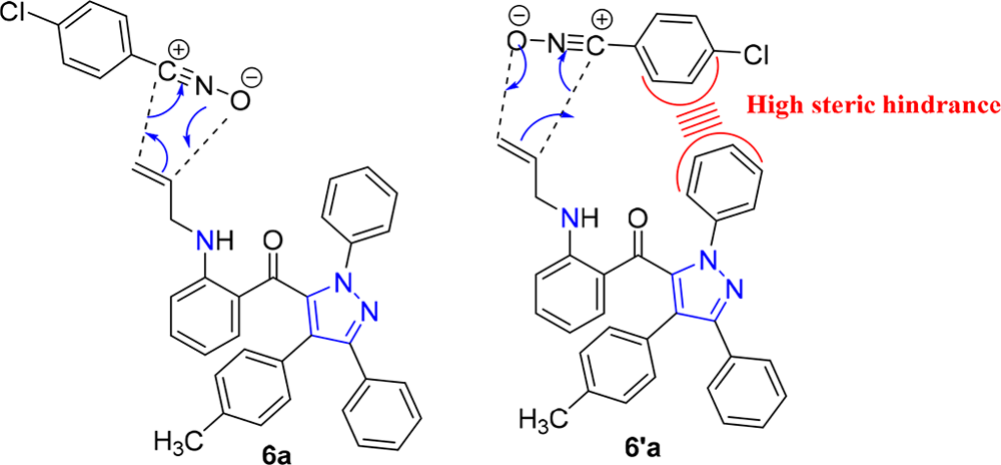
Steric control of the regioselectivity of the 1,3-dipolar cycloaddition reaction of dipolarophile **4a** and nitrile oxide **5a**.

The chemical shift obtained for the asymmetric carbon C_5_ of isoxazoline ring (79 ppm) agrees with the proposed regiochemistry, and the literature data.^39−41^ This largely unshielded chemical shift value for an aliphatic carbon can be due to the presence of an electronegative atom like nitrogen. This suggests that the obtained regioisomer during this reaction is a 3,5-disubstituted regioisomer **6** rather than 3,4-disubstituted regioisomer **6′**. For the regioisomer **6′**, the signal of the stereogenic carbon would be expected to have a lower chemical shift value since it would be distant from the oxygen atom (Scheme 2). The spectral data are in favor of regioisomer **6** and confirm the regiospecificity of the reaction of nitrile oxides on allylated pyrazoles used as dipolarophiles. This regiocontrol process can be explained by the steric control of the nitrile oxide **5a** approach on both sides of the dipolarophile **4a** as described in Figure 3. The suggested regiochemistry is subjected to a theoretical study to explain and confirm the observed results.

The attribution of different signals of the 1D NMR spectra (^1^H and ^13^C) was further confirmed using COSY and HSQC 2D NMR. On the homonuclear (^1^H–^1^H) 2D NMR spectrum of compound **6a** (Figure S14), a good correlation between the methylene and methine protons of the isoxazoline nucleus is observed, as well as the correlation between the methine proton and the methylene protons linked to the nitrogen atom. Moreover, on the heteronuclear (^1^H–^13^C) 2D NMR spectrum of compound **6a** (Figure S15), we notice that the two methylene protons of the isoxazoline resonate at 3.15 and 3.43 ppm are correlated with the same signal at 38 ppm attributed to carbon (**C**_4_). This further confirms that the two protons of the methylene group are chemically nonequivalent.

Additionally, the FT-IR spectrum of hybrid compound **6a** indicated the existence of two absorption bands which are characteristic of the vibrations of the C–O and C=N bonds in the isoxazoline core and appear at 1222 and 1573 cm^–1^, respectively. Likewise, it demonstrates the presence of two additional absorption bands at 1622 and 3290 cm^–1^, which were assigned to the vibrations of the C=O and NH bonds of the carbonyl group and the secondary amine, respectively.

Otherwise, the mass spectra of the new pyrazole-isoxazoline hybrids indicated the presence of a molecular ion peak [M + H]^+^, which corresponds to a single molecule’s precise mass and is coherent with the chemical formula of the proposed structures (Table S1). For instance, the mass spectrum of hybrid **6a** exhibits a peak for the protonated molecular ion [M + H]^+^ at *m*/*z*: 623.22079, confirming the proposed structure’s molecular formula [C_39_H_31_ClN_4_O_2_] (Figure S16).

### 2.2 Mechanistic Study

Recently, the 1,3-dipolar cycloaddition reactions are reported as an excellent process for the syntheses of new heterocyclic compounds.^43−45^ In our case, the 1,3-dipolar cycloaddition reaction of the allylated pyrazole **4a** and the nitrile oxide **5a**, taken as a representative example for this study, conducts to the pyrazole-isoxazoline hybrid heterocycles. Depending on the regioselective attacks, two possible pathways were investigated, as shown in Figure 3.^46,47^ Initially, the optimized structures of the allylated pyrazole **4a** and the nitrile oxide **5a** were performed using B3LYP/6-31G(d,p) basis set, see Figure S1 in Supporting Information, and the DFT investigations have been summarized into two sections. The first one is the analysis of the global and local reactivity indexes are computed through the analysis of the Conceptual Density Functional Theory (CDFT) indices; and the last one is the investigation of the possible cycloaddition reaction profiles based on energy barriers and all reported findings.

Recently, the global reactivity indexes obtained through the conceptual DFT calculations were reported as a powerful tool used to explain the obtained regioselectivity in 1,3-dipolar reactions.^48,49^ The global reactivity indexes for the nitrile oxide **5a** and the allylated pyrazole **4a** were calculated and summarized in Table 1. The electronic chemical potential of **4a** is only slightly higher than that of **5a** by 0.25 eV indicating that the compound **4a** will not be very likely to transfer electron density toward the compound **5a**, and the corresponding 1,3-dipolar reaction is characterized by a low polar character.^50,51^ The analysis of the global electrophilicity and nucleophilicity indexes, within the electrophilicity^52^ and nucleophilicity scales,^53^ shows that **4a** acts as a strong electrophile (ω = 1.81 eV) and a strong nucleophile (*N* = 3.49 eV), while **5a** acts as a moderate electrophile (ω = 1.56 eV) and a moderate nucleophile (*N* = 2.65 eV). Analysis of the global CDFT indices confirms that **5a** has a lower electrophilic and nucleophilic character which does not allow its participation in polar processes; consequently, the corresponding 1,3-dipolar reaction between **4a** and **5a** compounds will have a very low polar character. Moreover, these results indicate that **4a** acts as an electrophile and **5a** as a nucleophile.

**Table 1:** Global Electronic Proprieties and Reactivity Indexes at **4a** and **5a** Compounds (in eV)

	HOMO	LUMO	μ	η	ω	*N*
allylated pyrazole **4a**	–5.62	–1.80	–3.71	3.82	1.81	3.49
nitrile oxide **5a**	–6.46	–1.45	–3.96	5.01	1.56	2.65

**Figure 4:**
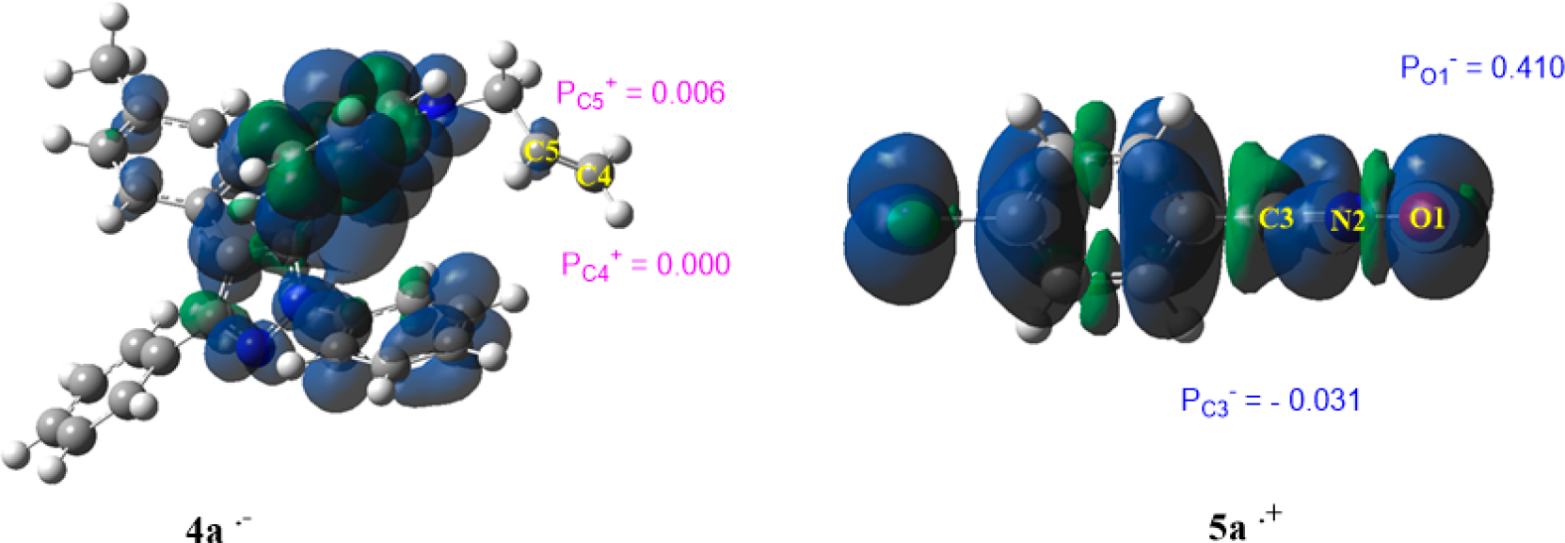
Mulliken atomic spin densities representations of radical ions together with the corresponding Parr functions for **4a** and **5a**; values are in eV.

Recently, the analysis of local reactivity indexes was reported as an excellent tool to explain the regio- or chemoselectivity issues in 1,3-dipolar cycloaddition reactions.^54^ The values of the local electrophilicity at **4a** and the local nucleophilicity at **5a** are calculated and reported in Figure 4. The analysis of the local electrophilicity (P_k_^+^) at **4a** shows that the most electrophilic center is the carbon atom (C5), (P_C5_^+^) = 0.006 eV, and the more nucleophilic activated center at **5a** is the oxygen atom O1, (P_O1_^–^) = 0.41 eV. These results confirm the regioselective synthesis of compound **6a** and can allow us to understand the observed regioselectivity.

**Figure 5:**
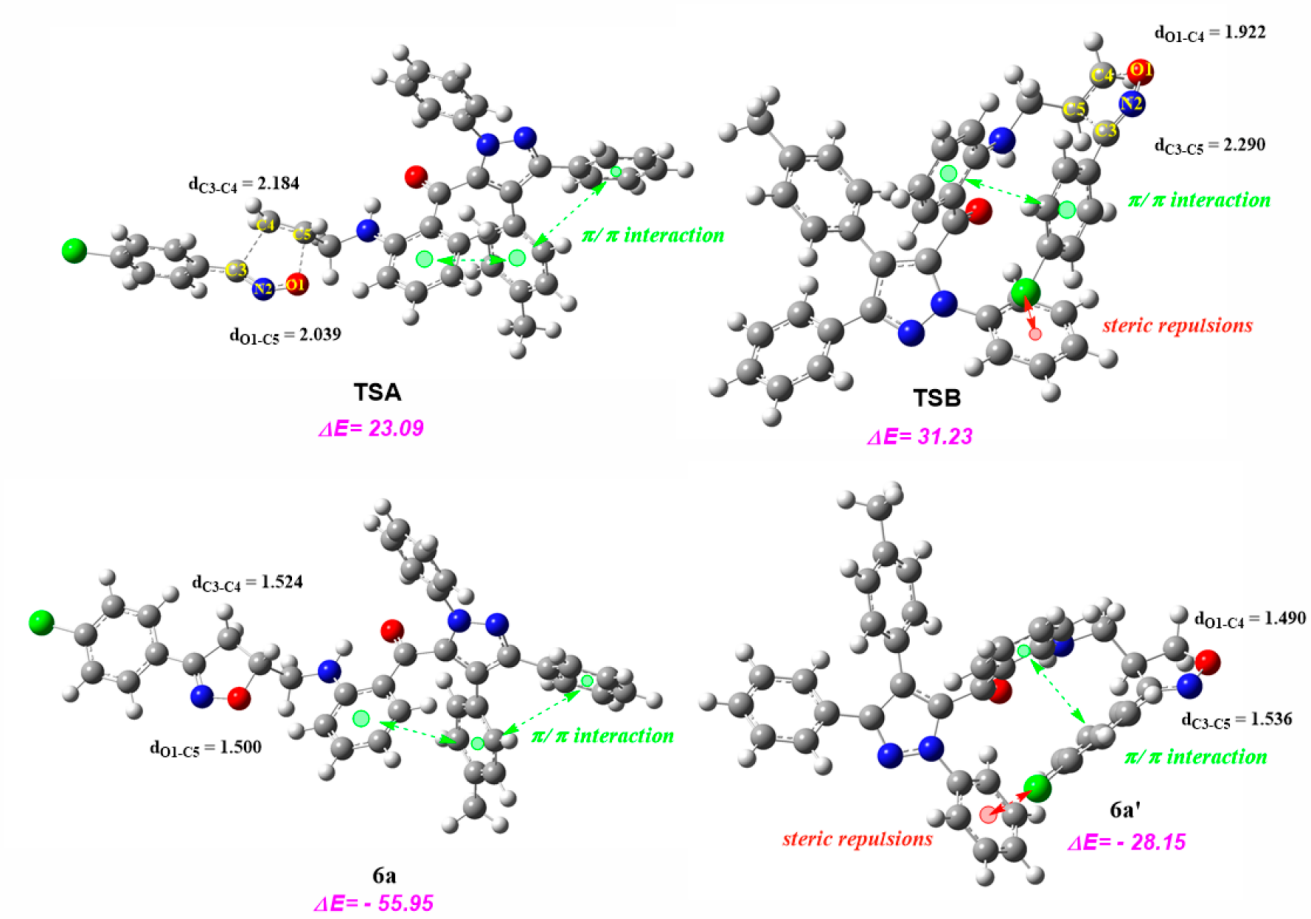
Energy profiles for the corresponding cycloaddition reaction between the nitrile oxide (**5a**) and the allylated pyrazole (**4a**). Values of energies are in kcal/mol and lengths are in Å.

The mechanistic study for the obtained compound **6a** was performed through the investigation of both considered reaction pathways for the cycloaddition between the nitrile oxide (**5a**) and the allylated pyrazole (**4a**). The analysis of the reaction profile confirms that this cycloaddition reaction takes place via a one-step mechanism. The relative energies are computed, using chloroform as a solvent, and summarized in Figure 5. Path B has an activation energy of 31.23 kcal/mol, which is endothermic by 8.14 kcal/mol, compared with path A. The structural analysis of these TSs indicates the presence of unfavorable steric interactions between the chlorine atom in the **5a** framework and π electron of the phenyl group in the **4a** framework in **TSB**, see Figure 5, whereas in **TSA** the steric interactions are not observed. The reaction products have exothermic by 55.95 and 28.15 kcal/mol for **6a** and **6a′**, respectively. These results can be explained by the presence of only a stabilizing π/π interaction in **6a** compared to the **6a′** product.^55^ Selected geometrical parameters of the TSs and products for the two reaction possible pathways are shown in Figure 5. In TSA, the length of the O1–C5 and C3–C4 bonds are 2.039 and 2.184 Å, respectively. However, in compound **6a**, the C3–C4 bond length (1.524 Å) is longer than the O1–C5 bond length (1.500 Å). Similarly, in **TSB**, the C3–C5 bond length (2.290 Å) is longer than the O1–C4 bond length (1.922 Å) and in **6a′** product, these lengths are 1.536 and 1.490 Å for C3–C5 and O1–C4 bond lengths, respectively. All these findings indicate that the more favorable product of this cycloaddition reaction is compound **6a** which is in good agreement with the experimental observations.

### 2.3 Antimicrobial Activity and Structure Activity Relationship (SAR) Study

The newly synthesized class of heterocyclic compounds pyrazole-isoxazoline hybrids **6a**–**m** was evaluated for their antibacterial activity against two Gram-positive bacteria: *Staphylococcus aureus* (CECT 976) and *Bacillus subtilis* (DSM 6633) and one Gram-negative bacteria: *Escherichia coli* (K12), we also investigated their antifungal effectiveness against the fungus *Candida albicans* (ATCC 10231). This is done with the purpose to assess the sensitivity of the microbes to various synthesized hybrid compounds, as well as to examine the impact of coupled substituents and the incorporation of the pharmacophore isoxazoline on the biological activity of the hybrid compounds, while comparing the obtained results to our previous works. *In vitro* antimicrobial screening of the target compounds was performed using the disk diffusion method and the broth microdilution technique to determine the minimum inhibitory concentration (MIC). The obtained outcomes are compared with those of the antibiotics employed as positive controls against the bacterial species, namely Ampicillin and Streptomycin; Fluconazole was chosen as the standard drug for antifungal activity (see Tables 2 and S2).

**Table 2:** Antimicrobial Activity of Hybrid Heterocycles **6a**–**m** and Standard Drugs against Microbes Tested by the Disk Diffusion Method

zone inhibition in mm (1 mg/mL) (means ± SD)^a^										
			tested microorganisms							
compounds			*E. coli* (*−ve*)		*B. subtilis* (*+ve*)		*C. albicans*		*S. aureus* (*+ve*)	
no.	Ar	Ar_1_	ZOI (mm)	AI (%)	ZOI (mm)	AI (%)	ZOI (mm)	AI (%)	ZOI (mm)	AI (%)
**6a**	*p*-CH_3_(C_6_H_4_)	*p*-Cl(C_6_H_4_)	12.5 ± 0.5	52	8 ± 00	50	10.5 ± 01	50	10.5 ± 0.5	45.6
**6b**	*p*-OCH_3_(C_6_H_4_)	*p*-Cl(C_6_H_4_)	14 ± 01	58	NE	-	10.25 ± 0.75	48.8	10 ± 01	43.4
**6c**	*p*-Br(C_6_H_4_)	*p*-Cl(C_6_H_4_)	12.5 ± 0.5	52	14.5 ± 1.5	90.6	12.25 ± 1.87	58.3	12 ± 00	52.1
**6d**	*p*-Cl(C_6_H_4_)	*p*-Cl(C_6_H_4_)	13.5 ± 1.5	56	10 ± 00	62.5	11.5 ± 0.5	54.7	18 ± 03	78.6
**6e**	*p*-Br(C_6_H_4_)	*p*-NO_2_(C_6_H_4_)	9.5 ± 0.5	39	NE	-	12 ± 01	57.1	10 ± 01	43.4
**6f**	*p*-CH_3_(C_6_H_4_)	*p*-NO_2_(C_6_H_4_)	12 ± 01	50	8.5 ± 1.5	53.1	11.75 ± 1.25	55.9	10.5 ± 0.5	45.6
**6g**	*p*-OCH_3_(C_6_H_4_)	*p*-NO_2_(C_6_H_4_)	15 ± 01	62.5	NE	-	11.25 ± 0.75	53.5	12 ± 02	52.1
**6h**	*p*-Br(C_6_H_4_)	*p*-OCH_3_(C_6_H_4_)	13 ± 01	54.1	15 ± 01	93.7	12.25 ± 2.12	58.3	12 ± 01	52.1
**6i**	*p*-OCH_3_(C_6_H_4_)	*p*-OCH_3_(C_6_H_4_)	10.5 ± 1.5	43.7	9 ± 01	56.2	10.5 ± 0.5	50	11.5 ± 0.5	50
**6j**	*p*-CH_3_(C_6_H_4_)	*p*-CH_3_(C_6_H_4_)	13 ± 02	54.1	8.5 ± 0.5	53.1	10.5 ± 0.5	50	13.5 ± 3.5	58.6
**6k**	*p*-OCH_3_(C_6_H_4_)	*p*-Br(C_6_H_4_)	13.5 ± 1.5	56	12 ± 00	75	11 ± 01	52.3	11 ± 02	47.8
**6l**	*p*-OCH_3_(C_6_H_4_)	*p*-CH_3_(C_6_H_4_)	13 ± 03	54.1	9.5 ± 0.5	59.3	11.25 ± 1.25	53.5	7.5 ± 0.5	32.6
**6m**	*p*-Cl(C_6_H_4_)	*o*-Cl(C_6_H_4_)	14 ± 00	58	7 ± 0.5	43.7	11.5 ± 0.3	54.7	11 ± 02	47.8
ampicillin			NT	-	16 ± 0.3	100	NT	-	23 ± 0.5	100
fluconazole			NT	-	NT	-	21 ± 01	100	NT	-
streptomycin			24 ± 1.5	100	NT	-	NT	-	NT	-
DMF			***6 (NZ)***	***-***	***6 (NZ)***	***-***	***6 (NZ)***	***-***	***6 (NZ)***	***-***
filter paper disk			***6 (NZ)***	***-***	***6 (NZ)***	***-***	***6 (NZ)***	***-***	***6 (NZ)***	***-***

a Values are represented as mean ± standard deviations of twice experiments for bacteria and four experiments for the yeast. NZ = No zone of inhibition; diameter of filter disk is 6 mm; NT: Not tested; NE: This compound has No Effect or Negligible Effect on this strain; AI: Activity Index.

According to the obtained results from the preliminary antimicrobial screening presented in Table 2, the target hybrid compounds **6a**–**m** show variable antimicrobial activity toward the tested pathogenic microorganisms although their structures are similar. The observed difference in inhibitory activity between the various **6a**–**m** hybrid compounds investigated may be related to the effect and nature of the substituents of the phenyl rings (Ar and Ar_1_) of the pyrazole and isoxazoline cores. This is consistent with reported works in the literature that demonstrate how electron-donating and electron-withdrawing substituents like nitro, chlorine, bromine, methyl, and methoxy groups affect biological activity.^56−58^ As the obtained results indicate, some compounds with specific substituents turned out to be more active. Among the examined hybrids, compound **6d** (Ar = *p*-Cl(C_6_H_4_), Ar_1_ = *p*-Cl(C_6_H_4_) displays the best antibacterial activity against *S. aureus* with an inhibition zone of (18 ± 03) mm and a percentage of inhibition of 78.6%, as compared to the reference antibiotic, ampicillin (23 ± 0.5) mm. This powerful inhibitory effect obtained for compound **6d** is most likely owing to the existence of two chlorine atoms (Cl) in the para position of the aromatic rings (Ar and Ar_1_). However, the change in the position of the chlorine atom from para to ortho in the Ar_1_ moiety resulted in a significant decrease of the antibacterial effect of compound **6m** (Ar = *p*-Cl(C_6_H_4_), Ar_1_ = *o*-Cl(C_6_H_4_)) against the same strain (*S. aureus*), which is consistent with other previously described studies on homologous compounds.^59^ The decrease in bioactivity showed for compound **6m** when compared to that of hybrid compound **6d** may be related to unfavorable interactions of chlorine in the ortho-phenyl position, which prevents its ability to inhibit the *S. aureus* strain. The antimicrobial efficacy of the others target hybrids is also decreased when chlorine atom is replaced by other groups in the para position of the aromatic rings (Ar and Ar_1_).

The Gram-positive bacteria *B. subtilis* (DSM 6633) showed remarkable sensitivity to the hybrid compounds **6h** (Ar= *p*-Br(C_6_H_4_), Ar_1_= *p*-OCH_3_(C_6_H_4_) and **6c** (Ar= *p*-Br(C_6_H_4_), Ar_1_= *p*-Cl(C_6_H_4_) with an inhibition zone of (15 ± 01) mm and (14.5 ± 1.5) mm and inhibition rates of 93.7% and 90.6%, respectively, compared with the reference drug, Ampicillin (16 ± 0.3) mm. The slight decrease observed for compound **6c** can be attributed to the chlorine atom as an electron withdrawing group, which affects negatively the activity. This was confirmed when introducing the strongly electron-withdrawing group NO_2_ into compound **6e** (Ar = *p*-Br(C_6_H_4_), Ar_1_ = *p*-NO_2_(C_6_H_4_). The substitution of bromine with the OCH_3_ group in compound **6k** (Ar = *p*-OCH_3_(C_6_H_4_), Ar_1_ = *p*-Br(C_6_H_4_) showed a slight drop in activity compared to compound **6h** (Ar = *p*-Br(C_6_H_4_), Ar_1_ = *p*-OCH_3_(C_6_H_4_), suggesting that the presence of bromine atom in the Ar ring is best for inhibiting the growth of the bacteria *B. subtilis*. This result is similar to those described in the literature for compounds containing the same substituents.^60^

On the other hand, the Gram-negative bacteria *E. coli* is inhibited by almost the majority of the tested hybrid compounds with moderate to good antibacterial effect in comparison with the control. Among the evaluated hybrid compounds, compound **6g** (Ar = *p*-OCH_3_(C_6_H_4_), Ar_1_ = *p*-NO_2_(C_6_H_4_)) is the most active against *E. coli* with an inhibition zone of (15 ± 01) mm and an inhibition rate of 62.5% compared to streptomycin (24 ± 1.5 mm). The substitution of the methoxy group (OCH_3_) by a bromine atom or methyl group in compounds **6e** (Ar = *p*-Br(C_6_H_4_), Ar_1_ = *p*-NO_2_(C_6_H_4_)) and **6f** (Ar = *p*-CH_3_(C_6_H_4_), Ar_1_ = *p*-NO_2_(C_6_H_4_)) leads to a decrease of the antibacterial efficacy. Similarly, the replacement of the substituent NO_2_ with chlorine or bromine in the hybrid compounds **6b** (Ar = *p*-OCH_3_(C_6_H_4_), Ar_1_ = *p*-Cl(C_6_H_4_)) and **6k** (Ar = *p*-OCH_3_(C_6_H_4_), Ar_1_ = *p*-Br(C_6_H_4_)) also resulted in a slight decrease in the antibacterial effect. These findings suggest that the presence of both groups OCH_3_ in Ar and NO_2_ in Ar_1_ is most effective at inhibiting the growth of the Gram-negative bacteria *E. coli*, which is consistent with those reported in previous studies.^60,61^ Overall, the tested hybrid heterocycles **6a**–**m** had a greater inhibitory activity index against Gram-positive bacteria than Gram-negative bacteria in comparison with standard drugs, which corroborates the data in the literature.^60,62^

For the antifungal activity, the fungal strain *C. albicans* exhibited moderate sensitivity toward almost all of the tested hybrid heterocycles. Among them, compounds **6c** (Ar = *p*-Br(C_6_H_4_), Ar_1_ = *p*-Cl(C_6_H_4_)), **6h** (Ar = *p*-Br(C_6_H_4_), Ar_1_ = *p*-OCH_3_(C_6_H_4_)), and **6e** (Ar = *p*-Br(C_6_H_4_), Ar_1_ = *p*-NO_2_(C_6_H_4_)) showed a moderate antifungal capacity with inhibition zones of (12.25 ± 1.87) mm (58.3%), (12.25 ± 2.12) mm (58.3%), and (12 ± 01) mm (57.1%), as compared to Fluconazole (21 ± 01) mm. According to these findings, it can be remarked that the electron-withdrawing substituent NO_2_ induced a slight decrease in the antifungal effect. The same phenomenon is observed when substituting bromine by chlorine in the Ar ring of hybrid compounds **6d** (Ar = *p*-Cl(C_6_H_4_), Ar_1_ = *p*-Cl(C_6_H_4_)) and **6m** (Ar = *p*-Cl(C_6_H_4_), Ar_1_ = *o*-Cl(C_6_H_4_)). This latter shows a growth inhibition of 54.7% (11.5 mm) against the fungus *C. albicans*. According to the obtained results, the bromine atom in the Ar ring is crucial for the antifungal activity. This is in contrast to our previous study, which demonstrated that replacing a bromine atom with a more electronegative halogen, like chlorine, improves the antifungal activity of pyrazole derivatives against the strain *C. albicans*.^34^

The MIC results for the investigated hybrid heterocyclic compounds are collected in Table S2. The results indicate that among the tested hybrids, compounds **6d** (Ar = *p*-Cl(C_6_H_4_), Ar_1_ = *p*-Cl(C_6_H_4_)), **6m** (Ar = *p*-Cl(C_6_H_4_), Ar_1_ = *o*-Cl(C_6_H_4_)), and **6g** (Ar = *p*-OCH_3_(C_6_H_4_), Ar_1_ = *p*-NO_2_(C_6_H_4_)) proved to be the most active against *S. aureus* with MIC values of (18.23 ± 0.55) μM, (36.5 ± 0.88) μM, and (48.1 ± 0.53) μM close to the ampicillin standard (11.1 ± 0.43) μM. Compound **6c** (Ar = *p*-Br(C_6_H_4_), Ar_1_ = *p*-Cl(C_6_H_4_) shows excellent activity against *B. subtilis* and *C. albicans* with MICs of (34.14 ± 0.11) μM and (91.1 ± 0.33) μM, respectively. Furthermore, compounds **6g** (Ar = *p*-OCH_3_(C_6_H_4_), Ar_1_ = *p*-NO_2_(C_6_H_4_)) and **6i** (Ar = *p*-OCH_3_(C_6_H_4_), Ar_1_ = *p*-OCH_3_(C_6_H_4_)) exhibit potent antibacterial activity against Gram-negative *E. coli* with MIC values of (48.1 ± 0.63) μM and (49.2 ± 0.23) μM, respectively, compared to streptomycin (13.4 ± 0.52) μM. Overall, the bioactivity results observed for the hybrid compounds pyrazole-isoxazoline **6a**–**m** are promising and clearly greater than those obtained for pyrazole derivatives only described in our previous work.^33,34^ These results can be attributed to the synergistic effect between the pyrazole and isoxazoline pharmacophores, which is consistent with the concept of molecular hybridization.^9,16^

### 2.4 Molecular Docking Studies

The molecular docking analysis was performed to evaluate the affinity of the synthesized compounds toward the targeted proteins related to antimicrobial activity. The docking results are presented in Table S3. All investigated compounds exhibited low and negative binding energy, demonstrating their high affinity toward the target proteins.^63^ Furthermore, their binding energies are lower than those exhibited by the reference drugs (Fluconazole and Streptomycin), supporting their observed antimicrobial activity.

**Figure 6:**
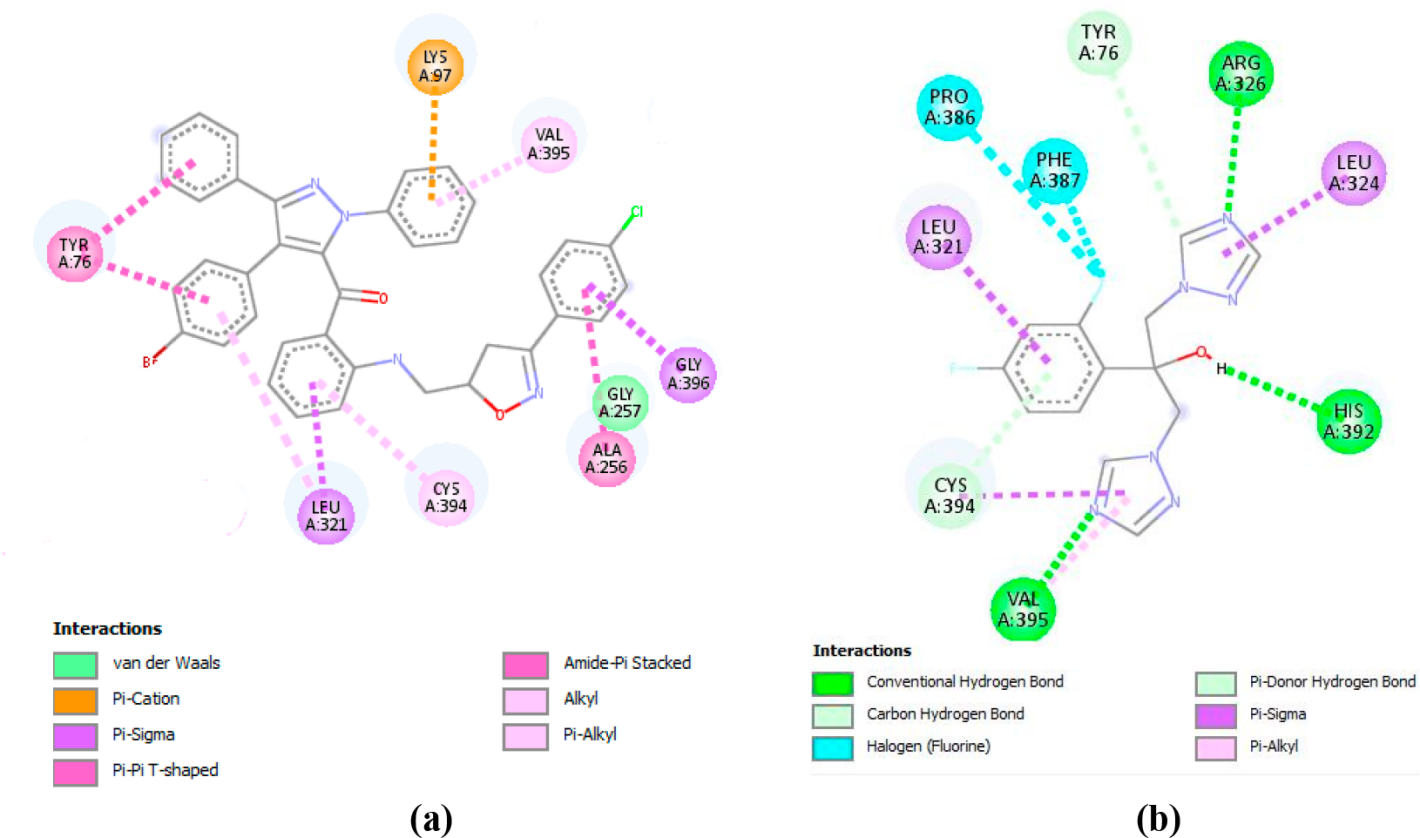
(a) 2D Diagram of **1E9X–6c** interactions. (b) 2D Diagram of interactions involved between **1E9X** and Fluconazole.

**Figure 7:**
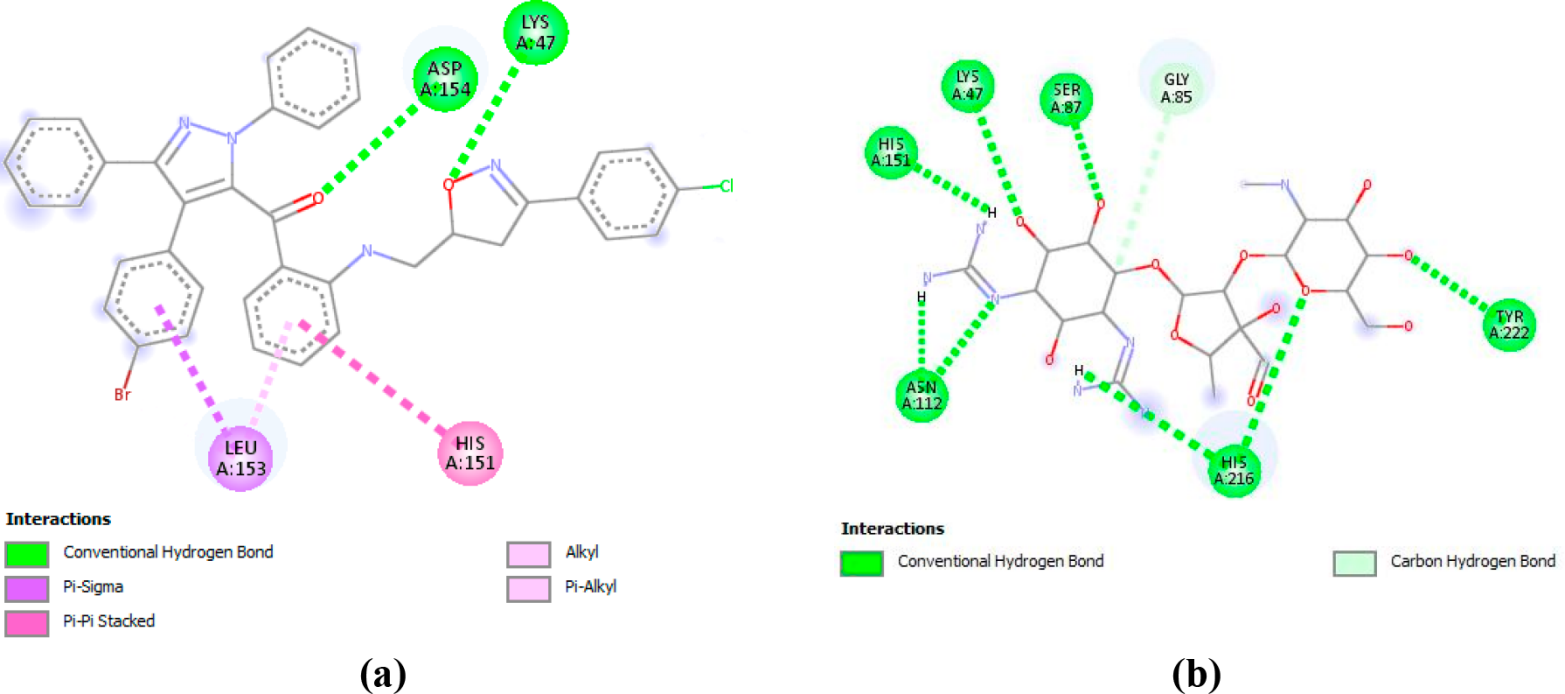
(a) 2D Diagram of **3MZD–6c** interactions. (b) 2D Diagram of interactions involved between **3MZD** and Streptomycin.

Besides, we analyzed the interactions involved between the target proteins (**1E9X** and **3MZD**), and the compound **6c** which exhibited high affinity toward **1E9X** and **3MZD** and has proven to possess good *in vitro* antimicrobial activity. The ligand–proteins interactions are depicted in Figures 6 and 7. As shown in Figure 6a, compound **6c** interact with seven residues to be complexed with **1E9X**. It implicated three alkyl interactions with VAL A: 395, CYS A: 394 and LEU A: 321, two Pi-sigma interactions with GLY A: 396 and LEU A: 321, three amide-Pi stacked interactions with TYR A: 76 and ALA A: 256, as well as a Pi-cation interaction with LYS A: 97. In addition, VAL A:395, TYR A: 76, LEU A: 321, CYS A: 364 residues with which interact compound **6c** were identified to be relevant in the complexation process between Fluconazole and **1E9X** (Figure 6b). The identification of such residues leading to the complexation of Fluconazole, which is an antifungal medicine, supports the observed antifungal activity exhibited by the studied compounds.

According to Figure 7, compound **6c** implicates two conventional hydrogen bonds with ASP A: 154 and LYS A: 74, a Pi–Pi stacked interaction with HIS A: 151, as well as Pi–sigma interaction and Pi–alkyl interaction with LEU A:153. Moreover, LEU A: 153, LYS A47, HIS A:151 are identified as the interacting residues ensuring the complexation of Streptomycin at the active site of **3MZD**. The identification of such residues leading to the complexation of Streptomycin, support the observed antibacterial activity exhibited by the studied compounds.

### 2.5 Molecular Dynamics Simulations

#### 2.5.1 Root Mean Square Deviations Analysis

**Figure 8:**
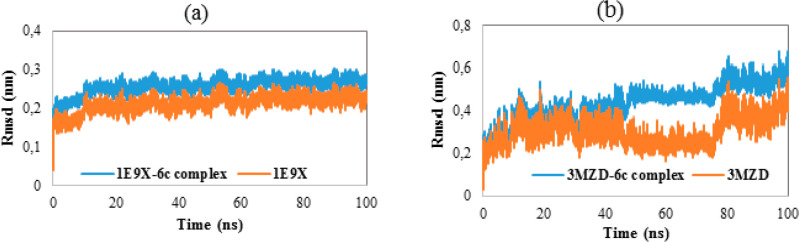
(a) RMSD of the backbone of **1E9X** and **1E9X–6c** complex as a function of time. (b) RMSD of the backbone of the **3MZD** and **3MZD–6c** complex as a function of time.

MD studies were performed to investigate the dynamic behavior of **1E9X**, **3MZD**, and their complexes with compound **6c** in an aqueous environment. At first, the stability of the studied systems under the MD conditions was evaluated by analyzing their root-mean-square deviations (RMSD) during 100 ns. Figure 8 presents the RMSD of the studied proteins as well as their respective complexes. As evident from the Figure 8a, the RMSD of **1E9X** and its complex increase until reaching stable state after 15 ns. In addition, their RMSD values do not exceed 0.3 nm during the entire simulation. However, the RMSD of **3MZD** and its complex exceeded 0.4 nm and presented more fluctuations compared to the other systems. Furthermore, the average RMSD values of **1E9X**, **3MZD**, **1E9X–6c**, and **3MZD–6c** were found to 0.212, 0.306, 0.258, and 0.436 nm, respectively. Overall, the RMSD graphs highlight the stability of **1E9X–6c** and **1E9X** in aqueous environment.

#### 2.5.2 Root Mean Square Fluctuation Analysis

**Figure 9:**
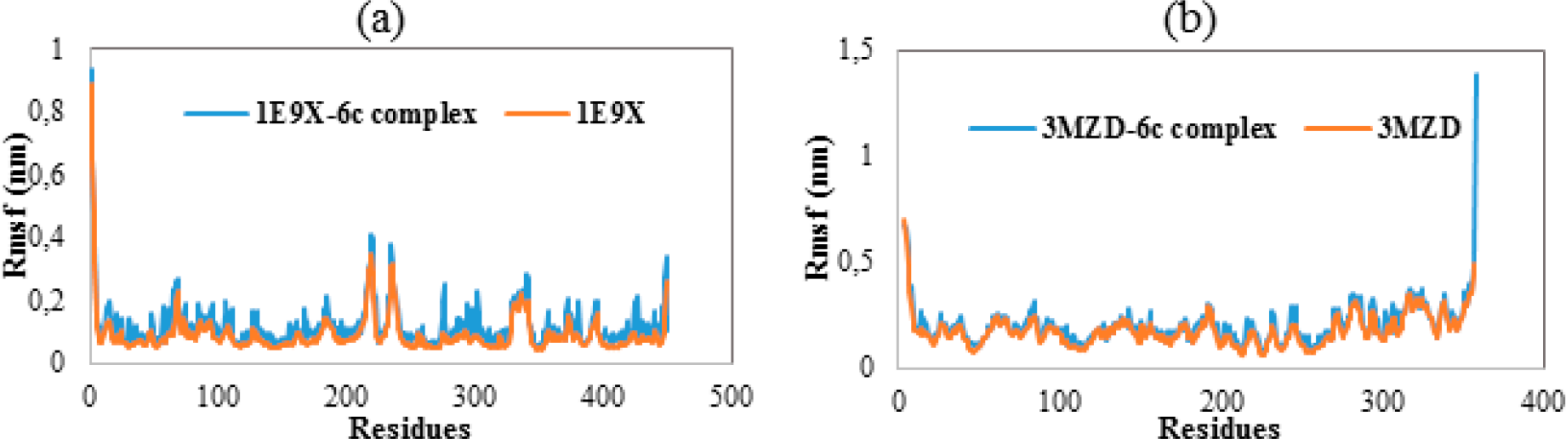
(a) RMSF of Cα atoms of **1E9X** in the absence and presence of **6c**. (b) RMSF of Cα atoms of **3MZD** in the absence and presence of **6c**.

Evaluating the root mean square fluctuation during a simulation period is an analysis performed to gain insight into the flexibility of protein residues.^64^ Indeed, more flexible residues are characterized by high RMSF values. Figure 9 shows the RMSF graphs of all investigated systems. As highlighted in Figure 9, the RMSF diagrams relative to the studied proteins are relatively comparable to those of their respective complexes, indicating that **6c** induce no potential conformational changes on its receptors. Moreover, many residues were found to have low RMSF value, reflecting the stability of the studied systems.

#### 2.5.3 Radius of Gyration

**Figure 10:**
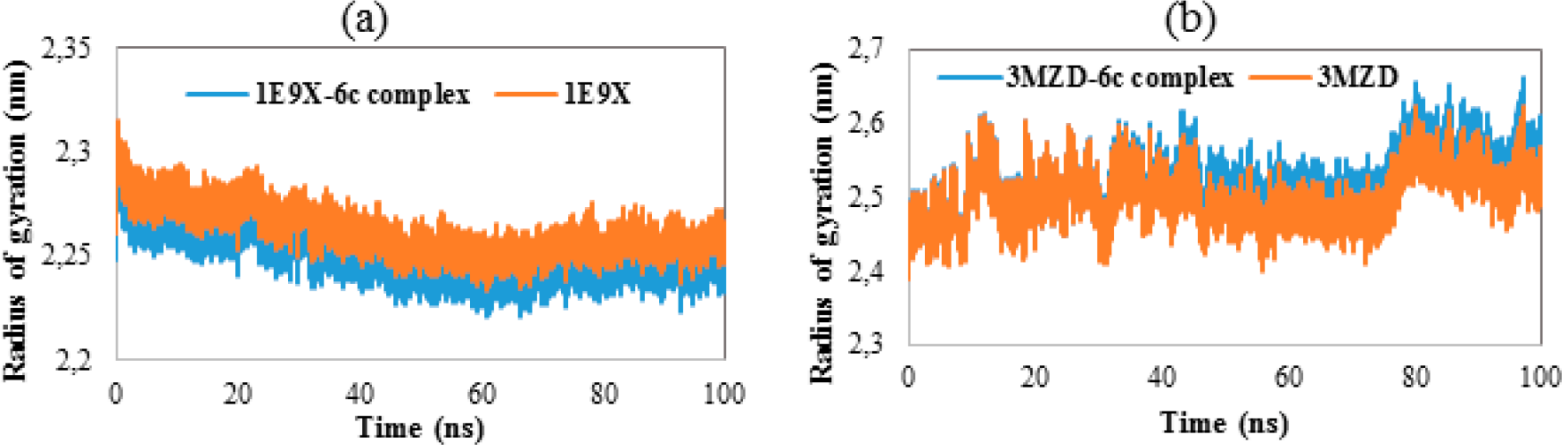
(a) RoG of **1E9X** and **1E9X–6c** complex as a function of time. (b) RoG of **3MZD** and **3MZD–6c** complex as a function of time.

The radius of gyration (RoG) diagrams corresponding to the studied systems are presented in Figure 10. The average RoG values relative to **1E9X**, **1E9X–6c** complex, **3MZD** and **3MZD–6c** complex were determined to be 2.262, 2.249, 2.502, and 2.523 nm, respectively. In addition, the variation of RoG values corresponding to **1E9X**, and its complex was revealed to be stable compared to the other systems which have shown some fluctuations during the whole simulation. The results support the stability of **1E9X–6c** in an aqueous environment.

#### 2.5.4 Number of Hydrogen Bonds

**Figure 11:**
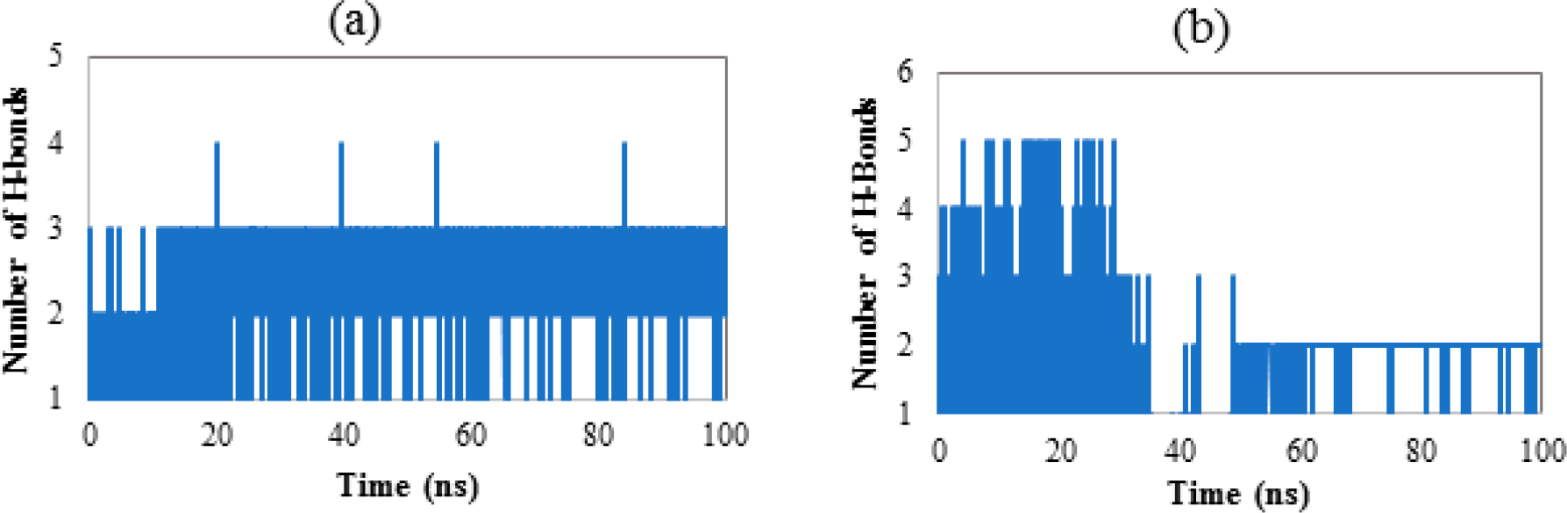
(a) Number of hydrogens formed by ligand (**6c**) with **1E9X** as a function of time. (b) The number of hydrogens formed by ligand (**6c**) with **3MZD** as a function of time.

Figure 11 reveals the number of hydrogen bonds implicated between the ligand **6c** and its receptors during the simulation period. As reveled, the ligand **6c** was able to form a great number of hydrogen bonds during the entire simulation, supporting its stability at the binding pocket of **1E9X**.

#### 2.5.5 Solvent Accessible Surface Area

**Figure 12:**
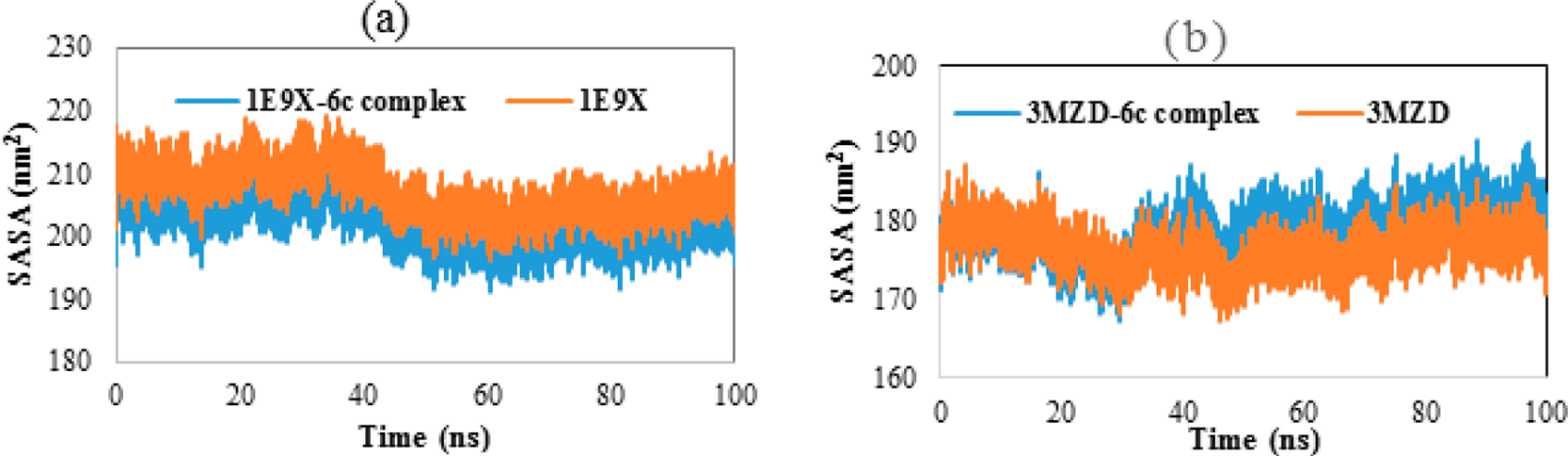
(a) SASA of **1E9X** and **1E9X–6c** complex. (b) SASA of **3MZD** and **3MZD–6c** complex.

The solvent accessible surface area (SASA) of all studied proteins as well as their complexes was calculated during the simulation period and presented in Figure 12. The obtained results reveal negligible variations in SASA values for all studied systems. Indeed, the SASA variation of all systems is almost at steady state. In addition, the SASA diagrams of the studied proteins are comparable to those of their respective complexes, indicating that the target proteins have undergone no relevant conformational change when interacting with ligand **6c**.

To sum up, all the tested parameters, including RMSD, RMSF, numbers of hydrogen bonds, SASA and RoG, confirm that compound **6c** does not influence the stability of the target proteins when it complexes with them. Besides, from the analyzed trajectories, it turned out that the **1E9X–6c** complex is likely to be more stable in an aqueous medium than **3MZD–6c** complex.

### 2.6 Pharmacokinetic Proprieties

According to Table S4, all investigated compounds are predicted to be soluble in water at 25 °C, and highly absorbable thought the human small intestine (intestinal absorption > 80%). In addition, except for compounds **6e**, **6f**, and **6g**, they are considered to have a high caco-2 permeability. Furthermore, they are likely to be skin permeable. Besides, we note that all investigated compounds are likely to penetrate the Central Nervous System (CNS) and to be moderately distributed in the brain.

Cytochrome P450 is a powerful detoxification enzyme in the body. Therefore, inhibitors of this enzyme can influence the drug metabolism process. The obtained results indicate that the investigated compounds are not likely to be inhibitors of four cytochrome P450 isoforms, namely, CYP1A2, CYP2C19, CYP2D6, and CYP3A4.

Renal OCT2 is a useful indicator of renal clearance of drugs. As revealed, none of the tested compounds is likely to be OCT2 substrate, indicating that they will difficulty transported by the Organic Cation Transporter 2. The total clearance of all tested compounds is given in log(mL/min/kg).

Toxicity evaluation is a relevant stage in drug discovery. In fact, it highlights the compounds which can present harmful effects for health. As depicted in Table S4, except for **6e**, **6f**, and **6g**, no tested compound is predicted to be mutagenic or irritant. In addition, they are not likely to be hERGI inhibitors, meaning that they are not expected to induce fatal ventricular arrhythmia by inhibiting the potassium channel encoded by Hergi (human ether-a-go-go gene).

## 3 Conclusion

To conclude, we have successfully synthesized a new class of hybrid poly heterocycles incorporating pyrazole and isoxazoline rings, using alkylation and 1,3-dipolar cycloaddition reactions. The synthesized compounds were characterized unambiguously by IR and 1D NMR and 2D NMR spectroscopy, as well as high-resolution mass spectrometry. The mechanistic study of the 1,3-dipolar reaction was investigated at the B3LYP/6-31G(d,p) level, and the observed regioselectivity was successfully explained by the analysis of the local reactivity indexes derived from the Parr functions. Furthermore, the results of the antimicrobial screening reveal that the evaluated hybrids exhibit variable and powerful antimicrobial activity against the tested pathogenic strains. The noted difference in inhibitory activity can be linked to the effect and nature of the substituents linked to the Ar and Ar_1_ rings. The potent activity obtained can be attributed to the synergistic effect between the pyrazole and isoxazoline pharmacophores. *In silico* studies were also conducted to support and provide an explanation for the outcomes found in the *in vitro* investigations. The results of the molecular docking studies revealed strong binding interactions within the *E. coli* and *C. albicans* proteins, thus proving the antimicrobial effect of the hybrid compounds. Besides that, molecular dynamics (MD) simulations proved the stability of protein–ligand interactions under physiological conditions and the binding affinity of compound **6c** with the target receptors. Interestingly, the ADMET parameters prediction indicated that the majority of the synthesized conjugates have an acceptable pharmacokinetic profile with nontoxic character. Taken together, the studied hybrids can serve as models for the design of potential new drugs, and the current research may offer a promising path toward the development of new multitargeted antimicrobial agents based on the pyrazole and isoxazoline pharmacophores.

## 4 Experimental Section

### 4.1 Chemical Reagents and Instruments

All chemicals and solvents used were of analytical quality and were obtained from commercial suppliers. They were utilized without additional purification. The different spectroscopic techniques, the detailed synthesis procedures, the physicochemical and spectroscopic data (FT-IR, ^1^H NMR, ^13^C NMR and HRMS) of the synthesized compounds are given in the Supporting Information.

### 4.2 Computational Details

Optimization of the geometries is performed with the B3LYP method,^65,66^ and the 6-31G(d,p) as a basis set using the Gaussian 09 program.^67^ The polarizable continuum model (CPCM) was applied using chloroform as a solvent. Global electronic proprieties, HOMO and LUMO, and reactivity indexes, including electronic chemical potential (μ), chemical hardness (η), global electrophilicity (ω), and global nucleophilicity *N* were computed by the following expressions: μ = (*E*_HOMO_ + *E*_LUMO_)/2 and η = (*E*_LUMO_ – *E*_HOMO_), ω = μ^2^/2η, and *N* = *E*_H_ – *E*_H_(TCE).^68,69^ The Parr functions were used to calculate the local reactivity indexes, namely, local electrophilic (P_k_^+^), and nucleophilic (P_k_^–^), via the analysis of the Mulliken atomic spin density (ASD).^70^

### 4.3 Antimicrobial Activity Assay

The target compounds were tested against four pathogenic microorganisms, namely, two Gram-positive bacteria, *S. aureus* (S. A.) (CECT 976) and *B. subtilis* (B. S.) (DSM 6633), a Gram-negative bacterial strain, *E. coli* (K12) (E. C.), and a yeast, *C. albicans* (C. A.) (ATCC 10231), using the standard agar diffusion method and the broth microdilution method. The details on the methods employed to assess the antimicrobial activity of the compounds are described in the Supporting Information.

### 4.4 Molecular Docking Analysis

Molecular docking studies were performed with the purpose of supporting the experimental results and identifying the most likely modes of interaction between the synthesized compounds and target proteins involved in antimicrobial activities. Autodock Vina software^71^ was implemented to perform the Molecular docking studies. The crystal structure of CYP51 (ID: 1E9X) as a target for antifungal compounds, was downloaded from the RCSB Protein Data Bank available online at (www.RCSB.org/structure/1E9X). CYP51 (sterol 14α-demethylase) is a cytochrome P450 enzyme essential for sterol biosynthesis and plays a crucial role in the growth of invasive fungi, making this protein one of the main targets for antifungal drug development. Several CYP51 inhibitors are used in the treatment of fungal infections, such as fluconazole, itraconazole, voriconazole.^72^

The crystal structure of penicillin-binding protein 5 from *E. coli*, was obtained from the RCSB Protein Data Bank (PDB ID: 3MZD). Penicillin-binding proteins (PBPs) are the molecular targets for the widely used β-lactam class of antibiotics.^73^

Primarily, the collected structures were prepared using AutoDockTools^74^ by adopting the following procedure:

- Step 1: Water molecules and ligands were deleted from the protein structures.
- Step 2: Kollman charges and polar hydrogen were added.
- Step 3: A docking gird box (coordinates: *x* = −16.204, *y* = −2.858, *z* = 66.913 at 30 Å size and 0.375 Å spacing) was generated to cover the active site of 1E9X. Besides, *x* = 43.068, y = 7.589, *z* = 29.610 were set as the docking center for ligands targeting the 3MZD.
- Step 4: PDBQT files corresponding to the prepared structures were generated.

Furthermore, we applied the MMFF94 force field^75^ along with the steepest Descent method (5000 steps) available in Avogadro software^76^ in order to optimize the structures of the studied compounds. Following that, Gasteiger charges were added, polar hydrogens were merged using AutoDockTools,^74^ to prepare the ligands for docking. The interactions involved between the studied compounds and the selected protein were analyzed via Discovery Studio 2021 software.

The docking protocol was validated by redocking the cocrystallized ligands and calculating the root-mean-square deviation (RMSD) corresponding to the superimposed conformation of the cocrystallized and redocked ligands in the protein target pocket.

### 4.5 Molecular Dynamics Simulation

MD simulations were done utilizing GROMACS^77^ with charmm27 force field^78^ to get an idea of the stability of the ligand–protein complexes. The ligands topologies were generated from the SwissParam server.^79^ Furthermore, the ligand–protein complexes were covered by a cubic simulation box and then were solvated with TIP3P water molecules. Sodium ions were added for neutralizing the overall system. Following that, all simulated systems were optimized using the steepest descent minimization to avoid steric clashes. Then, we performed NVT equilibration at 300 K for 1 ns, followed by NPT equilibration utilizing Parrinello–Rahman barostat at 1 atm for 1 ns,^80^ to stabilize the systems at the desired conditions. Finally, the equilibrated systems were subjected to the MD simulation for 100 ns. From the simulation results, we calculated various parameters, e.g., RMSD, RMSF, RoG, number of hydrogen bonds, and SASA.

### 4.6 Pharmacokinetic Prediction

The therapeutic effect is not the only parameter to take into consideration when developing a new drug. Indeed, evaluating the pharmacokinetic properties is a relevant stage highlighting the compounds which cannot administrated as medicine due to poor properties or harmful effects for health. In the current study, the pharmacokinetic properties of the investigated compounds, including absorption, excretion, metabolism, and toxicity were evaluated by adopting the pKCSM theory.^81,82^

## Supporting Information Available

Detailed synthesis protocol, analytical and spectroscopic data, copies of spectra (NMR, and HRMS) of the newly synthesized compounds, and all additional data analyzed during this study (PDF)
