# Supplementary material for: Synthesis, Characterization, DFT Mechanistic Study, Antimicrobial Activity, Molecular Modeling, and ADMET Properties of Novel Pyrazole-isoxazoline Hybrids
Source: ACS Omega. 2022 Dec 8;7(50):46731–44. doi: 10.1021/acsomega.2c05788 (PMC9773794; doi:10.1021/acsomega.2c05788)
Supplement: Supplementary file 1 — ao2c05788_si_001.pdf [file ao2c05788_si_001.pdf]

Supporting Information for the paper

Synthesis, characterization, DFT mechanistic study, antimicrobial activity, molecular modeling and ADMET properties of novel pyrazole-isoxazoline hybrids

Mohammed Chalkha[1], Hassan Nour[2], Khalid Chebbac[3], Asmae Nakkabi[1], Lahoucine Bahsis[4,5], Mohamed Bakhouch[6], Mohamed Akhazzane[1,7], Mohamed Bourass[8], Samir Chtita[2], Yousef A. Bin Jardan[9], Maria Augustyniak[10,*], Mohammed Bourhia[11,*], Mourad A. M. Aboul-Soud[12,*], and Mohamed El Yazidi[1]

[1] Engineering Laboratory of Organometallic, Molecular, Materials and Environment, Faculty of Sciences Dhar EL Mahraz, Sidi Mohamed Ben Abdellah University, P.O. Box 1796, 30000 Fez, Morocco

[2] Laboratory of Analytical and Molecular Chemistry, Faculty of Sciences Ben M'Sik, Hassan II University of Casablanca, P.O. Box 7955, Casablanca, Morocco.

[3] Laboratory of Biotechnology Conservation and Valorisation of Natural Resources, Faculty of Sciences Dhar El Mahraz, Sidi Mohammed Ben Abdallah University, P.O. Box 1796, Fez 30000, Morocco.

[4] Laboratory of Analytical and Molecular Chemistry, Polydisciplinary Faculty, Cadi Ayyad University, P.O. Box 4162, Safi 46000, Morocco.

[5] Department of Chemistry, Faculty of Sciences of El Jadida, Chouaïb Doukkali University, P.O. Box 20, El Jadida 24000, Morocco.

[6] Laboratory of Bioorganic Chemistry, Department of Chemistry, Faculty of Sciences, Chouaïb Doukkali University, P.O. Box 24, El Jadida 24000, Morocco.

[7] Cité de l'innovation, Université Sidi Mohamed Ben Abdellah, Route Immouzer, P.O. Box 2626, Fez, Morocco.

[8] Université de Bordeaux, CNRS, Bordeaux INP, ISM, UMR 5255, 351 Cours de la Libération, F-33405 Talence, Cédex, France.

[9] Department of Pharmaceutics, College of Pharmacy, King Saud University, Riyadh, Saudi Arabia

[10] Institute of Biology, Biotechnology and Environmental Protection, Faculty of Natural Sciences, University of Silesia in Katowice, Bankowa 9, 40-007 Katowice, Poland

[11] Higher Institute of Nursing Professions and Technical Health, Laayoune 70000, Morocco

[12] Chair of Medical and Molecular Genetics Research, Department of Clinical Laboratory Sciences, College of Applied Medical Sciences, King Saud University, P.O. Box 10219, Riyadh 11433, Saudi Arabia.

*Corresponding authors:
Mohammed Bourhia (MB) bourhiamohammed@gmail.com
Mourad A. M. Aboul-Soud (MAMA) maboulsoud@ksu.edu.sa
Maria Augustyniak (MA) maria.augustyniak@us.edu.pl

Table of Contents

Table S1 Physical and spectroscopic characterization data of the synthesized compounds **6a-m**. S3

Table S2 MIC of hybrid compounds **6a-m** S4

Table S3 Binding energy exhibited by investigated ligands **6a-m** S4

Table S4 ADMET properties of synthesized compounds **6a-m** S6

1. *Chemical reagents and instruments* S7

2. *Synthesis methods* S7

2.1 Synthetic procedure of the allylated pyrazoles **4a-d** S7

2.2 Synthetic procedure of the hybrids **6a-m** S7

3. *Antimicrobial activity* S8

3.1. Disk diffusion method S8

3.2. Minimum inhibitory concentration (MIC) S8

4. *Optimized structures of both reagents* S10

5. *Energies and cartesian coordinates for all optimized geometries* S10

6. *Characterization data of all synthesized compounds* S19

6.1. Spectroscopic data of the allylated pyrazoles **4a-d** S19

6.2. Spectroscopic data of the synthesized hybrids **6a-m** S20

7. *^{1}H, ^{13}C NMR and HRMS Spectra of hybrid molecules pyrazole-isoxazoline* ***6a-m*** S31

Figure S32. Photographs displaying the effect of the hybrid compounds on the *S. Aureus* strain S47

Figure S33. Photographs displaying the effect of the hybrid compounds on the *E. Coli* strain S47

References S48

Table S1 Physical and spectroscopic characterization data of the synthesized compounds **6a-m**.

N°	Ar_1	Ar_2	M.p[b] (°C)	NMR-^{1}H (ppm): N-C**H**$_2$ / C**H**$_2$ / **CH**	^{13}C-NMR (ppm): N-**C**H$_2$ / **C**H$_2$ / **C**H	IR(cm^{-1}): NH / C=O	HRMS *(m/z)* [M+H]+
**6a**	*p*-$CH_3(C_6H_4)$	*p*-$Cl(C_6H_4)$	164-166	3.47-3.62 (m, 2H)<br>3.15 (dd, 1H); 3.42 (dd, 1H)<br>4.97-5.07 (m, 1H)	45.66<br>38.12<br>79.53	3290<br>1622	623.22079
**6b**	*p*-$OCH_3(C_6H_4)$	*p*-$Cl(C_6H_4)$	144-146	3.48-3.62 (m, 2H)<br>3.15 (dd, 1H); 3.42 (dd, 1H)<br>4.98-5.07 (m, 1H)	45.70<br>38.15<br>79.49	3301<br>1618	639.21677
**6c**	*p*-$Br(C_6H_4)$	*p*-$Cl(C_6H_4)$	152-154	3.49-3.60 (m, 2H)<br>3.15 (dd, 1H); 3.45 (dd, 1H)<br>4.98-5.08 (m, 1H)	45.72<br>38.10<br>79.46	3300<br>1620	687.11621
**6d**	*p*-$Cl(C_6H_4)$	*p*-$Cl(C_6H_4)$	166-168	3.51-3.63 (m, 2H)<br>3.16 (dd, 1H); 3.46 (dd, 1H)<br>5.00-5.09 (m, 1H)	45.07<br>38.12<br>79.43	3300<br>1616	643.16649
**6e**	*p*-$Br(C_6H_4)$	*p*-$NO_2(C_6H_4)$	184-186	3.55-3.65 (m, 2H)<br>3.21 (dd, 1H); 3.51(dd, 1H)<br>5.07-5.17 (m, 1H)	45.69<br>37.64<br>80.30	3315<br>1614	698.14121
**6f**	*p*-$CH_3(C_6H_4)$	*p*-$NO_2(C_6H_4)$	180-182	3.45-3.64 (m, 3H)<br>3.22 (dd, 1H)<br>5.06-5.16 (m, 1H)	45.75<br>37.68<br>80.31	3315<br>1614	634.24529
**6g**	*p*-$OCH_3(C_6H_4)$	*p*-$NO_2(C_6H_4)$	170-172	3.44-3.63 (m, 3H)<br>3.22 (dd, 1H)<br>5.06-5.16 (m, 1H)	45.73<br>37.67<br>80.30	3300<br>1620	650.24097
**6h**	*p*-$Br(C_6H_4)$	*p*-$OCH_3(C_6H_4)$	136-138	3.48-3.61 (m, 2H)<br>3.14 (dd, 1H); 3.43 (dd, 1H)<br>4.97-5.07 (m, 1H)	45.77<br>38.27<br>79.17	3319<br>1616	683.1661
**6i**	*p*-$OCH_3(C_6H_4)$	*p*-$OCH_3(C_6H_4)$	172-174	3.40-3.49 (m, 2H)<br>3.16 (dd, 1H); 3.57 (dd, 1H)<br>4.93-5.03 (m, 1H)	45.83<br>38.62<br>78.86	3325<br>1610	635.26591
**6j**	*p*-$CH_3(C_6H_4)$	*p*-$CH_3(C_6H_4)$	150-152	3.40-3.52 (m, 2H)<br>3.17 (dd, 1H); 3.58 (dd, 1H)<br>4.94-5.04 (m, 1H)	45.79<br>38.45<br>79.00	3298<br>1620	603.27545
**6k**	*p*-$OCH_3(C_6H_4)$	*p*-$Br(C_6H_4)$	152-154	3.40-3.52 (m, 2H)<br>3.16 (dd, 1H); 3.44 (dd, 1H)<br>4.94-5.04 (m, 1H)	45.71<br>38.08<br>79.51	3300<br>1618	683.16602
**6l**	*p*-$OCH_3(C_6H_4)$	*p*-$CH_3(C_6H_4)$	162-164	3.41-3.58 (m, 3H)<br>3.17 (dd, 1H)<br>4.95-5.04 (m, 1H)	45.85<br>38.48<br>78.55	3305<br>1618	619.27075
**6m**	*p*-$Cl(C_6H_4)$	*o*-$Cl(C_6H_4)$	180-190	3.49-3.59 (m, 2H)<br>3.36 (dd, 1H); 3.65 (dd, 1H)<br>5.02-5.11 (m, 1H)	45.64<br>40.68<br>79.70	3305<br>1620	643.16668

Table S2 MIC of hybrid compounds **6a-m**

**MIC in *μM* (means ± SD, *n* = 2)**						
**Compounds**			**Tested microorganisms**			
**N°**	Ar_1	Ar_2	***E. Coli***	***B. Subtilis***	***C. Albicans***	***S. Aureus***
**6a**	*p*-$CH_3(C_6H_4)$	*p*-$Cl(C_6H_4)$	200.8 ±1.16	602.7 ±2.17	200.8 ±0.42	200.8 ±2.44
**6b**	*p*-$OCH_3(C_6H_4)$	*p*-$Cl(C_6H_4)$	97.9 ±0.33	NT	195.8 ±0.65	391.72 ±3.45
**6c**	*p*-$Br(C_6H_4)$	*p*-$Cl(C_6H_4)$	91.1 ±0.66	**34.14 ±0.11**	**91.1 ±0.33**	273.3 ±0.88
**6d**	*p*-$Cl(C_6H_4)$	*p*-$Cl(C_6H_4)$	97.3 ±0.77	194.6 ±1.32	291.9 ±0.88	**18.23 ±0.55**
**6e**	*p*-$Br(C_6H_4)$	*p*-$NO_2(C_6H_4)$	358.6 ±1.22	NT	179.3 ±1.55	358.6 ±0.43
**6f**	*p*-$CH_3(C_6H_4)$	*p*-$NO_2(C_6H_4)$	98.7 ±0.42	789.6 ±2.41	197.4 ±2.73	592.2 ±1.77
**6g**	*p*-$OCH_3(C_6H_4)$	*p*-$NO_2(C_6H_4)$	**48.1 ±0.63**	NT	**96.2 ±0.82**	**48.1 ±0.53**
**6h**	*p*-$Br(C_6H_4)$	*p*-$OCH_3(C_6H_4)$	366.5 ±2.15	**68.7 ±0.22**	**91.6 ±1.24**	274.8 ±0.93
**6i**	*p*-$OCH_3(C_6H_4)$	*p*-$OCH_3(C_6H_4)$	**49.2 ±0.23**	591.24 ±1.76	295.6 ±0.88	394.1 ±0.89
**6j**	*p*-$CH_3(C_6H_4)$	*p*-$CH_3(C_6H_4)$	415.1 ±2.67	415.1 ±1.73	103.77 ±0.91	311.3±1.10
**6k**	*p*-$OCH_3(C_6H_4)$	*p*-$Br(C_6H_4)$	68.7 ±0.22	**91.6 ±0.76**	137.3 ±0.44	183.2 ±0.95
**6l**	*p*-$OCH_3(C_6H_4)$	*p*-$CH_3(C_6H_4)$	75.8 ±0.82	404.3 ±0.94	**101.1 ±0.87**	404.3 ±1.34
**6m**	*p*-$Cl(C_6H_4)$	*o*-$Cl(C_6H_4)$	194.6 ±0.54	583.9 ±1.76	194.6 ±1.62	**36.5 ±0.88**
**Ampicillin**			NT	22.35 ±0.76	NT	11.1 ±0.43
**Fluconazole**			NT	NT	51 ±0.42	NT
**Streptomycin**			13.4 ±0.52	NT	NT	NT

* Values are represented as mean ± standard deviations of twice experiments.

Table S3 Binding energy exhibited by investigated ligands **6a-m**

**Anti-fungal activity**			**Anti-biotic activity**		
Ligands	Binding energy (kcal/mol)	Interacting Residues	Ligands	Binding energy (kcal/mol)	Interacting Residues
**6a**	**-13.2**	GLY396/ALA256 CYS394/LEU321 HIS259/VAL434 MET79/TYR76 LYS97/VAL395 PHE74	**6a**	**-8.9**	ASP 154/ LEU 153 LYS 47 / GLY 152
**6b**	**-12.8**	GLY396/ALA256 CYS394/LEU321 HIS259/VAL434 MET79/TYR76 LYS97/VAL395 GLY257	**6b**	**-8.8**	ASP 154/ LEU 153 LYS 47 / HIS 151
**6c**	**-13.2**	GLY396/ALA256 CYS394/LEU321	**6c**	**-9.1**	ASP 154/ LYS 47

		TYR76/LYS97 VAL395			HIS 151 /LEU 153
**6d**	**-13.1**	GLY396/ALA256 CYS394/LEU321 TYR76/LYS97 VAL395	**6d**	**-8.9**	ASP 154 /LYS 47 HIS 151/ LEU 153 GLY 152
**6e**	**-13.1**	GLY396/ALA256 CYS394/LEU321 TYR76 /LYS97 VAL395	**6e**	**-9.1**	ASP 154 /LYS 47 HIS 151/ LEU 153 GLY 152/ARG198
**6f**	**-13.0**	GLY396/ALA256 CYS394/LEU321 PHE78/TYR76 LYS97/VAL395	**6f**	**-9.3**	ASP 154/ LYS 47 HIS 151/ LEU 153 ARG198/GLY 152
**6g**	**-12.6**	GLY396/ALA256 CYS394/LEU321 TYR76/LYS97 VAL395/PHE78	**6g**	**-9.2**	ASP 154 /LYS 47 HIS 151/LEU 153 VAL 82/ ARG 198
**6h**	**-12.7**	GLY396/ALA256 VAL395/LYS97 TYR76/LEU321 CYS394/LEU105 LEU152/HIS101	**6h**	**-8.9**	ASP 154 /LYS 47 HIS 151/LEU 153
**6i**	**-11.4**	PHE78/MET79 PHE255/HIS259 VAL434/TYR76 ALA256/GLN72 ALA73/LYS97 ARG393/VAL395 CYS394/LEU321	**6i**	**-8.8**	ASP 154 /LYS 47 HIS 151/LEU 153
**6j**	**-13.4**	GLY396/ALA256 CYS394/LEU321 TYR76/LYS97 VAL395	**6j**	**-8.9**	ASP 154 /LYS 47 GLY 152/LEU 153
**6k**	**-12.8**	GLY396/ALA256 CYS394/LEU321 TYR76/LYS97 VAL395/PHE78	**6k**	**-8.9**	ASP 154 /LYS 47 HIS 151/LEU 153 GLY 152
**6l**	**-13.0**	GLY396/ALA256 CYS394/LEU321 HIS259/VAL434 TYR76/LYS97 VAL395/PHE78	**6l**	**-8.9**	ASP 154 /LYS 47 HIS 151/LEU 153
**6m**	**-13.3**	GLY396/LEU105 ALA256/CYS394 LEU321/MET79 TYR76/LYS97 VAL395	**6m**	**-9.2**	ASP 154 /LYS 47 HIS 151/ LEU 153 GLY 152
**Fluconazole**	**-7.3**	LEU321/PRO386 PHE387/TYR76 ARG326/LEU324 HIS392/VAL395 CYS394	**Streptomycin**	**-8.4**	HIS 151/ LYS 47 SER 87/ GLY 85 TYR 222/ HIS 216 ASN 112

Table S4 ADMET properties of synthesized compounds **6a-m**

Property	Model Name	Predicted value												
		**6a**	**6b**	**6c**	**6d**	**6e**	**6f**	**6g**	**6h**	**6i**	**6j**	**6k**	**6l**	**6m**
**Absorption**	Water solubility	-2.99	-2.98	-2.99	-2.99	-2.95	-2.96	-2.94	-2.99	-2.98	-3.00	-2.98	-2.98	-2.99
	Caco2 permeability	0.93	0.95	0.91	0.92	-0.37	-0.26	-0.59	0.93	0.97	0.94	0.95	0.96	0.92
	Intestinal absorption	91.13	92.04	89.60	89.67	100	100	100	91.15	93.59	92.58	91.97	93.49	89.67
	Skin permeability	-2.73	-2.73	-2.73	-2.73	-2.73	-2.73	-2.73	-2.73	-2.73	-2.73	-2.73	-2.73	-2.73
**Distribution**	BBB permeability	-0.58	-0.80	-0.7	-0.75	-0.57	-0.39	-0.60	-0.82	-0.86	-0.40	-0.81	-0.63	-0.75
	CNS permeability	-1.31	-1.59	-1.25	-1.27	-1.55	-1.61	-1.89	-1.51	-1.85	-1.35	-1.56	-1.63	-1.27
**Metabolism**	CYP1A2 inhibitor	No	No	No	No	No	No	No	No	No	No	No	No	No
	CYP2C19 inhibitor	No	No	No	No	No	No	No	No	No	No	No	No	No
	CYP2C9 inhibitor	Yes	Yes	Yes	Yes	Yes	Yes	Yes	Yes	Yes	Yes	Yes	Yes	Yes
	CYP2D6 inhibitor	No	No	No	No	No	No	No	No	No	No	No	No	No
	CYP3A4 inhibitor	No	No	No	No	No	No	No	No	No	No	No	No	No
**Excretion**	Total Clearance	0.06	0.14	-0.03	-0.01	0.12	0.49	0.49	0.13	0.47	0.48	0.12	0.47	0.05
	OCT2 substrate	No	No	No	No	No	No	No	No	No	No	No	No	No
**Toxicity**	AMES toxicity	No	No	No	No	Yes	Yes	Yes	No	No	No	No	No	No
	hERGI inhibitor	No	No	No	No	No	No	No	No	No	No	No	No	No
	LD50	2.97	2.86	2.97	2.98	2.61	4.39	2.58	2.97	2.86	2.97	2.86	2.86	2.97
	Skin Sensitization	No	No	No	No	No	No	No	No	No	No	No	No	No

1. *Chemical reagents and instruments*

All chemicals used were of analytical grade and were used without further purification and were purchased from commercial suppliers. The progress of the reactions was monitored by TLC (Merck, silica gel 60 F254), and spots were visualized under UV light (VILBER LOURMAT, VL-215.LC). Column chromatography was performed using Merck silica gel (70-230 mesh) and n-hexane / diethyl ether mixture as eluent. The melting points were determined with an uncertainty of ± 2 °C using a KOFLER BENCH. The IR spectra were recorded in the range of 450–4000 cm^{-1} on a BRUKER VERTEX 70 FT-IR Spectrometer, and wavenumbers are given in cm^{-1}. The NMR spectra (^1H and ^{13}C) were recorded at room temperature on a BRUKER AVANCE II 300 Ultra-Shield (300 MHz for ^1H and 75 MHz for ^{13}C) spectrometer using $CDCl_3$ as solvent. For the ^{13}C NMR spectra, the APT experiment was used, which provide information on the multiplicity of the ^{13}C signals (CH_3, CH_2, CH and C_q). In these spectra, the negative signals correspond to the CH_3 and CH carbons and the positive signals correspond to the CH_2 and quaternary C carbons. The chemical shifts are expressed in ppm and the coupling constants *J* are expressed in Hertz (Hz). The spin multiplicities are reported as singlet (s), doublet (d), triplet (t), multiplet (m), doublet of doublets (dd), doublet of triplets (dt) and broad (br). High-resolution mass spectra were recorded on a Waters/Vion IMS-QTOF: Spectrometer, equipped with an electrospray ionization (ESI), source operating in either positive and negative ion mode. The arylhydroxamoyl chlorides **5a-f** as arylnitriloxide precursors were prepared according to the method described in literature.[1–3]

2. *Synthesis methods*

2.1 Synthetic procedure of the allylated pyrazoles 4a-d[4]

In a 100 ml flask, 1 mole of pyrazoles **3**, sodium hydride NaH and 0.5 mole of BTBA were dissolved in 20 ml of DMF. The mixture was stirred at room temperature for 15 min, and 1 mole of allyl bromide was added. The reaction mixture was kept under magnetic stirring until the consumption of the reagents as indicated by TLC. Once the reaction was completed, the solvent was distilled under vacuo, and the obtained residue was diluted with DCM dichloromethane, washed with water and the organic layer was dried over anhydrous Na_2SO_4, filtered, and concentrated. The crude product was purified by silica gel column chromatography using a mixture of hexane/diethyl ether as eluent to give allylated pyrazoles **4a-d** in good yield.

2.2 Synthetic procedure of the hybrids 6a-m

In a 100 ml flask equipped with a condenser and a $CaCl_2$ guard tube, 1 mol of dipolarophile **4a-d** and 1.2 mol of arylhydroxamoyl chloride **5a-f** are dissolved in 20 ml of chloroform. To this

mixture, 1.2 mol of anhydrous triethylamine were added slowly under magnetic stirring. After the addition, the stirring was continued at room temperature for the appropriate period of time, and the progress of the reaction was monitored by TLC. Then, the reaction mixture was transferred into a separatory funnel and extracted with chloroform, washed three times with water. The organic layer was dried over anhydrous sodium sulfate (Na_2SO_4), which was filtered off and the solvent was removed by rotary evaporator. The obtained residue was purified by silica gel column chromatography (eluent: hexane/ether (4/1)), the obtained product was crystallized in ethanol.

3. *Antimicrobial activity*

3.1. Disk diffusion method

The antimicrobial activities of the pyrazole-isoxazoline hybrids against *Staphylococcus aureus* (CECT 976) and *Bacillus subtilis* (DSM 6633), *Escherichia coli* (K12), and *Candida albicans* (ATCC 10231) were assessed via the conventional agar disk diffusion technique, according to protocols published in the literature with minor modifications.[5–8] Stock solutions of synthesized hybrid heterocycles were prepared in 1 mL of anhydrous DMF to obtain the necessary concentration (1 mg/mL). Then, 30 µL of the compounds were added to the Whatman Filter Paper disk (6 mm diameter) and dried for 10 min. Ampicillin and streptomycin were used as standard antibacterial agents, while Fluconazole was used as a standard agent for antifungal activity. DMF alone was used as a control, and it showed no effect on the growth of microorganisms. After that, the plates were incubated for 24 hours at 37 °C for the bacteria and 30 °C for yeast. Lastly, a transparent ruler was used to measure the zone of inhibition surrounding the disk (in mm). The percentage activity index of the examined compounds was calculated using the equation shown below:[9,10]

$$\text{Activity index (A)} = \frac{\text{Inhibition zone of hybrid compound (mm)}}{\text{Inhibition zone of standard drug (mm)}} \times 100$$

3.2. Minimum inhibitory concentration (MIC)

The *in vitro* screening of the pyrazole-isoxazoline hybrids against bacterial and fungi strains was carried out using the broth microdilution method in 96-well plates, according to the previously described methods,[4,11] and following guidelines of the Clinical and Laboratory Standards Institute (CLSI, approved Standard M7-A8 and M27-A3).[12,13] The tested hybrids compounds were dissolved in DMF and mixed with sterile LB medium. From a stock solution of the tested compounds and reference drugs dissolved in DMF, the required concentrations of 1000, 500, 250, 125, 62.5, 31.25, 15.62, 7.81, 3.90 and 1.95 µg/mL were prepared by the

successive dilution ½. Each well was inoculated by 100 µL of Luria Bertani medium liquid culture medium (LB) for bacteria and Malt Extract (ME) liquid culture medium for fungi,[4] more than 50 µL of the tested compounds; every well was then inoculated with 50 µL of the microbial concentration. Negative controls (bacteria and fungi without drugs) and positive controls (bacteria, fungus, and serially diluted antibiotics) were included on every plate. After incubation at an appropriate temperature and period, the results were recorded visually in terms of minimum inhibitory concentration (MIC, µM). The experiments were repeated twice, and the average results were calculated for possible antimicrobial activity.

4. *Optimized structures of both reagents*

1)

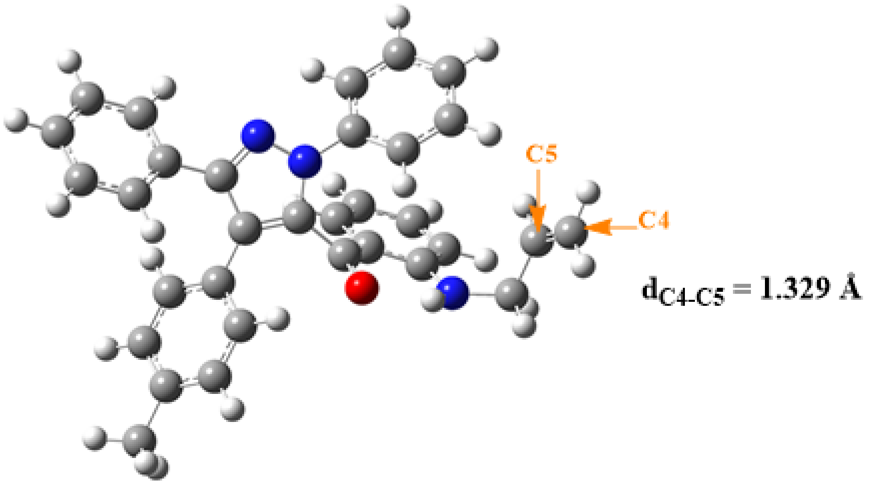

2)

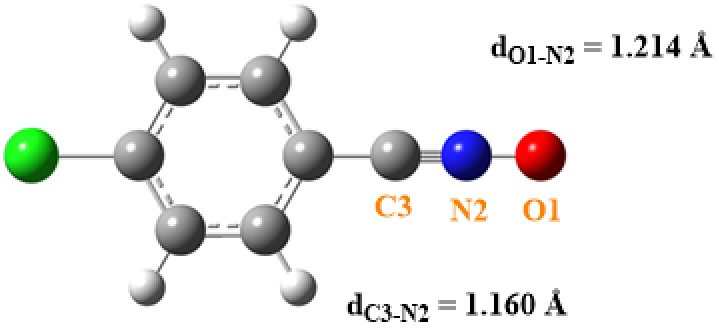

Figure S1. Optimized structures of 1) the allylated pyrazole (**4a**) and 2) the nitrile oxide (**5a**) reagents

5. *Energies and cartesian coordinates for all optimized geometries*

Table S5 Allylated pyrazole (4a): Energy: -1474.174428 Hartree

Center Number	Atomic Number	Coordinates (Angstroms)		
		X	Y	Z
1	6	-0.168053	-0.109106	-0.291321
2	6	-1.544128	0.025222	-0.25727
3	6	-2.02188	-1.316087	-0.239439
4	7	0.103266	-1.445933	-0.298837
5	7	-1.016367	-2.186897	-0.275807
6	6	1.376871	-2.072722	-0.389746
7	6	1.646182	-3.181251	0.407852
8	6	2.341452	-1.562393	-1.255697
9	6	2.89998	-3.778131	0.339025
10	1	0.87875	-3.557559	1.074229
11	6	3.598948	-2.154531	-1.299957

12	1	2.113552	-0.714616	-1.892417
13	6	3.880876	-3.262818	-0.506173
14	1	3.114917	-4.642065	0.958949
15	1	4.352722	-1.748083	-1.965819
16	1	4.860264	-3.727222	-0.548575
17	6	-3.412447	-1.810128	-0.219288
18	6	-4.430705	-1.133104	-0.899361
19	6	-3.720437	-2.991926	0.462955
20	6	-5.730986	-1.626492	-0.890341
21	1	-4.201308	-0.221789	-1.441453
22	6	-5.020107	-3.486112	0.466167
23	1	-2.931009	-3.518541	0.989169
24	6	-6.030149	-2.803296	-0.207718
25	1	-6.511431	-1.093231	-1.423739
26	1	-5.245287	-4.404685	0.998672
27	1	-7.044924	-3.188255	-0.202798
28	6	-2.313557	1.280566	-0.159516
29	6	-2.101771	2.326874	-1.062385
30	6	-3.278705	1.447276	0.839793
31	6	-2.832866	3.505628	-0.962804
32	1	-1.355615	2.211976	-1.840889
33	6	-4.008618	2.625664	0.929923
34	1	-3.45866	0.644767	1.548638
35	6	-3.79724	3.676615	0.03257
36	1	-2.654342	4.30421	-1.677929
37	1	-4.755146	2.73213	1.712372
38	6	-4.565016	4.968054	0.153877
39	1	-4.02292	5.682823	0.782852
40	1	-4.711739	5.437073	-0.822632
41	1	-5.545694	4.808094	0.609385
42	6	0.91436	0.923411	-0.232931
43	8	1.019685	1.718835	-1.168187
44	6	1.825623	0.874543	0.911085
45	6	3.139995	1.4234	0.822381
46	6	1.437917	0.185553	2.076902
47	6	4.022988	1.189101	1.90211
48	6	2.300451	0.010408	3.13732
49	1	0.429585	-0.21263	2.133236
50	6	3.60535	0.50867	3.027437
51	1	5.039164	1.561607	1.855785
52	1	1.980362	-0.512581	4.03071
53	1	4.309004	0.362288	3.841278
54	7	3.531477	2.134101	-0.271969
55	1	2.828704	2.207221	-0.997598
56	6	4.919269	2.298878	-0.673479
57	1	4.939681	3.036029	-1.479687
58	1	5.492828	2.730861	0.15431
59	6	5.54666	1.007062	-1.13344

60	1	5.521343	0.186826	-0.41702
61	6	6.092124	0.824556	-2.331918
62	1	6.122566	1.621226	-3.071335
63	1	6.534234	-0.125742	-2.61558

Table S6 Nitrile oxide (5a): Energy: -859.245608 Hartree

Center Number	Atomic Number	Coordinates (Angstroms)		
		X	Y	Z
1	6	-1.124376	1.214133	0.000073
2	6	0.263488	1.213395	-0.000051
3	6	0.964216	0.000715	0.000057
4	6	0.264142	-1.212392	0.000146
5	6	-1.123695	-1.213896	-0.000128
6	6	-1.803686	-0.000059	-0.000047
7	1	-1.671621	2.149229	0.000114
8	1	0.804522	2.152484	0.000017
9	1	0.805725	-2.151163	0.000246
10	1	-1.670439	-2.149285	-0.000099
11	6	2.396434	0.001033	0.000158
12	7	3.556703	-0.000191	-0.000028
13	8	4.771482	-0.000951	-0.000119
14	17	-3.550359	-0.000582	-0.000023

Table S7 TSA: Energy: -2333.383235 Hartree

Center Number	Atomic Number	Coordinates (Angstroms)		
		X	Y	Z
1	6	2.896776	0.785025	-0.34415
2	6	4.016157	-0.064348	-0.39113
3	6	5.162207	0.806357	-0.338263
4	7	3.389813	2.09469	-0.277495
5	7	4.746908	2.110966	-0.256853
6	6	2.641448	3.330554	-0.214692
7	6	3.044044	4.328946	0.688741
8	6	1.541942	3.519586	-1.066881
9	6	2.318444	5.520702	0.744959
10	1	3.917095	4.17696	1.325526
11	6	0.827332	4.716601	-0.989492
12	1	1.249051	2.742419	-1.783269
13	6	1.210466	5.714608	-0.086978
14	1	2.620734	6.302475	1.441346
15	1	-0.030878	4.872024	-1.643197
16	1	0.648356	6.645713	-0.035091
17	6	6.582611	0.44986	-0.373786
18	6	7.031938	-0.565647	-1.235059

19	6	7.501653	1.129032	0.443933
20	6	8.386756	-0.902266	-1.267132
21	1	6.321109	-1.088065	-1.876365
22	6	8.855258	0.785478	0.405312
23	1	7.155505	1.928117	1.099377
24	6	9.299541	-0.230122	-0.447166
25	1	8.732327	-1.689466	-1.934959
26	1	9.56538	1.313267	1.039297
27	1	10.35428	-0.495256	-0.475252
28	6	4.003611	-1.520396	-0.464635
29	6	3.165275	-2.174922	-1.382445
30	6	4.823714	-2.282589	0.386274
31	6	3.147023	-3.568962	-1.448384
32	1	2.529079	-1.590448	-2.04869
33	6	4.802776	-3.674644	0.319792
34	1	5.479951	-1.779584	1.096893
35	6	3.963924	-4.329559	-0.598183
36	1	2.492885	-4.064049	-2.163023
37	1	5.439114	-4.256316	0.983523
38	6	3.944931	-5.822834	-0.655068
39	1	3.501404	-6.244395	0.258848
40	1	3.363797	-6.207789	-1.502666
41	1	4.959044	-6.236219	-0.74576
42	6	1.4527	0.403792	-0.379573
43	8	0.814844	0.645715	-1.401669
44	6	0.87733	-0.234897	0.795677
45	6	-0.486903	-0.672385	0.820422
46	6	1.665468	-0.436173	1.959016
47	6	-1.010523	-1.256817	2.018279
48	6	1.149203	-1.015039	3.099536
49	1	2.71318	-0.119779	1.945812
50	6	-0.205367	-1.423381	3.121599
51	1	-2.05705	-1.563002	2.055171
52	1	1.768584	-1.161704	3.978983
53	1	-0.609173	-1.872322	4.031238
54	7	-1.270938	-0.553691	-0.306978
55	1	-0.902224	-0.047708	-1.132123
56	6	-2.698456	-0.931839	-0.359158
57	1	-2.957808	-1.131592	-1.425543
58	1	-2.865346	-1.898409	0.181579
59	6	-3.585823	0.145639	0.22801
60	6	-4.767576	0.507272	-0.366599
61	1	-5.114682	0.069011	-1.290704
62	6	-6.253902	-0.596195	0.792666
63	7	-5.471815	-1.06691	1.597008
64	8	-4.260036	-1.014521	1.764243
65	1	-3.136001	0.751383	1.019744

66	6	-7.576403	-0.423574	0.296947
67	6	-8.352811	0.667373	0.73634
68	6	-8.093333	-1.324598	-0.655322
69	6	-9.640969	0.853073	0.233733
70	1	-7.944493	1.365331	1.469349
71	6	-9.381167	-1.142152	-1.1599
72	1	-7.485385	-2.16401	-0.996357
73	6	-10.135699	-0.055529	-0.706909
74	1	-10.252184	1.692915	0.567803
75	1	-9.793065	-1.834336	-1.895866
76	17	-11.72526	0.171817	-1.32887
77	1	-5.270969	1.428838	-0.107181

Table S8 Regio-isomer 6a: Energy: -2333.509208 Hartree

Center Number	Atomic Number	Coordinates (Angstroms)		
		X	Y	Z
1	6	2.887857	0.804783	-0.296973
2	6	3.983778	-0.072865	-0.380264
3	6	5.153293	0.765114	-0.323157
4	7	3.417506	2.099023	-0.20683
5	7	4.773915	2.078098	-0.204254
6	6	2.704697	3.352873	-0.099249
7	6	3.140711	4.309433	0.833448
8	6	1.607487	3.601111	-0.938974
9	6	2.450995	5.519646	0.931487
10	1	4.011992	4.111812	1.460238
11	6	0.92953	4.815966	-0.820294
12	1	1.288816	2.855361	-1.677188
13	6	1.346027	5.772819	0.11152
14	1	2.779334	6.269649	1.650895
15	1	0.074757	5.018601	-1.46522
16	1	0.812663	6.718557	0.195489
17	6	6.562967	0.371908	-0.386939
18	6	6.972566	-0.637416	-1.274831
19	6	7.511302	1.009792	0.430593
20	6	8.317303	-1.009133	-1.333475
21	1	6.239309	-1.127744	-1.916151
22	6	8.854515	0.631304	0.36523
23	1	7.196271	1.804331	1.106947
24	6	9.259257	-0.378213	-0.513765
25	1	8.632232	-1.791559	-2.02187
26	1	9.587536	1.127097	0.999125
27	1	10.306114	-0.670635	-0.56261
28	6	3.931586	-1.525938	-0.488529
29	6	3.072953	-2.135537	-1.419115

30	6	4.733192	-2.329649	0.34059
31	6	3.016681	-3.525978	-1.517605
32	1	2.452513	-1.518257	-2.06984
33	6	4.67409	-3.719273	0.241627
34	1	5.405914	-1.861956	1.05996
35	6	3.815718	-4.329047	-0.688198
36	1	2.347782	-3.987564	-2.241024
37	1	5.296705	-4.332377	0.890125
38	6	3.742631	-5.81857	-0.781583
39	1	2.991024	-6.213581	-0.081595
40	1	3.461688	-6.161345	-1.786096
41	1	4.698909	-6.298843	-0.53516
42	6	1.436249	0.46119	-0.321216
43	8	0.777282	0.779388	-1.308853
44	6	0.871139	-0.241133	0.824974
45	6	-0.489416	-0.686067	0.837307
46	6	1.668361	-0.499886	1.969817
47	6	-1.00036	-1.346953	1.996843
48	6	1.163343	-1.147894	3.078953
49	1	2.713142	-0.173248	1.969223
50	6	-0.18541	-1.570679	3.085205
51	1	-2.043251	-1.665878	2.025789
52	1	1.790191	-1.337758	3.945447
53	1	-0.580333	-2.077023	3.968706
54	7	-1.292067	-0.47663	-0.268286
55	1	-0.920433	0.056835	-1.074695
56	6	-2.691811	-0.922391	-0.358113
57	1	-2.972019	-0.979436	-1.434465
58	1	-2.804833	-1.964083	0.041142
59	6	-3.642161	0.006383	0.417641
60	6	-4.897339	0.454863	-0.346342
61	1	-4.890929	0.165631	-1.412321
62	6	-6.007031	-0.256909	0.419666
63	7	-5.533846	-0.93768	1.439447
64	8	-4.181789	-0.825464	1.543367
65	1	-3.113437	0.834861	0.929777
66	6	-7.422408	-0.191461	0.049406
67	6	-7.873211	0.760985	-0.882533
68	6	-8.342193	-1.08681	0.629784
69	6	-9.222784	0.819369	-1.235501
70	1	-7.175895	1.465202	-1.3347
71	6	-9.69298	-1.035573	0.282997
72	1	-7.997183	-1.825304	1.359366
73	6	-10.113422	-0.081962	-0.647236
74	1	-9.576349	1.556572	-1.956737
75	1	-10.407379	-1.72717	0.73131

76	17	-11.7803	-0.014322	-1.082515
77	1	-5.028472	1.552438	-0.31694

Table S9 TSB: Energy: -2333.370269 Hartree

Center Number	Atomic Number	Coordinates (Angstroms)		
		X	Y	Z
1	6	-1.371059	-0.419771	-0.698149
2	6	-2.657925	0.102439	-0.470286
3	6	-3.537216	-1.035015	-0.460383
4	7	-1.5164	-1.805019	-0.825049
5	7	-2.811231	-2.181881	-0.670402
6	6	-0.491051	-2.795511	-1.075645
7	6	-0.448355	-3.954836	-0.284074
8	6	0.423084	-2.589829	-2.121127
9	6	0.528738	-4.916858	-0.550853
10	1	-1.168609	-4.104707	0.522494
11	6	1.392228	-3.563909	-2.369461
12	1	0.379906	-1.680845	-2.730902
13	6	1.445393	-4.725085	-1.590155
14	1	0.572838	-5.823111	0.05621
15	1	2.107361	-3.417981	-3.181288
16	1	2.201793	-5.484079	-1.795565
17	6	-4.990166	-1.061222	-0.276163
18	6	-5.796396	-0.116997	-0.934485
19	6	-5.581473	-2.031033	0.55054
20	6	-7.181787	-0.144385	-0.761446
21	1	-5.339022	0.635965	-1.577395
22	6	-6.968487	-2.050546	0.718012
23	1	-4.958204	-2.766998	1.05895
24	6	-7.769502	-1.109107	0.064084
25	1	-7.806305	0.588466	-1.272463
26	1	-7.425864	-2.80311	1.360381
27	1	-8.850859	-1.127038	0.196614
28	6	-3.011951	1.502869	-0.272275
29	6	-3.832022	1.881027	0.805864
30	6	-2.531259	2.487102	-1.151625
31	6	-4.161899	3.220928	1.000005
32	1	-4.210439	1.120805	1.490483
33	6	-2.864442	3.828148	-0.955963
34	1	-1.899429	2.202963	-1.99391
35	6	-3.68097	4.207332	0.120941
36	1	-4.796142	3.505836	1.838818
37	1	-2.487243	4.582228	-1.645858
38	6	-4.041969	5.638559	0.339411
39	1	-3.595504	6.312671	-0.403842

40	1	-5.13104	5.789886	0.294568
41	1	-3.713472	5.990778	1.328722
42	6	-0.081986	0.328505	-0.813444
43	8	0.443414	0.392774	-1.92532
44	6	0.467528	0.947306	0.37689
45	6	1.674979	1.723801	0.335637
46	6	-0.185162	0.791416	1.631858
47	6	2.162047	2.314236	1.552072
48	6	0.301331	1.363789	2.784805
49	1	-1.103245	0.195999	1.675746
50	6	1.490347	2.135921	2.735762
51	1	3.079372	2.903849	1.52094
52	1	-0.212653	1.234784	3.733741
53	1	1.866272	2.587369	3.657488
54	7	2.332811	1.923179	-0.848741
55	1	1.983739	1.468713	-1.712042
56	6	3.615783	2.657932	-0.984708
57	1	3.395419	3.648469	-1.458494
58	1	4.053309	2.890485	0.017346
59	6	4.594188	1.88517	-1.831067
60	6	5.718707	2.501126	-2.330977
61	1	5.955335	3.539647	-2.12527
62	7	6.666533	1.105492	-0.377751
63	1	4.248627	0.937411	-2.242932
64	1	6.279948	2.075055	-3.156916
65	6	5.569285	0.625459	-0.184913
66	6	4.690226	-0.250251	0.508615
67	6	4.430633	-0.046666	1.879318
68	6	4.067636	-1.307673	-0.184317
69	6	3.55924	-0.90054	2.556699
70	1	4.910721	0.777692	2.41109
71	6	3.188589	-2.156695	0.488586
72	1	4.270502	-1.46055	-1.246789
73	6	2.949157	-1.938649	1.847784
74	1	3.354288	-0.752307	3.618646
75	1	2.695388	-2.976151	-0.040721
76	8	7.132255	1.942128	-1.153828
77	17	1.854802	-2.984396	2.677455

Table S10 Regio-isomer 6a’: Energy: -2333.464895 Hartree

Center Number	Atomic Number	Coordinates (Angstroms)		
		X	Y	Z
1	6	-1.367836	-0.417293	-0.736076
2	6	-2.646592	0.105592	-0.472085
3	6	-3.529918	-1.031399	-0.44714

4	7	-1.523636	-1.803792	-0.868766
5	7	-2.812549	-2.178091	-0.678065
6	6	-0.50393	-2.790422	-1.147531
7	6	-0.435603	-3.949214	-0.356222
8	6	0.386602	-2.579938	-2.2126
9	6	0.552012	-4.897269	-0.632999
10	1	-1.15003	-4.107762	0.453797
11	6	1.36613	-3.540585	-2.47131
12	1	0.313141	-1.675262	-2.828235
13	6	1.45209	-4.694762	-1.684783
14	1	0.618077	-5.799471	-0.024268
15	1	2.061247	-3.391037	-3.297348
16	1	2.218711	-5.439988	-1.894544
17	6	-4.978379	-1.058444	-0.230178
18	6	-5.795288	-0.072757	-0.809742
19	6	-5.556377	-2.075727	0.547999
20	6	-7.175766	-0.102381	-0.601576
21	1	-5.34929	0.712582	-1.420946
22	6	-6.938438	-2.097296	0.750991
23	1	-4.924524	-2.849893	0.983457
24	6	-7.749181	-1.111643	0.179368
25	1	-7.806768	0.662289	-1.051412
26	1	-7.384215	-2.886576	1.353801
27	1	-8.825291	-1.131346	0.33896
28	6	-2.990057	1.506294	-0.257892
29	6	-3.801213	1.880682	0.828325
30	6	-2.50762	2.494942	-1.131272
31	6	-4.120226	3.221164	1.036703
32	1	-4.183984	1.116395	1.505326
33	6	-2.829815	3.836828	-0.921669
34	1	-1.883266	2.21217	-1.979402
35	6	-3.636771	4.21089	0.163423
36	1	-4.747166	3.502178	1.880616
37	1	-2.450187	4.593379	-1.605306
38	6	-3.982681	5.64508	0.400354
39	1	-5.070206	5.790016	0.467885
40	1	-3.54714	6.005364	1.343742
41	1	-3.619217	6.307323	-0.395756
42	6	-0.078756	0.319156	-0.876762
43	8	0.46024	0.346209	-1.981558
44	6	0.483962	0.965819	0.303271
45	6	1.684251	1.742679	0.233413
46	6	-0.138641	0.81249	1.568688
47	6	2.223372	2.305832	1.431452
48	6	0.389016	1.372893	2.715245
49	1	-1.062186	0.228221	1.635703

50	6	1.583542	2.122571	2.638196
51	1	3.147212	2.880119	1.380524
52	1	-0.103338	1.241129	3.674607
53	1	1.998671	2.557124	3.549693
54	7	2.281056	1.986326	-0.990918
55	1	1.894484	1.527231	-1.836284
56	6	3.605005	2.613899	-1.150604
57	1	3.51133	3.407895	-1.930611
58	1	3.919435	3.130398	-0.210864
59	6	4.682522	1.596377	-1.580475
60	6	5.961356	2.296973	-2.0784
61	1	5.946992	3.394084	-2.038636
62	7	6.519944	1.01159	-0.213423
63	1	4.273056	0.909204	-2.356887
64	1	6.311392	1.948895	-3.060248
65	6	5.242567	0.784362	-0.403471
66	6	4.44579	-0.146658	0.400413
67	6	4.637464	-0.20861	1.792746
68	6	3.500659	-0.984503	-0.217209
69	6	3.881238	-1.090516	2.567568
70	1	5.382301	0.432532	2.268189
71	6	2.744069	-1.87238	0.549575
72	1	3.349891	-0.946206	-1.296181
73	6	2.94331	-1.906604	1.931409
74	1	4.020543	-1.140799	3.647305
75	1	2.009423	-2.527788	0.076828
76	8	7.026019	1.892515	-1.117453
77	17	1.998583	-2.995361	2.882467

6. *Characterization data of all synthesized compounds*

6.1. Spectroscopic data of the allylated pyrazoles 4a-d[4]

a) 5-(2-(allylaminobenzoyl)-1,3-diphenyl-4-(p-tolyl)-1H-pyrazole (4a)

Yield (83 %); m.p.: 138°C; **FT-IR** (υ_{max}, cm^{-1}): 3292 (N-H), 3061 (=CH vinylic), 3026 (C-H aromatic), 2985, 2913 and 2854 (C-H aliphatic), 1616 (C=O), 1568 (C=N, pyrazole ring), 1515, 1495 and 1429 (C=C, aromatic ring); **^{1}H NMR** (300 MHz, $CDCl_3$) (*δ*/ppm): 2.29 (s, 3H, –C**H**$_3$), 3.89-3.93 (m, 2H, –C**H**$_2$–), 5.18-5.26 (m, 2H, =C**H**$_2$ allylic), 5.87-5.99 (m, 1H, –C**H**= allylic), 6.34-6.37 (m, 1H, Ar–**H**), 6.58 (d, 1H, Ar–**H**, *J* = 8.4 Hz), 7.01-7.13 (m, 2H, Ar–**H**), 7.18-7.24 (m, 2H, Ar–**H**), 7.27-7.37 (m, 8H, Ar–**H**), 7.52-7.61 (m, 4H, Ar–**H**), 8.96 (t, 1H, N-**H**, *J* = 5.7 Hz, exchangeable with D_2O); **^{13}C NMR** (75 MHz, $CDCl_3$) (*δ*/ppm): 21.2 (–**C**H$_3$), 44.7 (–**C**H$_2$), 111.6, 114.5, 116.1 (=**C**H$_2$ allylic), 118.1, 121.1, 122.1, 123.6, 124.9, 127.5, 127.8, 128.2,

128.4, 128.6, 129.7, 130.8, 132.6, 133.9, 134.6, 135.9 (–**C**H= allylic), 136.7, 139.8 (**C**–N), 149.8 (**C**=N), 151.6 (**C**–NH), 190.8 (**C**=O).

b) 5-(2-allylaminobenzoyl)-4-(4-methoxyphenyl)-1,3-diphenyl-1H-pyrazole (4b)

Yield (73 %); m.p.: 146-148 °C; **FT-IR** (υ_{max}, cm^{-1}): 3346 (N-H), 3060 (=CH vinylic), 3026 (C-H aromatic), 2985, 2954 and 2835 (C-H aliphatic), 1614 (>C=O), 1567 (C=N, pyrazole ring), 1497, 1424 (C=C, aromatic ring), 1242 (C-O); **^{1}H NMR** (300 MHz, $CDCl_3$) (δ/ppm): 3.77 (s, 3H, –OC**H**$_3$), 3.89-3.93 (m, 2H, –C**H**$_2$–), 5.17-5.26 (m, 2H, =C**H**$_2$ allylic), 5.87-5.99 (m, 1H, –C**H**= allylic), 6.31-6.37 (m, 1H, Ar–**H**), 6.59 (d, 1H, Ar–**H**, J = 8.4 Hz), 6.74-6.78 (m, 2H, Ar–**H**), 7.13-7.38 (m, 10H, Ar–**H**), 7.52-7.61 (m, 4H, Ar–**H**), 8.96 (t, 1H, N-**H**, J = 5.7 Hz, exchangeable with D_2O); **^{13}C NMR** (75 MHz, $CDCl_3$) (δ/ppm): 44.7 (–**C**H$_2$–), 55.1 (–O**C**H$_3$), 111.7, 113.8, 114.5, 116.1 (=**C**H$_2$ allylic), 117.9, 121.1, 121.8, 123.5, 124.0, 127.5, 127.8, 128.2, 128.4, 128.6, 129.1, 131.0, 132.6, 133.8, 134.6, 135.9 (–**C**H= allylic), 139.8, 139.8 (**C**–N), 149.8 (**C**=N–),151.6 (**C**–NH–),158.6 (**C**–OCH$_3$), 190.8 (**C**=O); ESI-QTOF-MS (m/z): mass calculated for $[C_{32}H_{27}N_3O_2+H]^+$: 486.21761, found: 486.21814.

c) 5-(2-allylaminobenzoyl)-4-(4-chlorophenyl)-1,3-diphenyl-1H-pyrazole (4d)

Yield (69 %); m.p.: 130-132 °C; **FT-IR** (υ_{max}, cm^{-1}): 3352 (N-H), 3081 (=CH vinylic), 3062 and 3015 (C-H aromatic), 2982, 2983 and 2839 (C-H aliphatic), 1613 (C=O), 1566 (C=N, pyrazole ring), 1496 and 1421 (C=C, aromatic ring), 726 (C-Cl); **^{1}H NMR** (300 MHz, $CDCl_3$) (δ/ppm): 3.90-3.95 (m, 2H, –C**H**$_2$–), 5.19-5.27 (m, 2H, =C**H**$_2$ allylic), 5.87-6.00 (m, 1H, –C**H**= allylic), 6.31-6.36 (m, 1H, Ar–**H**), 6.61 (d, 1H, Ar–**H**, J = 8.7 Hz), 7.14-7.39 (m, 12H, Ar–**H**), 7.52-7.57 (m, 4H, Ar–**H**), 8.96 (t, 1H, N-**H**, J = 5.7 Hz, exchangeable with D_2O); **^{13}C NMR** (75 MHz, $CDCl_3$) (δ/ppm): 44.7 (–**C**H$_2$–), 111.8, 114.6, 116.2 (=**C**H$_2$ allylic), 117.7, 120.8, 123.6, 127.7, 128.1, 128.4, 128.6, 129.1, 130.4, 131.1, 132.2, 133.1, 133.7, 134.4, 136.2 (–**C**H= allylic), 139.7, 140.0 (**C**–N), 149.8 (**C**=N–),151.7 (**C**–NH–), 190.3 (**C**=O); ESI-QTOF-MS (m/z): mass calculated for $[C_{31}H_{24}ClN_3O+ H]^+$: 490.16939, found: 490.16973.

6.2. Spectroscopic data of the synthesized hybrids 6a-m

a) 5-(2-(3-(4-chlorophenyl)-4,5-dihydroisoxazol-5-yl)methylamino)benzoyl)-1,3-diphenyl-4-(p-tolyl)-1H-pyrazole (6a)

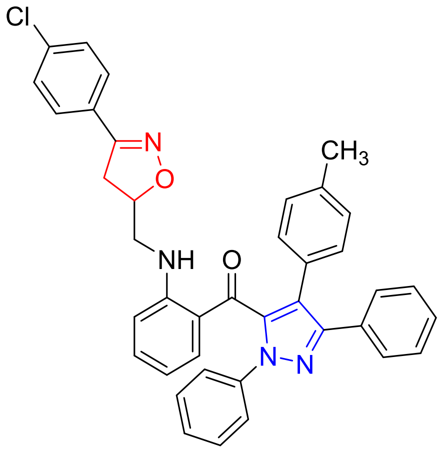

6a

Yield (73 %); m.p.: 164-166 °C; **FT-IR** (υ_{max}, cm^{-1}): 3290 (N-**H**), 3066 (Ar-**H**), 2929, 2854 (C-**H** aliphatic), 1622 (C=O), 1595, 1573 (2 C=N), 1519, 1498, 1458, 1427 (C=C), 1222 (C-O); **^{1}H NMR** (300 MHz, $CDCl_3$) (δ/ppm): δ_H 2.28 (s, 3H, C**H**$_3$), 3.15 (dd, 1H, J = 16.6, 7.3 Hz, C**H**$_{2isoxazoline}$), 3.42 (dd, 1H, J = 16.6, 10.4 Hz, C**H**$_{2isoxazoline}$), 3.47-3.62 (m, 2H, N–C**H**$_2$), 4.97-5.07 (m, 1H, C**H**$_{isoxazoline}$), 6.38 (t, 1H, J = 7.5 Hz, Ar–**H**), 6.71-6.75 (m, 3H, Ar–**H**), 7.09-7.13 (m, 2H, Ar–**H**), 7.22-7.35 (m, 8H, Ar–**H**), 7.37-7.42 (m, 2H, Ar–**H**), 7.49-7.52 (m, 2H, Ar–**H**), 7.56-7.61 (m, 4H, Ar–**H**), 9.01 (t, 1H, N**H**, J = 5.9 Hz, exchangeable with D_2O); **^{13}C NMR** (75 MHz, $CDCl_3$) (δ/ppm): δ_C 21.22 (**C**H$_3$), 38.12 (**C**H$_{2isoxazoline}$), 45.66 (N–**C**H$_2$), 79.53 (**C**H$_{isoxazoline}$), 111.34, 115.28, 118.46, 122.31, 123.62, 127.59, 127.84, 127.91, 128.00, 128.27, 128.45, 128.67, 129.05, 129.08, 129.75, 132.61, 134.80, 136.13, 136.25, 136.79, 139.66, 139.84, 149.92 (**C**=N$_{pyrazole}$), 151.45 (**C**–NH$_2$), 155.54 (**C**=N$_{isoxazoline}$), 190.97 (**C**=O$_{cétone}$); **ESI-QTOF-MS (m/z):** mass calculated for $[C_{39}H_{31}ClN_4O_2+H]^+$ 623.22084, found 623.22079.

b) 5-(2-(3-(4-chlorophenyl)-4,5-dihydroisoxazol-5-yl)methylamino)benzoyl)-4-(4-methoxyphenyl)-1,3-diphenyl-1H-pyrazole (6b)

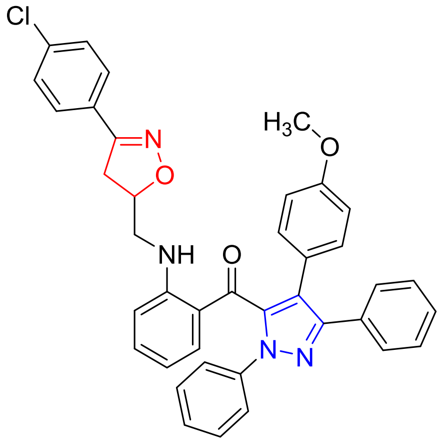

6b

Yield (67 %); m.p.: 144-146 °C; **FT-IR** (υ_{max}, cm^{-1}): 3301 (N-**H**), 3064 (Ar-**H**), 2929, 2866 (C-**H** aliphatic), 1618 (C=O), 1595, 1571 (2 C=N), 1514, 1500, 1454, 1434 (C=C), 1222 (C-O); **^{1}H NMR** (300 MHz, $CDCl_3$) (δ/ppm): δ_H 3.15 (dd, 1H, J = 16.5, 7.2 Hz, C**H**$_{2isoxazoline}$), 3.42 (dd, 1H, J = 16.6, 10.5 Hz, C**H**$_{2isoxazoline}$), 3.48-3.62 (m, 2H, N–C**H**$_2$), 3.75 (s, 3H, OC**H**$_3$), 4.98-5.07 (m, 1H, C**H**$_{isoxazoline}$), 6.38 (t, 1H, J = 7.5 Hz, Ar–**H**), 6.71-6.75 (m, 3H, Ar–**H**), 7.09-7.13 (m, 2H, Ar–**H**), 7.22-7.35 (m, 8H, Ar–**H**), 7.37-7.42 (m, 2H, Ar–**H**), 7.49-7.52 (m, 2H, Ar–**H**), 7.56-7.61 (m, 4H, Ar–**H**), 8.99 (t, 1H, N**H**, J = 6 Hz, exchangeable with D_2O); **^{13}C NMR** (75 MHz, $CDCl_3$) (δ/ppm): δ_C 38.15 (**C**H$_{2isoxazoline}$), 45.70 (N–**C**H$_2$), 55.12 (O**C**H$_3$) 79.49 (**C**H$_{isoxazoline}$), 111.35, 113.80, 115.28, 118.42, 122.04, 123.59, 123.98, 127.57, 127.83, 127.89, 127.99, 128.28, 128.40, 129.08, 131.07, 132.61, 134.80, 136.15, 136.25, 139.61, 139.85, 149.87 (**C**=N$_{pyrazole}$), 151.44 (**C**–NH$_2$), 155.54 (**C**=N$_{isoxazoline}$), 158.73 (**C**–OCH$_3$), 190.98 (**C**=O$_{cétone}$); **ESI-QTOF-MS (m/z):** mass calculated for $[C_{39}H_{31}ClN_4O_3+H]^+$ 639.21677, found 639.21682.

c) 4-(4-bromophenyl)-5-(2-(3-(4-chlorophenyl)-4,5-dihydroisoxazol-5-yl)methylamino)benzoyl)-1,3-diphenyl-1H-pyrazole (6c)

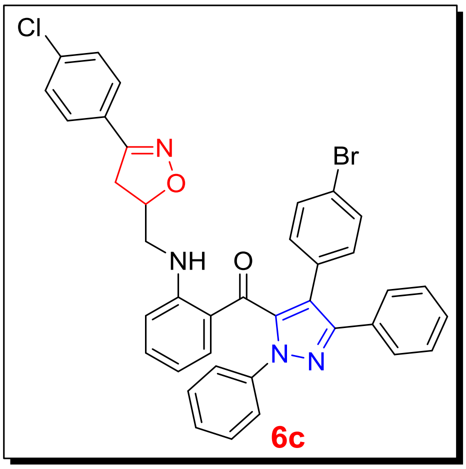

Yield (81 %); m.p.: 152-154 °C; **FT-IR** (υ_{max}, cm^{-1}): 3300 (N-**H**), 3068 (Ar-**H**), 2927, 2869 (C-**H** aliphatic), 1620 (C=O), 1596, 1571 (2 C=N), 1519, 1496, 1458, 1427 (C=C), 1220 (C-O); **^{1}H NMR** (300 MHz, $CDCl_3$) (δ/ppm): δ_H 3.15 (dd, 1H, J = 16.8, 7.5 Hz, C**H**$_{2isoxazoline}$), 3.45 (dd, 1H, J = 16.8, 10.8 Hz, C**H**$_{2isoxazoline}$), 3.49-3.60 (m, 2H, N–C**H**$_2$), 4.98-5.08 (m, 1H, C**H**$_{isoxazoline}$), 6.39 (td, 1H, J = 7.2, 0.9 Hz, Ar–**H**), 6.73 (d, 1H, J = 8.4 Hz, Ar–**H**), 7.06 (t, 2H, J = 1.8 Hz, Ar–**H**), 7.24-7.33 (m, 10H, Ar–**H**), 7.34-7.35 (m, 2H, Ar–**H**), 7.40-7.56 (m, 4H, Ar–**H**), 7.61-7.64 (m, 2H, Ar–**H**), 9.01 (t, 1H, N**H**, J = 6 Hz, exchangeable with D_2O); **^{13}C NMR** (75 MHz, $CDCl_3$) (δ/ppm): δ_C 38.10 (**C**H$_{2isoxazoline}$), 45.72 (N–**C**H$_2$), 79.46 (**C**H$_{isoxazoline}$), 111.52, 115.36, 118.20, 120.97, 121.42, 123.63, 127.81, 127.99, 128.17, 128.43, 128.47, 129.11, 129.15, 130.84, 131.50, 132.17, 134.54, 136.29, 136.40, 139.66, 139.79, 149.87

(**C**=N$_{pyrazole}$), 151.56 (**C**–NH$_2$), 155.54 (**C**=N$_{isoxazoline}$), 190.45 (**C**=O$_{cétone}$); **ESI-QTOF-MS (m/z):** mass calculated for [$C_{38}H_{28}BrClN_4O_2$+H]$^+$ 687.11650, found 687.11621.

d) 4-(4-chlorophenyl)-5-(2-(3-(4-chlorophenyl)-4,5-dihydroisoxazol-5-yl)methylamino)benzoyl)-1,3-diphenyl-1H-pyrazole (6d)

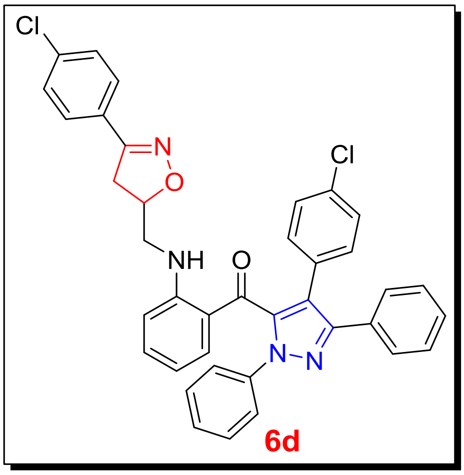

Yield (74 %); m.p.: 166-168 °C; **FT-IR** (υ_{max}, cm^{-1}): 3300 (N-**H**), 3062 (Ar-**H**), 2920, 2850 (C-**H** aliphatic), 1616 (C=O), 1595, 1569 (2 C=N), 1519, 1496, 1427 (C=C), 1217 (C-O); **^{1}H NMR** (300 MHz, CDCl$_3$) (δ/ppm): δ_H 3.16 (dd, 1H, J = 16.5, 6.9 Hz, C**H**$_{2isoxazoline}$), 3.46 (dd, 1H, J = 16.5, 10.5 Hz, C**H**$_{2isoxazoline}$), 3.51-3.63 (m, 2H, N–C**H**$_2$), 5.00-5.09 (m, 1H, C**H**$_{isoxazoline}$), 6.38 (td, 1H, J = 7.2, 0.9 Hz, Ar–**H**), 7.13 (d, 1H, J = 2.7 Hz, Ar–**H**), 7.27-7.35 (m, 4H, Ar–**H**), 7.40-7.54 (m, 16H, Ar–**H**), 7.59 (td, 2H, J = 6.6, 2.1 Hz, Ar–**H**), 9.01 (t, 1H, N**H**, J = 6.1 Hz, exchangeable with D$_2$O); **^{13}C NMR** (75 MHz, CDCl$_3$) (δ/ppm): δ_C 38.12 (**C**H$_{2isoxazoline}$), 45.07 (N–**C**H$_2$), 79.43 (**C**H$_{isoxazoline}$), 111.48, 115.35, 118.14, 120.99, 127.75, 127.81, 127.99, 128.15, 128.43, 128.46, 128.58, 129.11, 129.16, 130.33, 131.16, 132.15, 133.15, 134.58, 136.31, 136.40, 139.63, 139.78, 149.90 (**C**=N$_{pyrazole}$), 151.55 (**C**–NH$_2$), 155.54 (**C**=N$_{isoxazoline}$), 190.46 (**C**=O$_{cétone}$); **ESI-QTOF-MS (m/z):** mass calculated for [$C_{38}H_{28}Cl_2N_4O_2$+H]$^+$ 643.16666, found 643.16649.

e) 4-(4-bromophenyl)-5-(2-(3-(4-nitrophenyl)-4,5-dihydroisoxazol-5-yl)methylamino)benzoyl)-1,3-diphenyl-1H-pyrazole (6e)

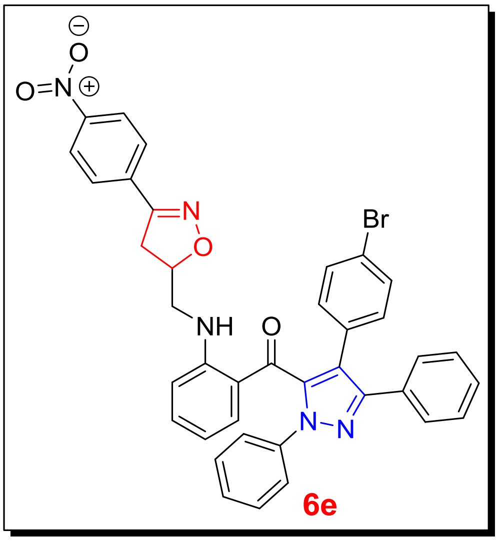

Yield (83 %); m.p.: 184-186 °C; **FT-IR** (υ_{max}, cm^{-1}): 3315 (N-**H**), 3084 (Ar-**H**), 2918, 2856 (C-**H** aliphatic), 1614 (C=O), 1596, 1573 (2 C=N), 1519, 1498, 1433 (C=C), 1215 (C-O); **^{1}H NMR** (300 MHz, $CDCl_3$) (δ/ppm): δ_H 3.21 (dd, 1H, J = 16.8, 7.5 Hz, C**H**$_{2isoxazoline}$), 3.51 (dd, 1H, J = 16.5, 10.5 Hz, C**H**$_{2isoxazoline}$), 3.55-3.65 (m, 2H, N–C**H**$_2$), 5.07-5.17 (m, 1H, C**H**$_{isoxazoline}$), **6**.40 (td, 1H, J = 7.8, 0.9 Hz, Ar–**H**), 6.74 (d, 1H, J = 8.4 Hz, Ar–**H**), 7.05 (dt, 2H, J = 9, 2.1 Hz, Ar–**H**), 7.25-7.36 (m, 10H, Ar–**H**), 7.47-7.55 (m, 4H, Ar–**H**), 7.84 (dt, 2H, J = 9.3, 2.4 Hz, Ar–**H**), 8.28 (dt, 2H, J = 9, 2.1 Hz, Ar–**H**), 9.00 (t, 1H, N**H**, J = 6 Hz, exchangeable with D_2O); **^{13}C NMR** (75 MHz, $CDCl_3$) (δ/ppm): δ_C 37.64 (**C**H$_{2isoxazoline}$), 45.69 (N–**C**H$_2$), 80.30 (**C**H$_{isoxazoline}$), 111.46, 115.54, 118.27, 121.02, 121.42, 123.62, 124.08, 127.47, 127.80, 128.20, 128.45, 129.15, 130.82, 131.49, 132.09, 134.61, 135.31, 136.45, 139.64, 139.70, 148.59 (**C**–NO_2), 149.89 (**C**=N$_{pyrazole}$), 151.45 (**C**–NH_2), 155.00 (**C**=N$_{isoxazoline}$), 190.55 (**C**=O$_{cétone}$); **ESI-QTOF-MS (m/z):** mass calculated for $[C_{38}H_{28}BrN_5O_4+H]^+$ 698.14172, found 698.14121.

f) 5-(2-(3-(4-nitrophenyl)-4,5-dihydroisoxazol-5-yl)methylamino)benzoyl)-1,3-diphenyl--4-(p-tolyl)-1H-pyrazole (6f)

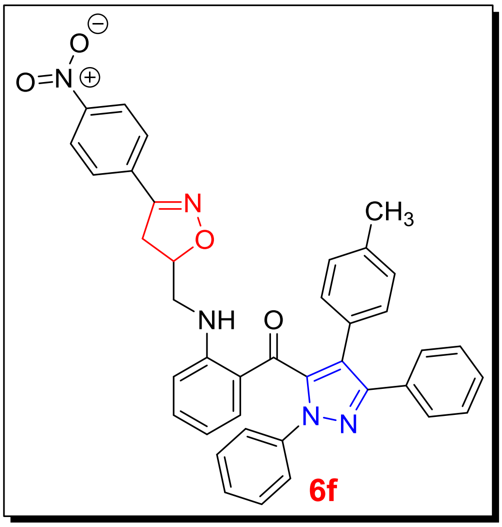

Yield (72 %); m.p.: 180-182 °C; **FT-IR** (υ_{max}, cm^{-1}): 3315 (N-**H**), 3085 (Ar-**H**), 2926, 2850 (C-**H** aliphatic), 1614 (C=O), 1598, 1573 (2 C=N), 1517, 1498, 1433 (C=C), 1215 (C-O); **^{1}H NMR** (300 MHz, $CDCl_3$+ D2O) (δ/ppm): δ_H 2.29 (s, 3H, C**H**$_3$), 3.22 (dd, 1H, J = 16.8, 7.5 Hz, C**H**$_{2isoxazoline}$), 3.45-3.64 (m, 3H, 2H of N–C**H**$_2$ and 1H of C**H**$_{2isoxazoline}$), 5.06-5.16 (m, 1H, C**H**$_{isoxazoline}$), **6**.40 (t, 1H, J = …Hz, Ar–**H**), 6.73 (d, 1H, J = 8.4 Hz, Ar–**H**), 6.97 (d, 2H, J = 7.8 Hz, Ar–**H**), 7.07 (dd, 2H, J = 6.3, 1.8 Hz, Ar–**H**), 7.23-7.39 (m, 8H, Ar–**H**), 7.47-7.51 (m, 2H, Ar–**H**), 7.55-7.58 (m, 2H, Ar–**H**), 7.84 (d, 2H, J = 9 Hz, Ar–**H**), 8.28 (d, 2H, J = 9 Hz, Ar–**H**); **^{13}C NMR** (75 MHz, $CDCl_3$+ D2O) (δ/ppm): δ_C 21.2 (**C**H$_3$), 37.68 (**C**H$_{2isoxazoline}$), 45.75 (N–**C**H$_2$), 80.31 (**C**H$_{isoxazoline}$), 111.41, 115.60, 118.57, 122.37, 123.66, 124.06, 127.49, 127.65, 127.99, 128.29, 128.45, 128.57, 129.05, 129.09, 129.74, 132.42, 134.85, 135.35, 136.20, 136.85, 139.57, 139.72, 148.56 (**C**–NO$_2$), 149.91 (**C**=N$_{pyrazole}$), 151.19 (**C**–NH$_2$), 155.00 (**C**=N$_{isoxazoline}$), 190.97 (**C**=O$_{cétone}$); **ESI-QTOF-MS (m/z):** mass calculated for $[C_{39}H_{31}N_5O_4+H]^+$ 634.24591, found 634.24529.

g) 4-(4-methoxyphenyl)-5-(2-(3-(4-nitrophenyl)-4,5-dihydroisoxazol-5-yl)methylamino)benzoyl)-1,3-diphenyl-1H-pyrazole (6g)

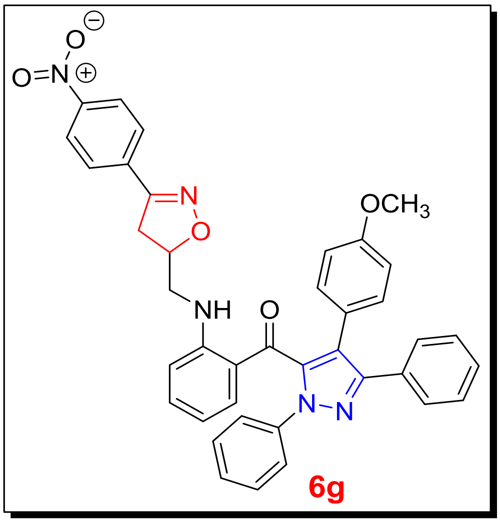

Yield (58 %); m.p.: 170-172 °C; **FT-IR** (υ_{max}, cm^{-1}): 3300 (N-**H**), 3070 (Ar-**H**), 2927, 2854 (C-**H** aliphatic), 1620 (C=O), 1596, 1571 (2 C=N), 1515, 1502, 1458, 1431 (C=C), 1222 (C-O); **^{1}H NMR** (300 MHz, $CDCl_3$+ D2O) (*δ*/ppm): δ_H 3.22 (dd, 1H, *J* = 16.8, 7.5 Hz, C**H**$_{2isoxazoline}$), 3.44-3.63 (m, 3H, 2H of N–CH_2 and 1H of C**H**$_{2isoxazoline}$), 3.75 (s, 3H, OC**H**$_3$), 5.06-5.16 (m, 1H, C**H**$_{isoxazoline}$), **6**.41 (t, 1H, *J* = 7.8 Hz, Ar–**H**), 6.69-6.74 (m, 3H, Ar–**H**), 7.10 (d, 2H, *J* = 8.7 Hz, Ar–**H**), 7.24-7.39 (m, 8H, Ar–**H**), 7.48-7.51 (m, 2H, Ar–**H**), 7.55-7.58 (m, 2H, Ar–**H**), 7.84 (d, 2H, *J* = 9 Hz, Ar–**H**), 8.28 (d, 2H, *J* = 9 Hz, Ar–**H**); **^{13}C NMR** (75 MHz, $CDCl_3$+ D2O) (*δ*/ppm): δ_C 37.67 (**C**H$_{2isoxazoline}$), 45.73 (N–**C**H$_2$), 55.12 (s, 3H, O**C**H$_3$), 80.30 (**C**H$_{isoxazoline}$), 111.39, 113.79, 115.56, 118.51, 122.08, 123.61, 123.89, 124.05, 127.48, 127.61, 127.97, 128.31, 128.40, 129.10, 131.05, 132.45, 134.86, 135.33, 136.22, 139.53, 139.75, 148.56 (**C**–NO$_2$), 149.87 (**C**=N$_{pyrazole}$), 151.24 (**C**–NH$_2$), 155.00 (**C**=N$_{isoxazoline}$), 158.73 (**C**–OCH$_3$), 190.00 (**C**=O$_{cétone}$); **ESI-QTOF-MS (m/z):** mass calculated for $[C_{39}H_{31}N_5O_5+H]^+$ 650.24170, found 650.24097.

h) 4-(4-bromophenyl)-5-(2-(3-(4-methoxyphenyl)-4,5-dihydroisoxazol-5-yl)methylamino)benzoyl)-1,3-diphenyl-1H-pyrazole (6h)

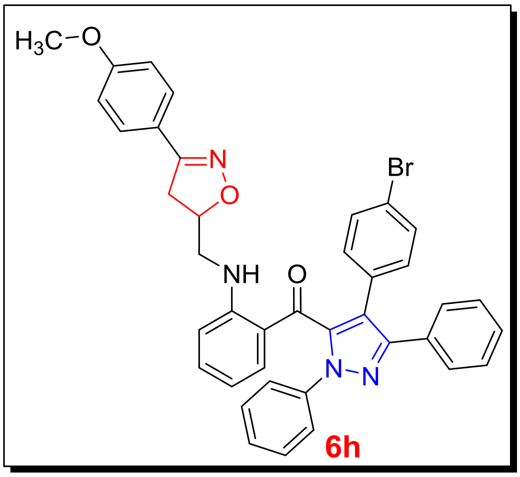

Yield (86 %); m.p.: 136-138 °C; **FT-IR** (υ_{max}, cm^{-1}): 3319 (N-**H**), 3064 (Ar-**H**), 2929, 2842 (C-**H** aliphatic), 1616 (C=O), 1602, 1573 (2 C=N), 1523, 1500, 1431 (C=C), 1215 (C-O); **^{1}H NMR** (300 MHz, $CDCl_3$+ D2O) (δ/ppm): δ_H 3.14 (dd, 1H, J = 16.8, 7.2 Hz, C**H**$_{2isoxazoline}$), 3.43 (dd, 1H, J = 16.5, 10.5 Hz, C**H**$_{2isoxazoline}$), 3.48-3.61 (m, 2H, N–C**H**$_2$), 3.75 (s, 3H, OC**H**$_3$), 4.97-5.07 (m, 1H, C**H**$_{isoxazoline}$), **6**.41 (t, 1H, J = 7.8 Hz, Ar–**H**), 6.69-6.74 (m, 3H, Ar–**H**), 7.10 (d, 2H, J = 8.7 Hz, Ar–**H**), 7.24-7.39 (m, 8H, Ar–**H**), 7.48-7.51 (m, 2H, Ar–**H**), 7.55-7.58 (m, 2H, Ar–**H**), 7.84 (d, 2H, J = 9 Hz, Ar–**H**), 8.28 (d, 2H, J = 9 Hz, Ar–**H**); **^{13}C NMR** (75 MHz, $CDCl_3$) (δ/ppm): δ_C 38.27 (**C**H$_{2isoxazoline}$), 45.77 (N–**C**H$_2$), 56.30 (s, 3H, O**C**H$_3$), 79.17 (**C**H$_{isoxazoline}$), 111.64, 111.98, 115.44, 118.20, 120.95, 122.66, 123.00, 123.67, 126.46, 127.86, 128.17, 128.43, 128.48, 128.64, 129.16, 130.78, 131.49, 131.52, 132.09, 134.53, 136.41, 139.58, 139.77, 149.84 (**C**=N$_{pyrazole}$), 151.45 (**C**–NH$_2$), 155.11 (**C**=N$_{isoxazoline}$), 156.49 (**C**–OCH$_3$), 190.40 (**C**=O$_{cétone}$); **ESI-QTOF-MS (m/z):** mass calculated for $[C_{39}H_{31}BrN_4O_3+H]^+$ 683.1656, found 683.1661.

i) 4-(4-methoxyphenyl)-5-(2-(3-(4-methoxyphenyl)-4,5-dihydroisoxazol-5-yl)methylamino)benzoyl)-1,3-diphenyl-1H-pyrazole (6i)

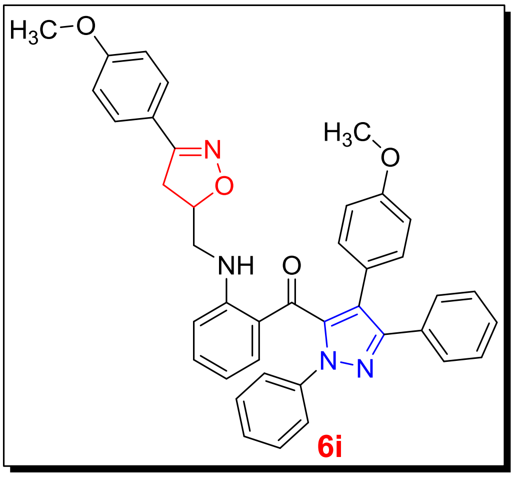

Yield (77 %); m.p.: 172-174 °C; **FT-IR** (υ_{max}, cm^{-1}): 3325 (N-**H**), 3076 (Ar-**H**), 2933, 2835 (C-**H** aliphatic), 1610 (C=O), 1568 (C=N), 1514, 1498, 1456, 1429 (C=C), 1218 (C-O); **^{1}H NMR** (300 MHz, $CDCl_3$+ D2O) (δ/ppm): δ_H 3.16 (dd, 1H, J = 16.5, 6.9 Hz, C**H**$_{2isoxazoline}$), 3.40-3.49 (m, 2H, N–C**H**$_2$), 3.57 (dd, 1H, J = 13.8, 5.7 Hz, C**H**$_{2isoxazoline}$), 3.75 (s, 3H, OC**H**$_3$), 3.86 (s, 3H, OC**H**$_3$), 4.93-5.03 (m, 1H, C**H**$_{isoxazoline}$), **6**.38 (t, 1H, J = 7.5 Hz, Ar–**H**), 6.73 (d, 3H, J = 8.7 Hz, Ar–**H**), 6.95 (d, 2H, J = 8.7 Hz, Ar–**H**), 7.11-7.14 (m, 2H, Ar–**H**), 7.21-7.49 (m, 8H, Ar–**H**), 7.48-7.51 (m, 2H, Ar–**H**), 7.55-7.58 (m, 2H, Ar–**H**), 7.84 (d, 2H, J = 9 Hz, Ar–**H**); **^{13}C NMR** (75 MHz, $CDCl_3$+ D2O) (δ/ppm): δ_C 38.62 (**C**H$_{2isoxazoline}$), 45.83 (N–**C**H$_2$), 55.13 (s, 3H, OCH$_3$), 55.40 (s, 3H, OCH$_3$), 78.86 (**C**H$_{isoxazoline}$), 111.53, 113.82, 114.21, 115.27, 118.39,

121.84, 122.02, 123.62, 123.94, 127.61, 127.91, 128.30, 128.33, 128.42, 129.12, 131.07, 132.58, 134.75, 136.16, 139.66, 139.79, 149.83 (**C**=N$_{pyrazole}$), 151.41 (**C**–NH$_2$), 156.01 (**C**=N$_{isoxazoline}$), 158.72 (**C**–OCH$_3$), 161.21 (**C**–OCH$_3$), 190.87 (**C**=O$_{cétone}$); **ESI-QTOF-MS (m/z):** mass calculated for $[C_{40}H_{34}N_4O_4+H]^+$ 635.26611, found 635.26591.

j) 1,3-diphenyl-4-(p-tolyl)-5-(2-(3-(p-tolyl)-4,5-dihydroisoxazol-5-yl)methylamino)benzoyl)-1H-pyrazole (6j)

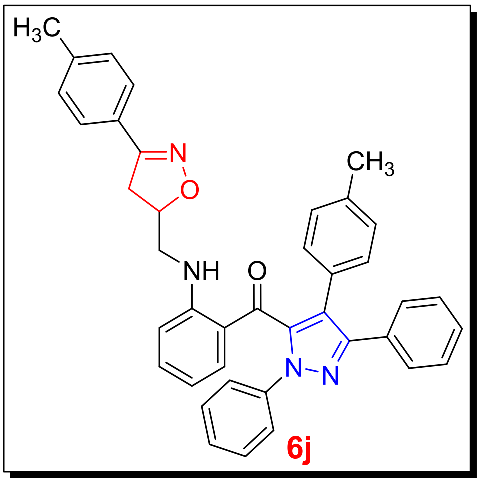

Yield (83 %); m.p.: 150-152 °C; **FT-IR** (υ_{max}, cm^{-1}): 3298 (N-**H**), 3064 (Ar-**H**), 2920, 2856 (C-**H** aliphatic), 1620 (C=O), 1596, 1573 (2 C=N), 1517, 1500, 1456, 1425 (C=C), 1222 (C-O); **^{1}H NMR** (300 MHz, CDCl$_3$+ D2O) (δ/ppm): δ_H 2.28 (s, 3H, C**H**$_3$), 2.41 (s, 3H, C**H**$_3$), 3.17 (dd, 1H, J = 16.5, 7.2 Hz, C**H**$_{2isoxazoline}$), 3.40-3.52 (m, 2H, N–C**H**$_2$), 3.58 (dd, 1H, J = 13.8, 5.7 Hz, C**H**$_{2isoxazoline}$), 4.94-5.04 (m, 1H, C**H**$_{isoxazoline}$), **6**.38 (t, 1H, J = 7.5 Hz, Ar–**H**), 6.73 (d, 3H, J = 8.7 Hz, Ar–**H**), 6.95 (d, 2H, J = 8.7 Hz, Ar–**H**), 7.11-7.14 (m, 2H, Ar–**H**), 7.21-7.49 (m, 8H, Ar–**H**), 7.48-7.51 (m, 2H, Ar–**H**), 7.55-7.58 (m, 2H, Ar–**H**), 7.84 (d, 2H, J = 9 Hz, Ar–**H**); **^{13}C NMR** (75 MHz, CDCl$_3$+ D2O) (δ/ppm): δ_C 21.24 (s, 3H, **C**H$_3$), 21.51 (s, 3H, **C**H$_3$), 38.45 (**C**H$_{2isoxazoline}$), 45.79 (N–**C**H$_2$), 79.00 (**C**H$_{isoxazoline}$), 111.53, 115.31, 118.46, 122.30, 123.66, 126.48, 126.74, 127.64, 127.93, 128.28, 128.47, 128.63, 129.07, 129.11, 129.5, 129.75, 132.57, 134.75, 136.14, 136.8, 139.72, 139.77, 140.57, 149.87 (**C**=N$_{pyrazole}$), 151.39 (**C**–NH$_2$), 156.40 (**C**=N$_{isoxazoline}$), 190.87 (**C**=O$_{cétone}$); **ESI-QTOF-MS (m/z):** mass calculated for $[C_{40}H_{34}N_4O_2+H]^+$ 603.27622, found 603.27545.

k) 5-(2-(3-(4-bromophenyl)-4,5-dihydroisoxazol-5-yl)methylamino)benzoyl)-4-(4-methoxyphenyl)-1,3-diphenyl-1H-pyrazole (6k)

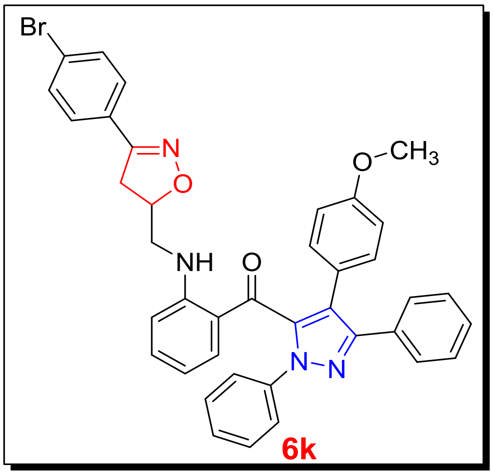

Yield (88 %); m.p.: 152-154 °C; **FT-IR** (υ_{max}, cm^{-1}): 3300 (N-**H**), 3062 (Ar-**H**), 2927, 2852 (C-**H** aliphatic), 1618 (C=O), 1595, 1571 (C=N), 1517, 1500, 1456, 1434 (C=C), 1220 (C-O); **^{1}H NMR** (300 MHz, $CDCl_3$+ D2O) (δ/ppm): δ_H 3.16 (dd, 1H, J = 16.8, 7.2 Hz, C**H**$_{2isoxazoline}$), 3.40-3.52 (m, 2H, N–C**H**$_2$), 3.44 (dd, 1H, J = 13.8, 5.7 Hz, C**H**$_{2isoxazoline}$), 3.75 (s, 3H, OC**H**$_3$), 4.94-5.04 (m, 1H, C**H**$_{isoxazoline}$), **6**.38 (t, 1H, J = 7.5 Hz, Ar–**H**), 6.73 (d, 3H, J = 8.7 Hz, Ar–**H**), 6.95 (d, 2H, J = 8.7 Hz, Ar–**H**), 7.11-7.14 (m, 2H, Ar–**H**), 7.21-7.49 (m, 8H, Ar–**H**), 7.48-7.51 (m, 2H, Ar–**H**), 7.55-7.58 (m, 2H, Ar–**H**), 7.84 (d, 2H, J = 9 Hz, Ar–**H**); **^{13}C NMR** (75 MHz, $CDCl_3$+ D2O) (δ/ppm): δ_C 38.08 (**C**H$_{2isoxazoline}$), 45.71 (N–**C**H$_2$), 55.13 (s, 3H, O**C**H$_3$), 79.51 (**C**H$_{isoxazoline}$), 111.38, 113.80, 115.32, 118.39, 122.04, 123.60, 123.95, 124.58, 127.60, 127.91, 128.21, 128.30, 128.41, 129.10, 131.07, 132.03, 132.57, 134.81, 136.18, 139.59, 139.81, 149.86 (**C**=N$_{pyrazole}$), 151.41 (**C**–NH$_2$), 155.64 (**C**=N$_{isoxazoline}$), 158.71 (**C**–OCH$_3$), 190.96 (**C**=O$_{cétone}$); **ESI-QTOF-MS (m/z):** mass calculated for $[C_{39}H_{31}BrN_4O_3+H]^+$ 683.16608, found 683.16602.

l) 4-(4-methoxyphenyl)-1,3-diphenyl-5-(2-(3-(p-tolyl)-4,5-dihydroisoxazol-5-yl)methylamino)benzoyl)-1H-pyrazole (6l)

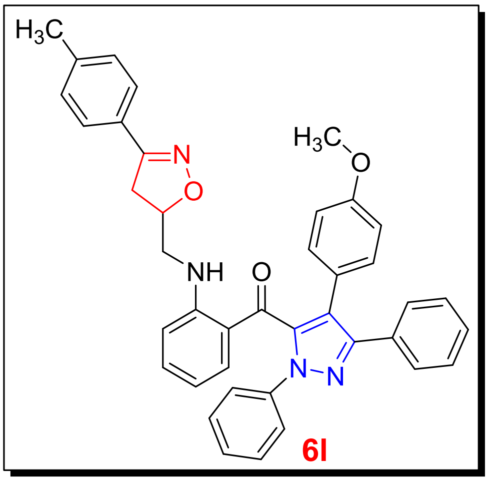

Yield (84 %); m.p.: 162-164 °C; **FT-IR** (υ_{max}, cm^{-1}): 3305 (N-**H**), 3058 (Ar-**H**), 2927, 2854 (C-**H** aliphatic), 1618 (C=O), 1593, 1571 (2 C=N), 1519, 1500, 1454, 1429 (C=C), 1220 (C-O); **^{1}H NMR** (300 MHz, $CDCl_3$+ D2O) (*δ*/ppm): δ_H 2.41 (s, 3H, C**H**$_3$), 3.17 (dd, 1H, *J* = 16.8, 7.2 Hz, C**H**$_{2isoxazoline}$), 3.41-3.58 (m, 3H, 2H of N–C**H**$_2$ and 1H of C**H**$_{2isoxazoline}$), 3.74 (s, 3H, OC**H**$_3$), 4.95-5.04 (m, 1H, C**H**$_{isoxazoline}$), **6**.38 (t, 1H, *J* = 7.5 Hz, Ar–**H**), 6.73 (d, 3H, *J* = 8.7 Hz, Ar–**H**), 6.95 (d, 2H, *J* = 8.7 Hz, Ar–**H**), 7.11-7.14 (m, 2H, Ar–**H**), 7.21-7.49 (m, 8H, Ar–**H**), 7.48-7.51 (m, 2H, Ar–**H**), 7.55-7.58 (m, 2H, Ar–**H**), 7.84 (d, 2H, *J* = 9 Hz, Ar–**H**); **^{13}C NMR** (75 MHz, $CDCl_3$+ D2O) (*δ*/ppm): δ_C 21.50 (s, 3H, **C**H$_3$), 38.48 (**C**H$_{2isoxazoline}$), 45.85 (N–**C**H$_2$), 55.12 (s, 3H, OCH$_3$), 78.55 (**C**H$_{isoxazoline}$), 111.55, 113.82, 115.32, 118.41, 122.03, 123.63, 123.93, 126.47, 126.73, 127.62, 127.92, 128.30, 128.42, 129.11, 129.50, 131.07, 132.56, 134.75, 136.16, 139.66, 139.77, 140.58, 149.82 (**C**=N$_{pyrazole}$), 151.37 (**C**–NH$_2$), 156.40 (**C**=N$_{isoxazoline}$), 158.73 (**C**–OCH$_3$), 190.86 (**C**=O$_{cétone}$); **ESI-QTOF-MS (m/z):** mass calculated for $[C_{40}H_{34}N_4O_3+H]^+$ 619.27105, found 619.27075.

m) 4-(4-chlorophenyl)-5-(2-(3-(2-chlorophenyl)-4,5-dihydroisoxazol-5-yl)methylamino)benzoyl)-1,3-diphenyl-1H-pyrazole (6m)

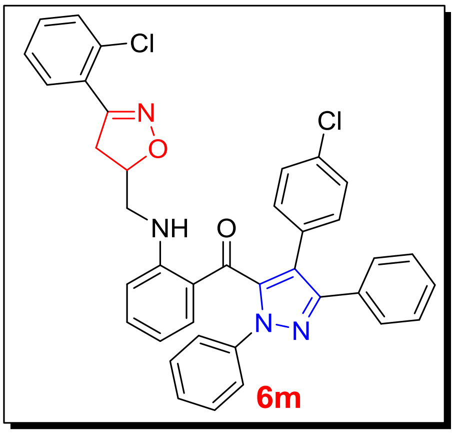

Yield (76 %); m.p.: 180-190 °C; **FT-IR** (υ_{max}, cm^{-1}): 3305 (N-**H**), 3058 (Ar-**H**), 2918, 2854S (C-**H** aliphatic), 1620 (C=O), 1595, 1569 (2 C=N), 1517, 1496, 1452, 1429 (C=C), 1220 (C-O); **^{1}H NMR** (300 MHz, $CDCl_3$+ D2O) (*δ*/ppm): δ_H 3.36 (dd, 1H, *J* = 17.1, 6.9 Hz, C**H**$_{2isoxazoline}$), 3.49-3.59 (m, 2H, N–C**H**$_2$), 3.65 (dd, 1H, *J* = 17.1, 10.5 Hz, C**H**$_{2isoxazoline}$), 5.02-5.11 (m, 1H, C**H**$_{isoxazoline}$), **6**.38 (t, 1H, *J* = 7.5 Hz, Ar–**H**), 6.73 (d, 3H, *J* = 8.7 Hz, Ar–**H**), 6.95 (d, 2H, *J* = 8.7 Hz, Ar–**H**), 7.11-7.14 (m, 2H, Ar–**H**), 7.21-7.49 (m, 8H, Ar–**H**), 7.48-7.51 (m, 2H, Ar–**H**), 7.55-7.58 (m, 2H, Ar–**H**), 7.84 (d, 2H, *J* = 9 Hz, Ar–**H**); **^{13}C NMR** (75 MHz, $CDCl_3$+ D2O) (*δ*/ppm): δ_C 40.68 (**C**H$_{2isoxazoline}$), 45.64 (N–**C**H$_2$), 79.70 (**C**H$_{isoxazoline}$), 111.63, 115.33, 118.22, 120.97, 123.66, 127.12, 127.83, 128.15, 128.60, 128.78, 130.32, 130.57, 130.70, 131.07, 132.17, 132.87, 133.16, 134.53, 136.36, 139.63, 139.85, 149.88 (**C**=N$_{pyrazole}$),

151.53 (**C**–NH_2), 156.49 (**C**=N$_{\text{isoxazoline}}$), 190.39 (**C**=O$_{\text{cétone}}$); **ESI-QTOF-MS (m/z):** mass calculated for $[C_{38}H_{28}Cl_2N_4O_2+H]^+$ 643.16704, found 643.16668.

7. *^{1}H, ^{13}C NMR and HRMS Spectra of hybrid molecules pyrazole-isoxazoline 6a-m*

a) 5-(2-(3-(4-chlorophenyl)-4,5-dihydroisoxazol-5-yl)methylamino)benzoyl)-1,3-diphenyl-4-(p-tolyl)-1H-pyrazole (6a)

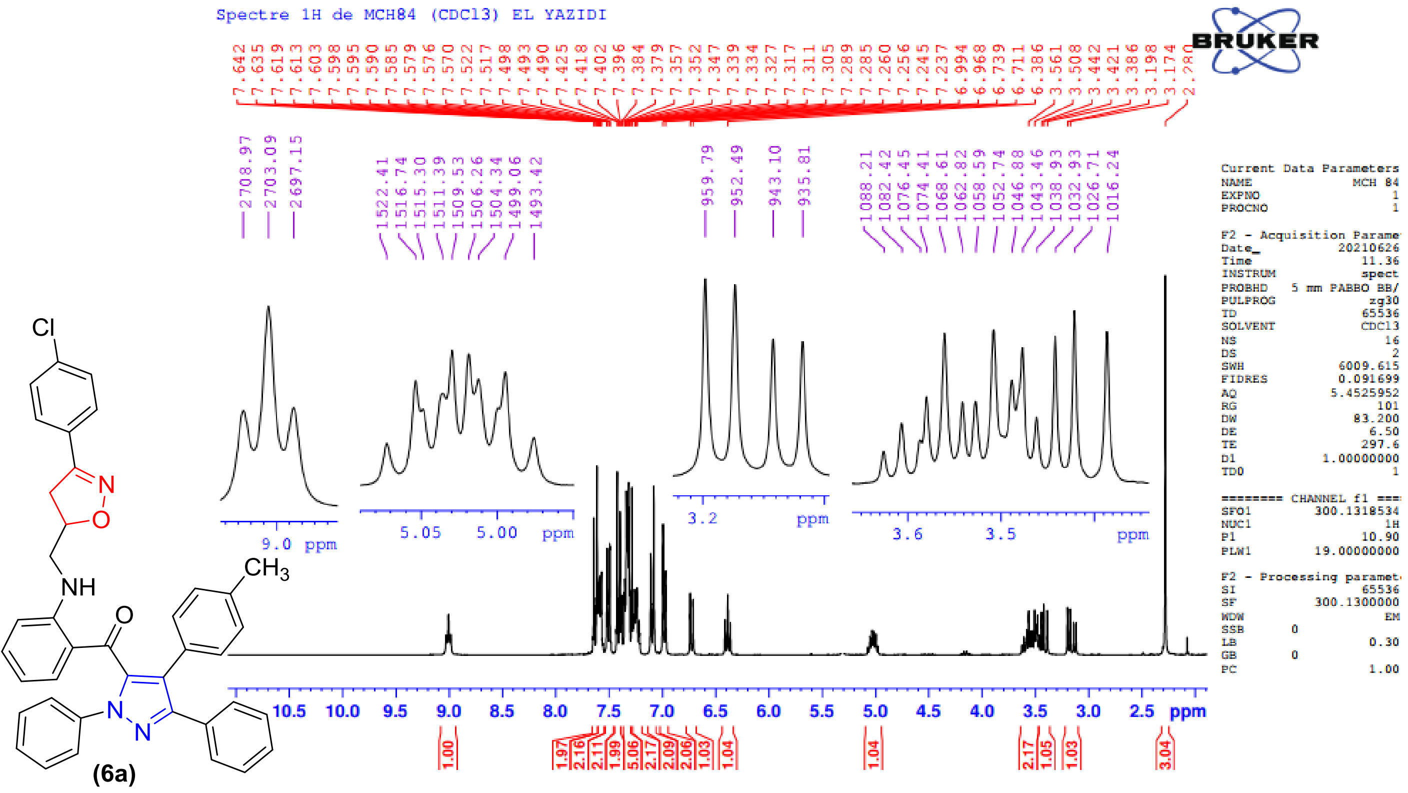

Figure S2. ^{1}H NMR spectrum (300 MHz, $CDCl_3$) of compound (**6a**)

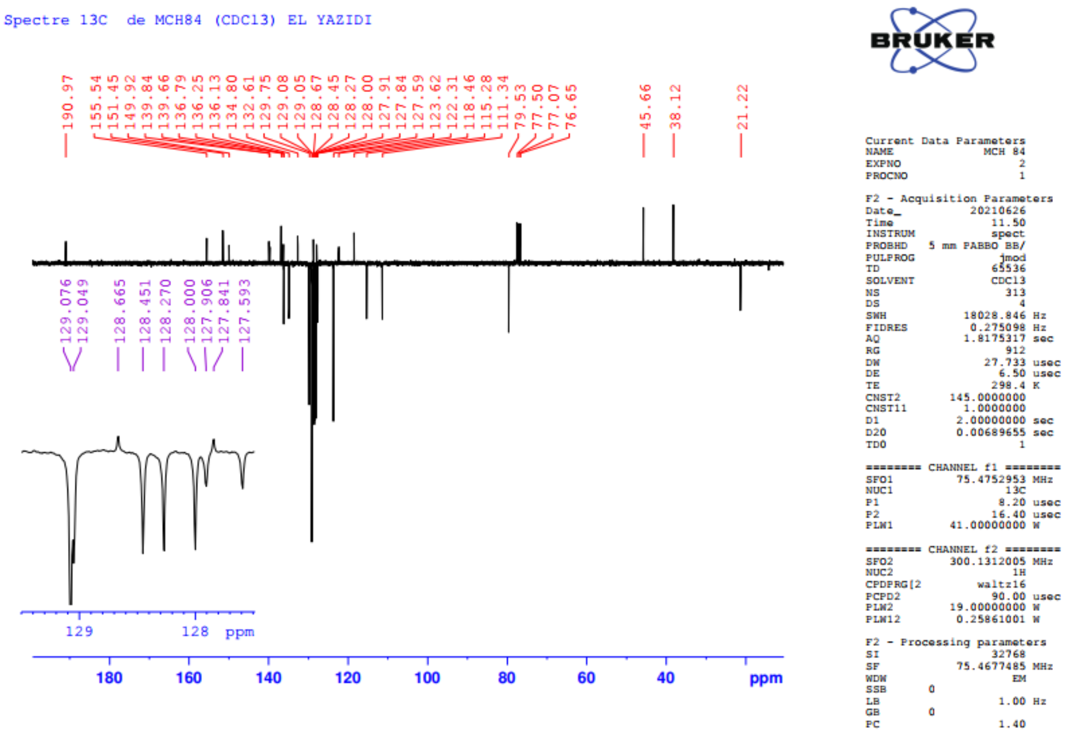

Figure S3. ^{13}C NMR spectrum (75 MHz, $CDCl_3$) of compound (**6a**)

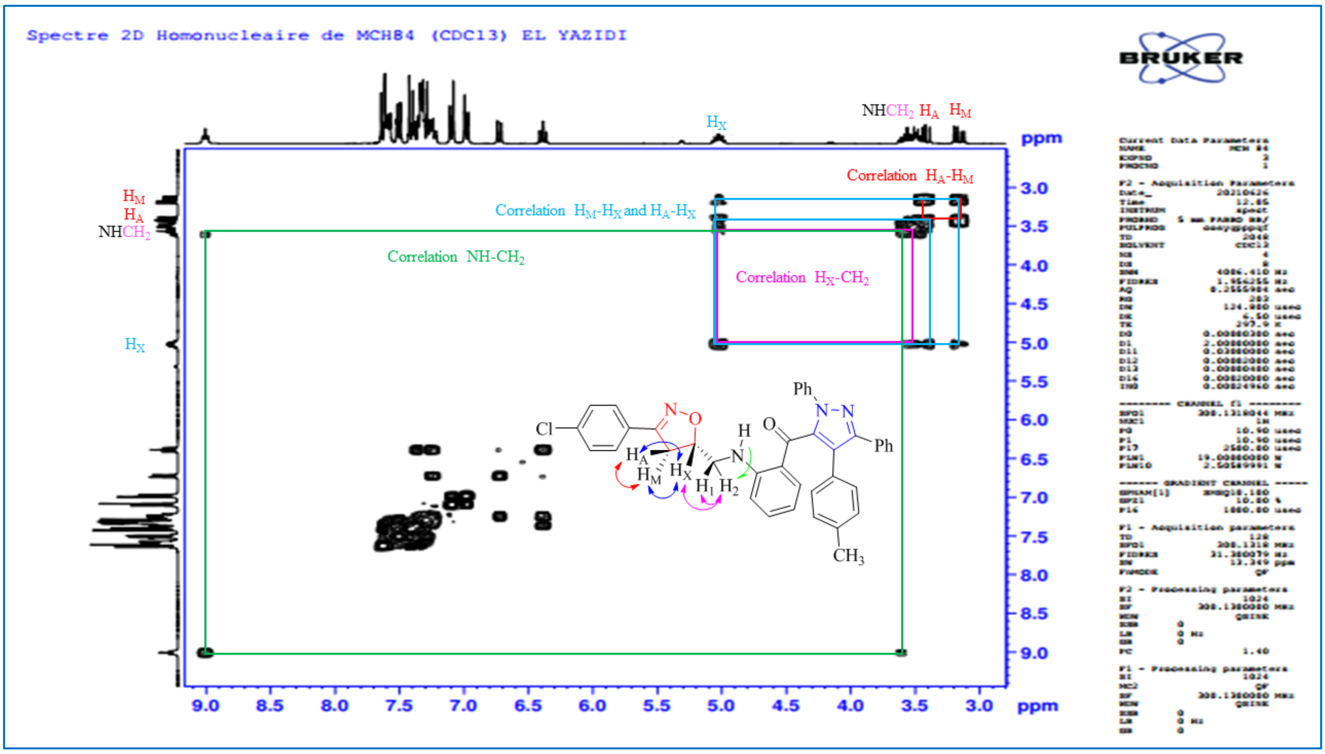

Figure S4. 2D-COSY NMR spectrum of compound **6a** showing the coupling signals for hydrogen atoms.

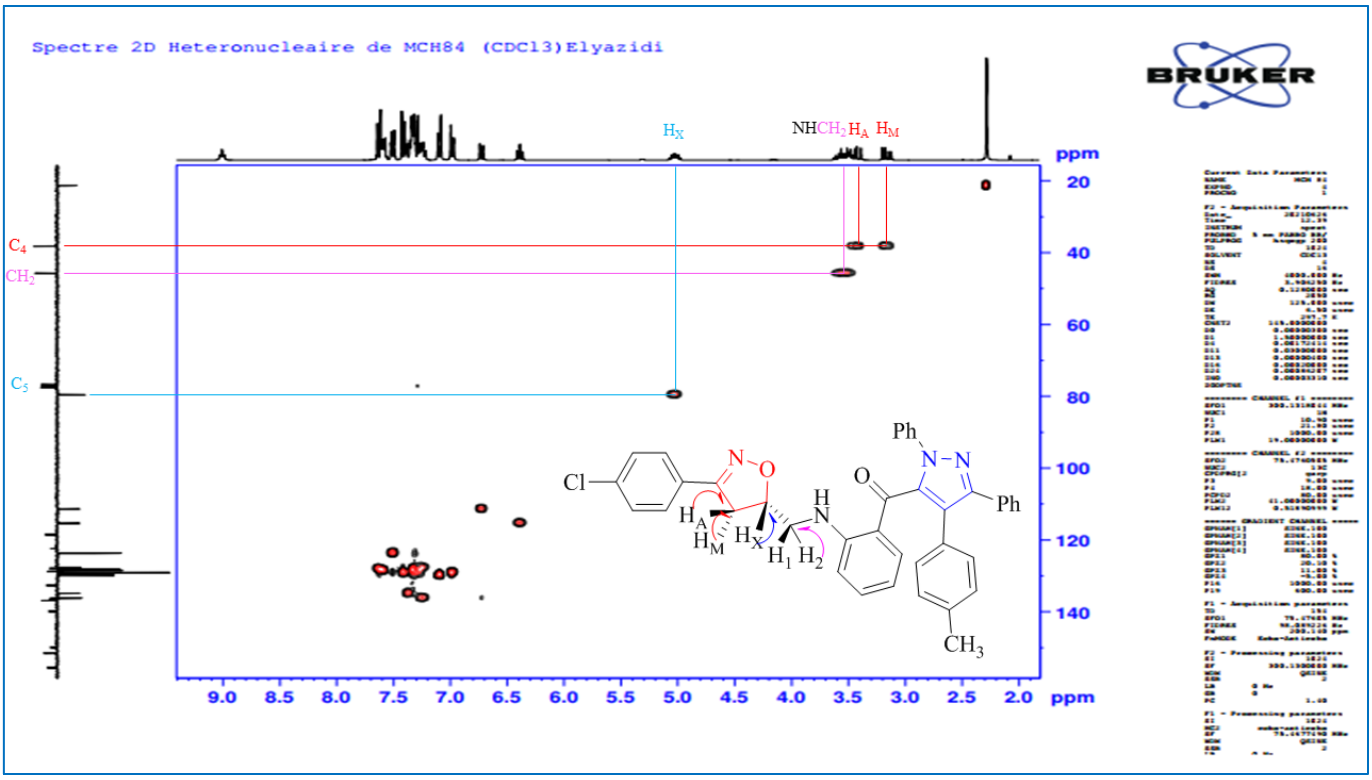

Figure S5. 2D-HSQC NMR spectrum of compound **6a** showing the identification of methine **C**5 and methylene **C**4 carbons of isoxazoline and exocyclic methylene carbon **C**H2.

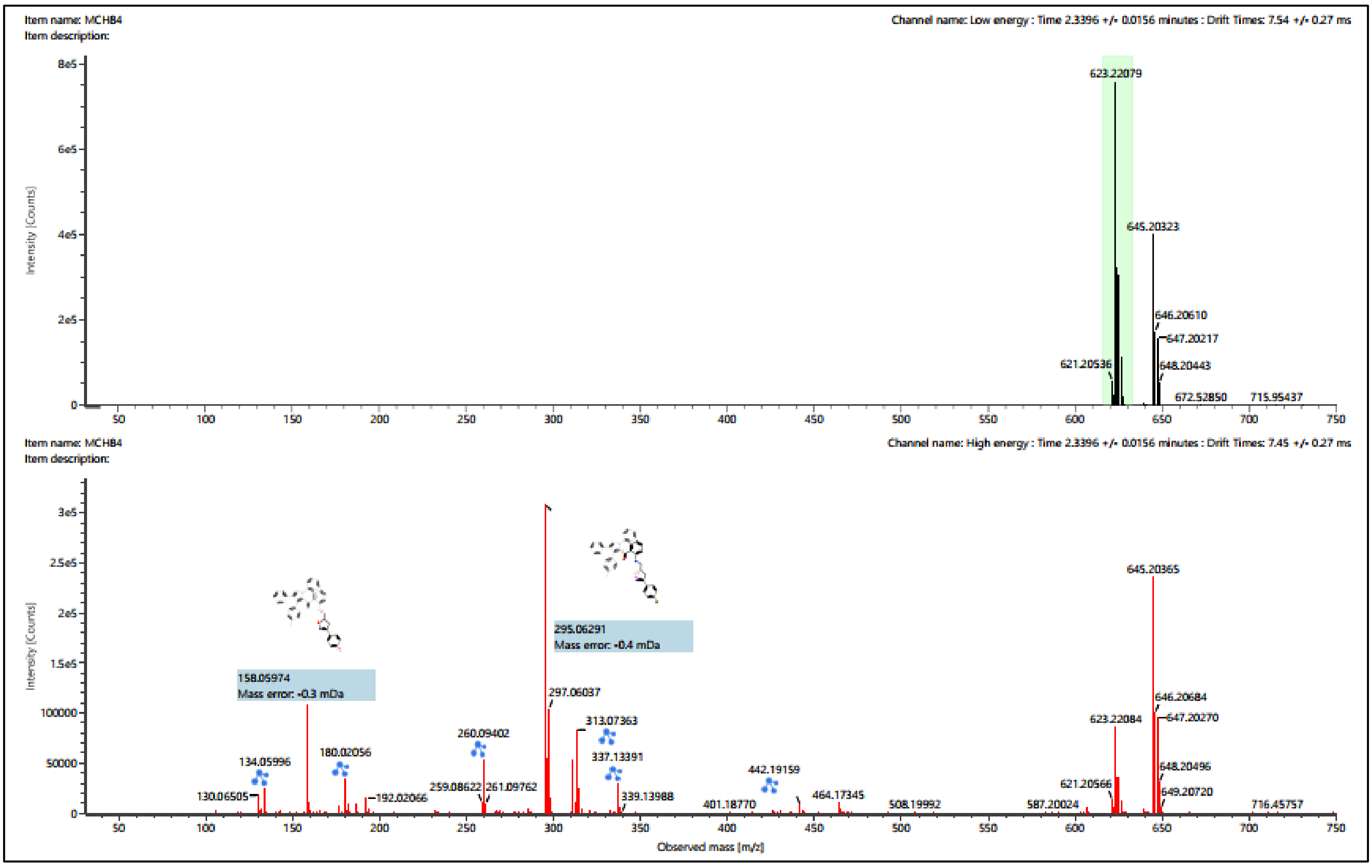

Figure S6. Mass Spectrum of compound (**6a**)

b) 5-(2-(3-(4-chlorophenyl)-4,5-dihydroisoxazol-5-yl)methylamino)benzoyl)-4-(4-methoxyphenyl)-1,3-diphenyl-1H-pyrazole (6b)

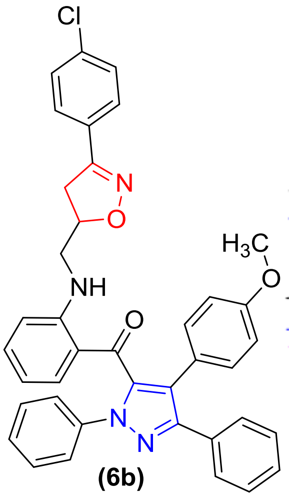

(6b)

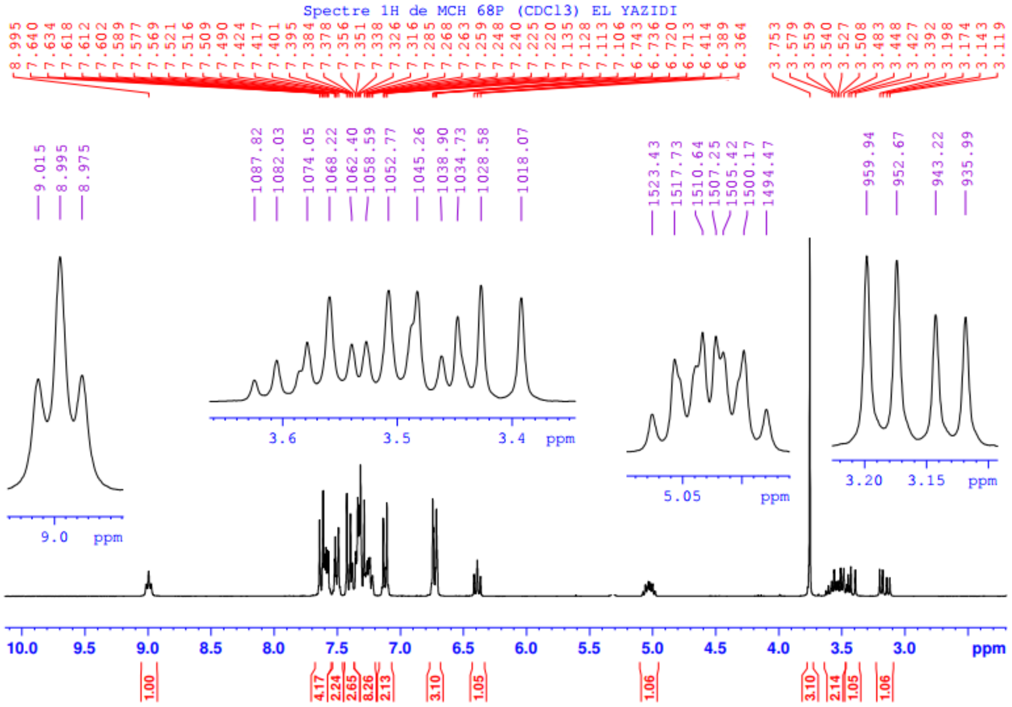

Figure S7. ^{1}H NMR spectrum (300 MHz, $CDCl_3$) of compound (**6b**)

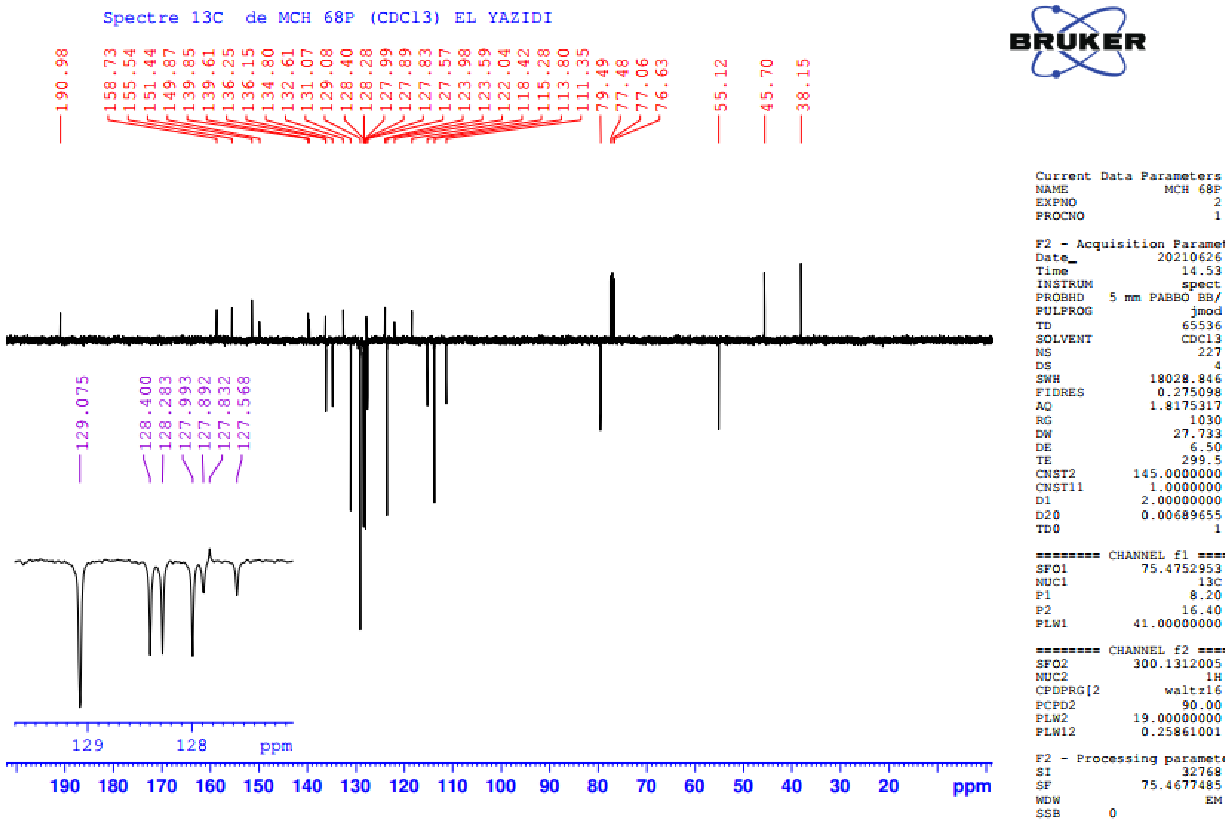

Figure S8. ^{13}C NMR spectrum (75 MHz, $CDCl_3$) of compound (**6b**)

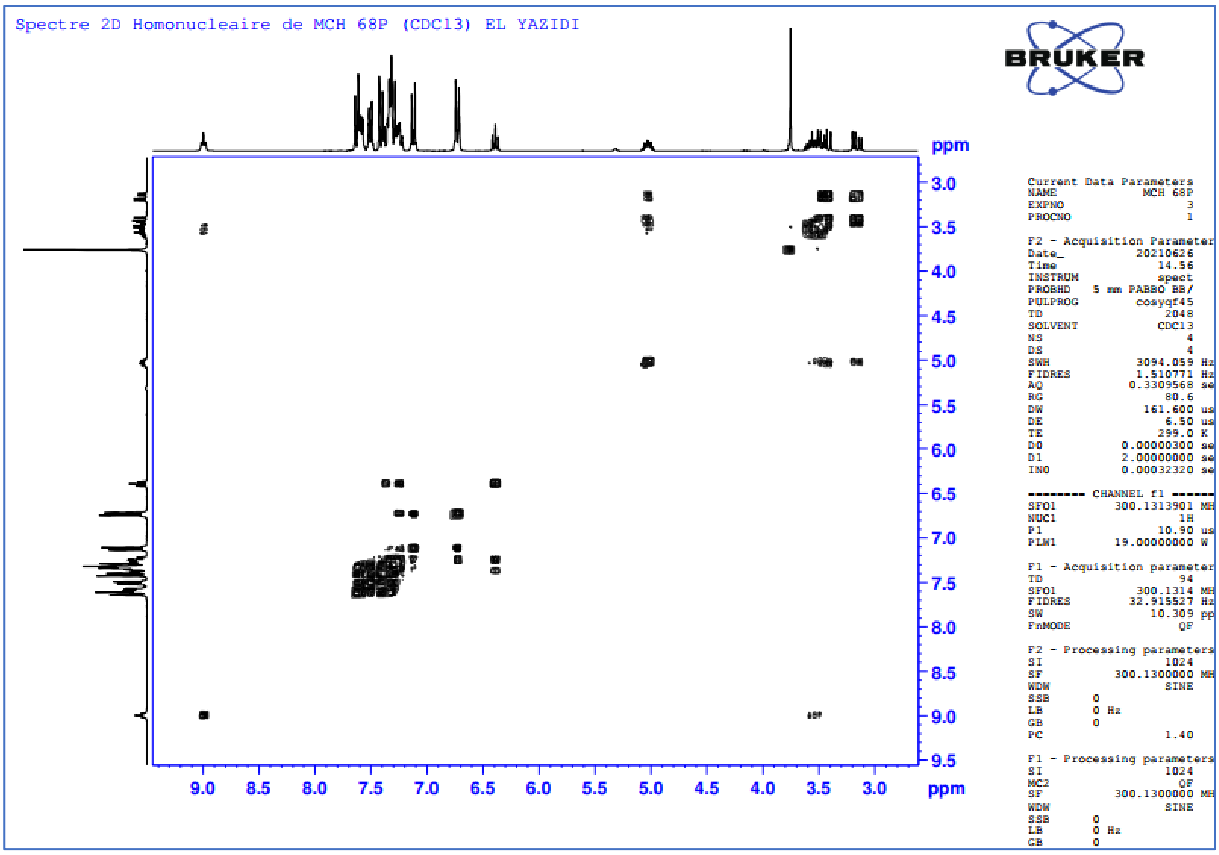

Figure S9. 2D-COSY NMR spectrum of compound **6b**

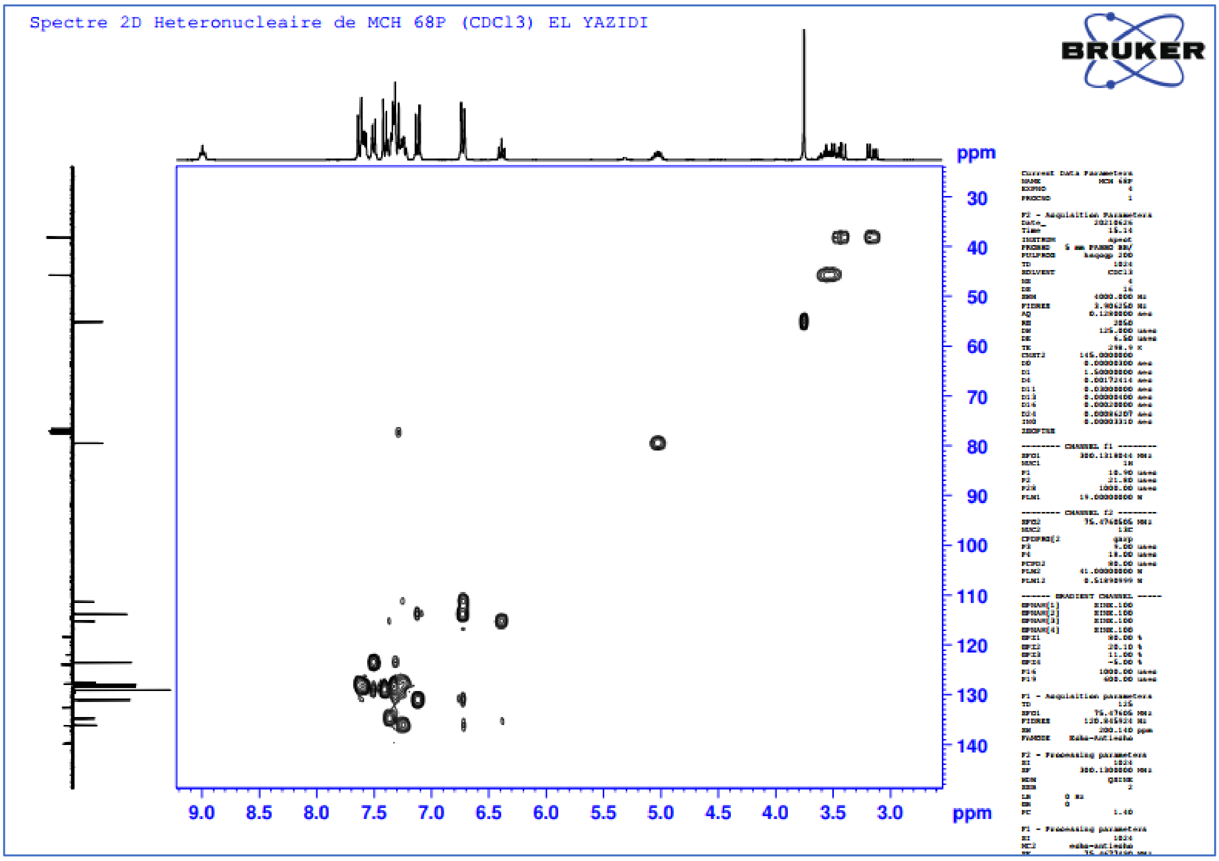

Figure S10. 2D-HSQC NMR spectrum of compound **6b**

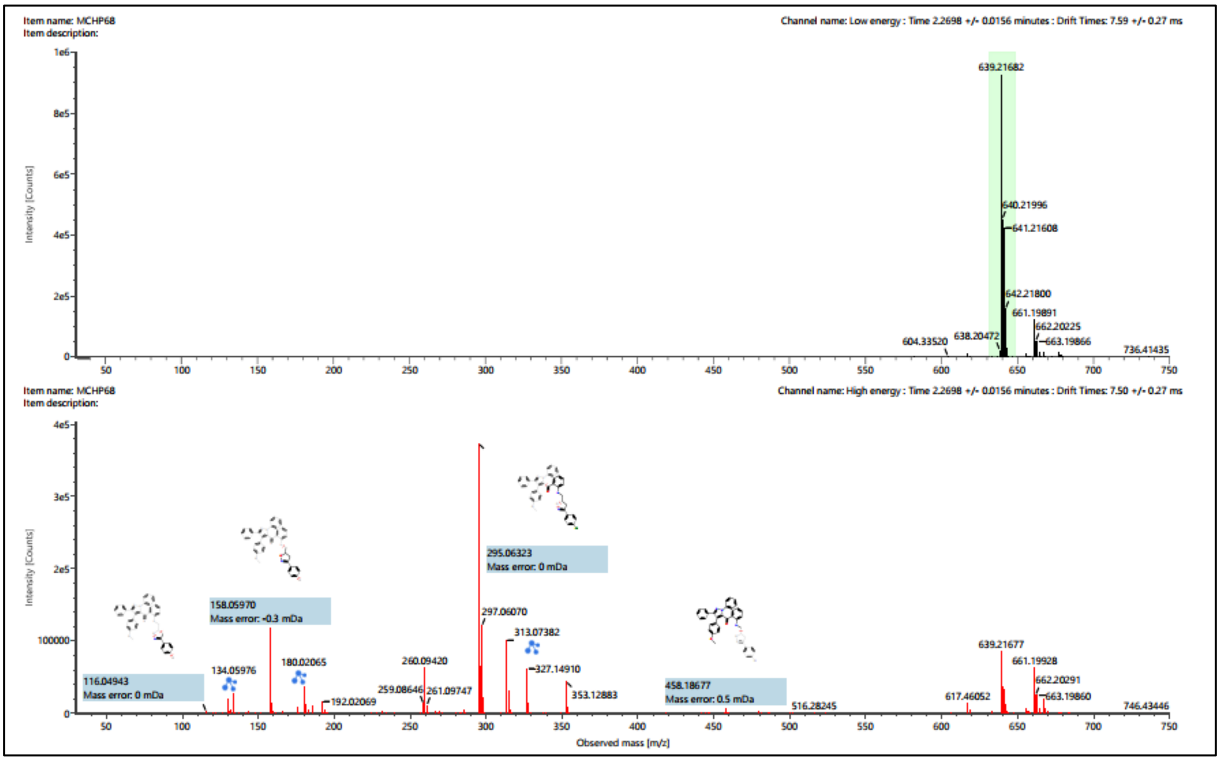

Figure S11. Mass spectrum of compound (**6b**)

c) 4-(4-bromophenyl)-5-(2-(3-(4-chlorophenyl)-4,5-dihydroisoxazol-5-yl)methylamino)benzoyl)-1,3-diphenyl-1H-pyrazole (6c)

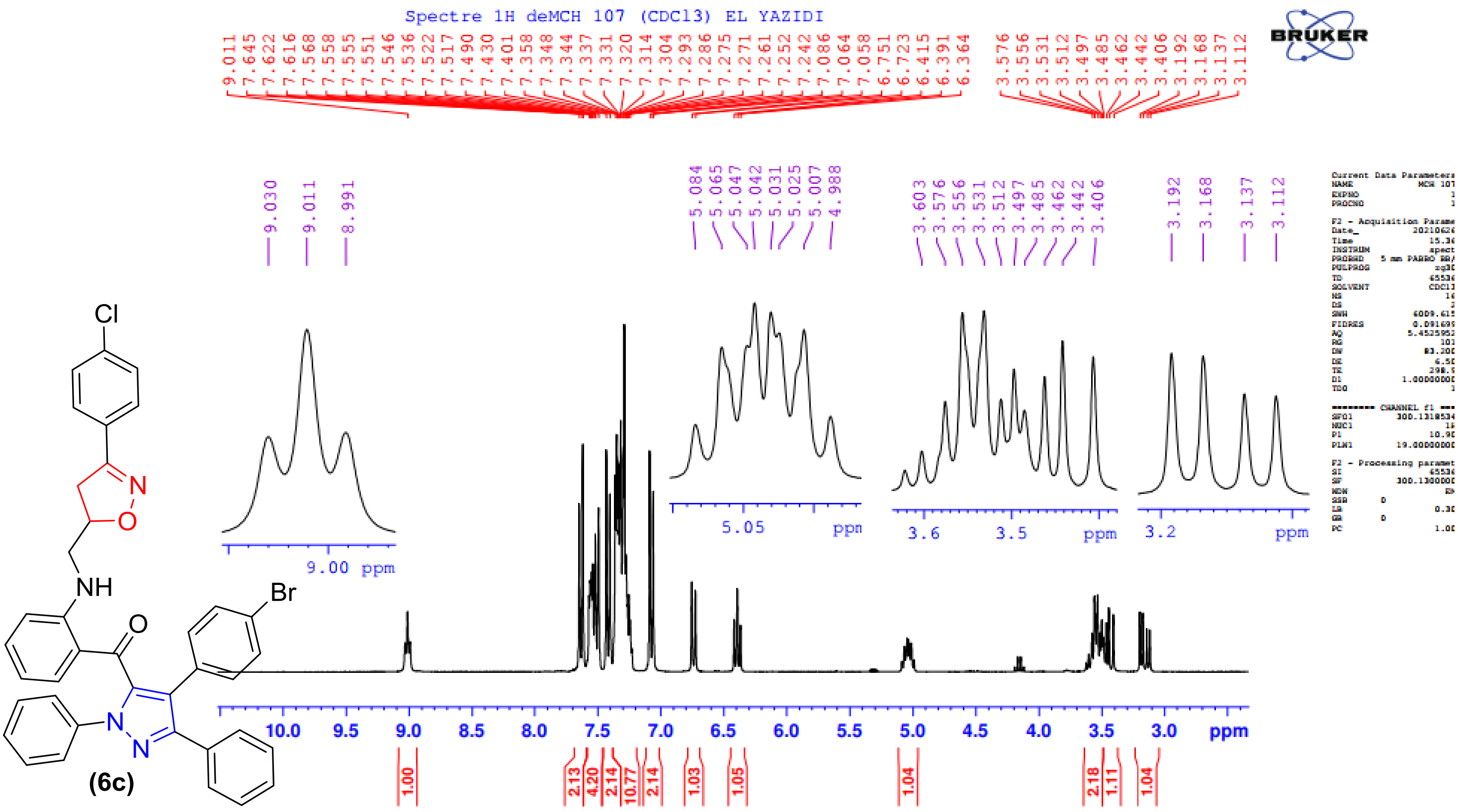

Figure S12. ^{1}H NMR spectrum (300 MHz, $CDCl_3$) of compound (**6c**)

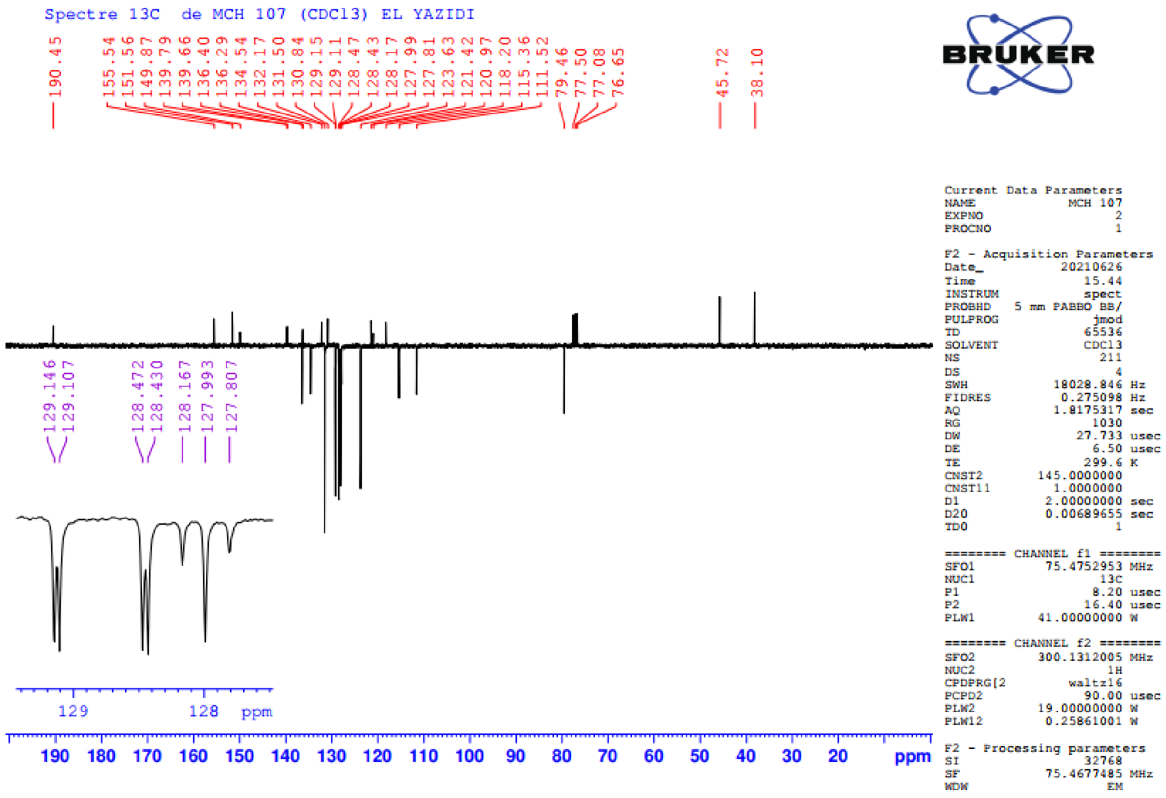

Figure S13. ^{13}C NMR spectrum (75 MHz, $CDCl_3$) of compound (**6c**)

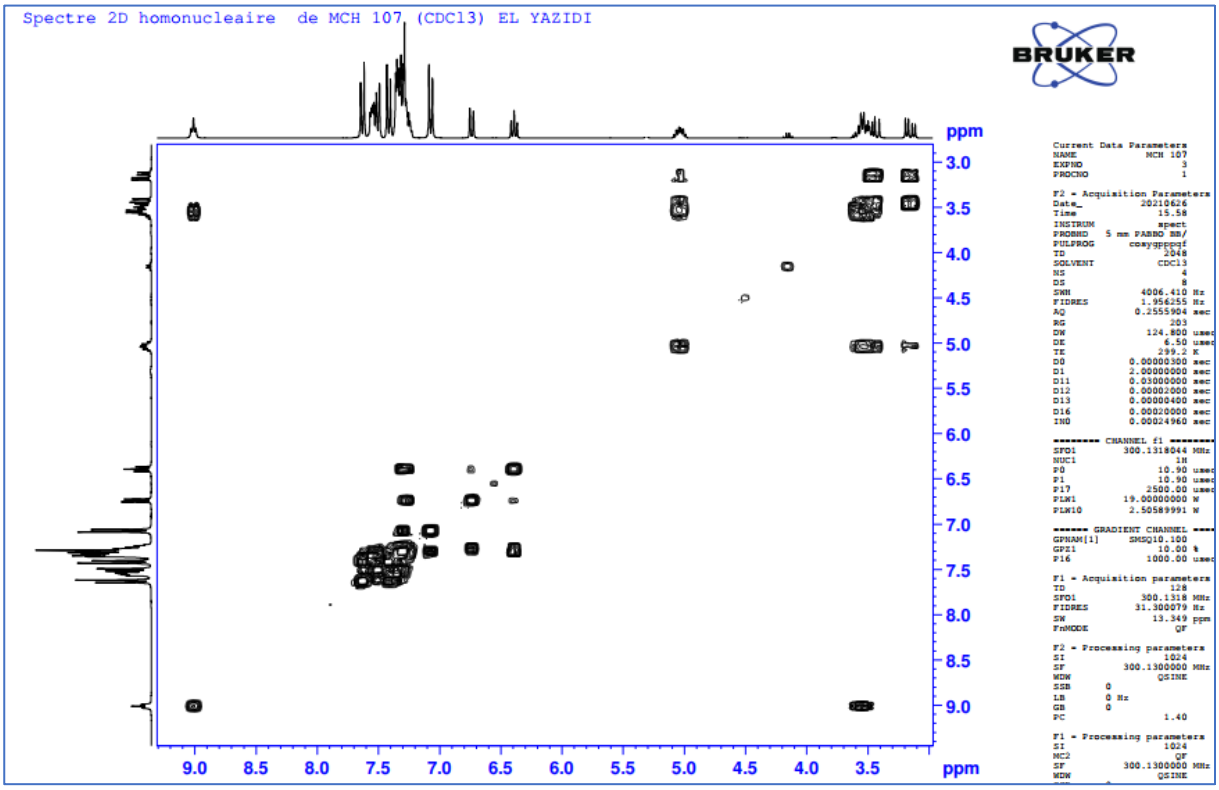

Figure S14. 2D-COSY NMR spectrum of compound **6c**

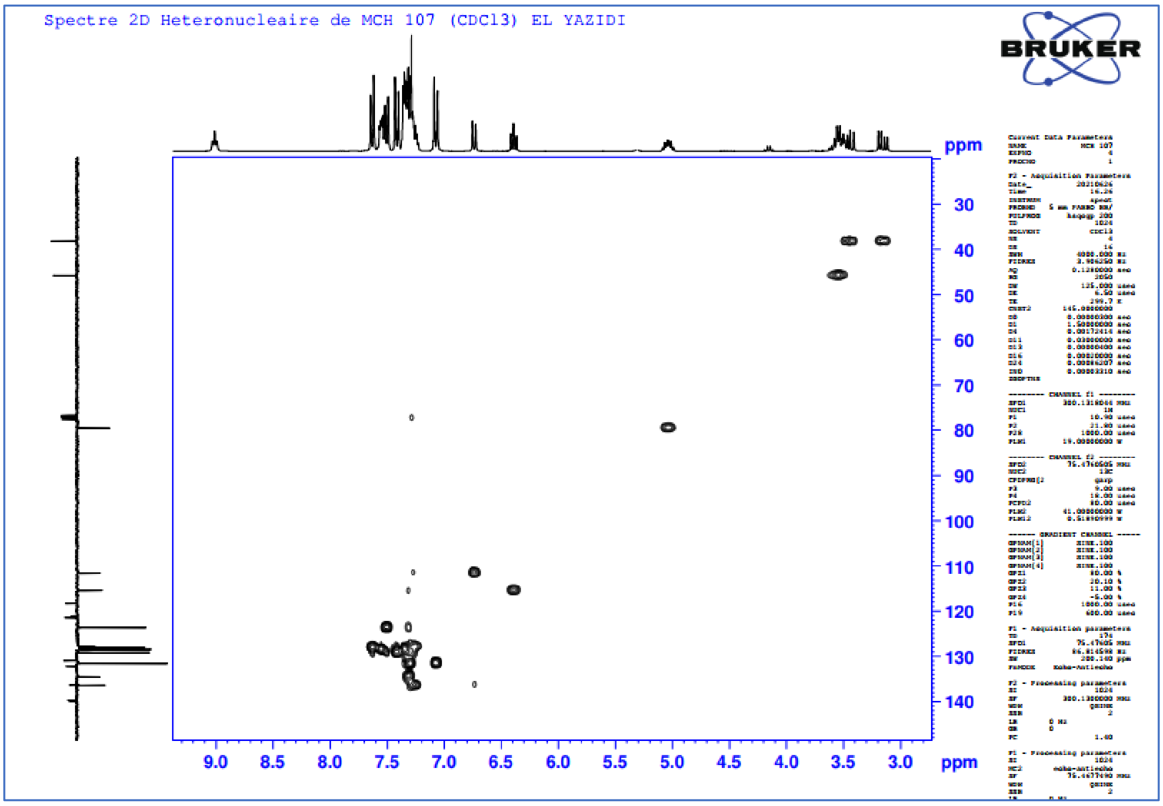

Figure S15. 2D-HSQC NMR spectrum of compound **6c**

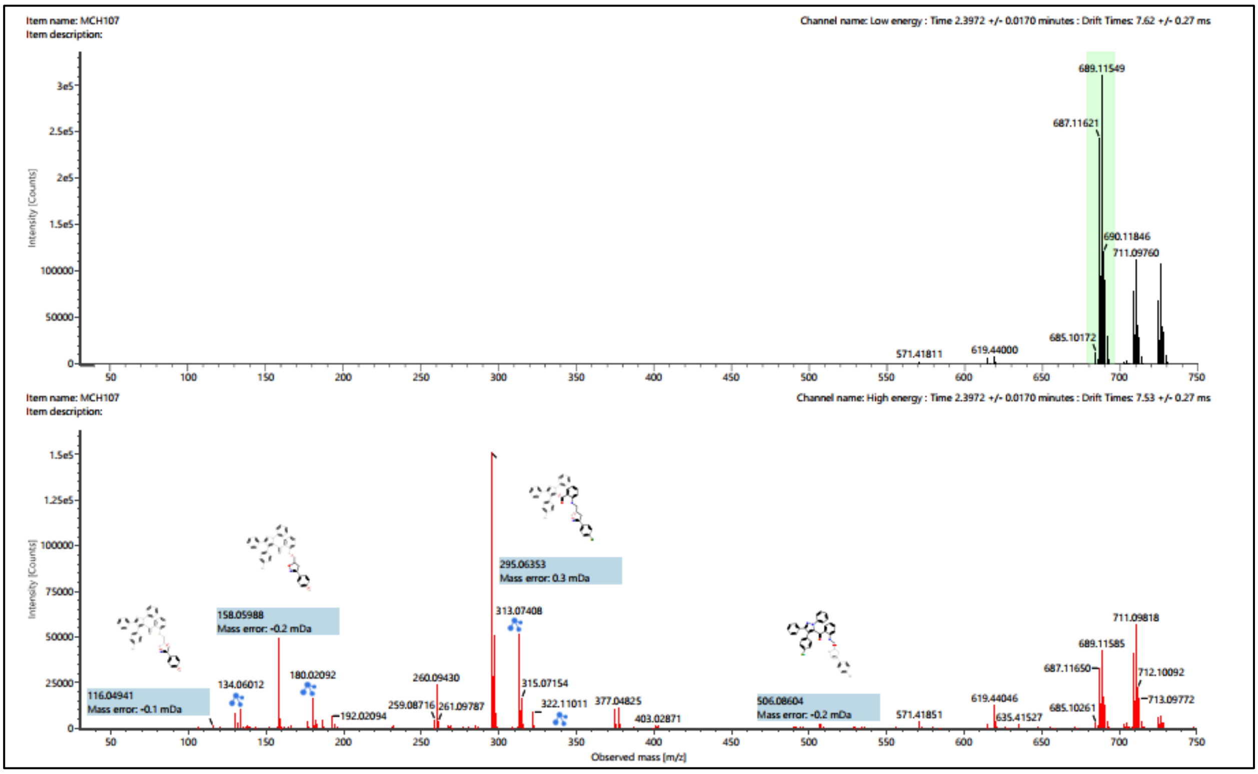

Figure S16. Mass spectrum of compound (**6c**)

d) 4-(4-chlorophenyl)-5-(2-(3-(4-chlorophenyl)-4,5-dihydroisoxazol-5-yl)methylamino)benzoyl)-1,3-diphenyl-1H-pyrazole (6d)

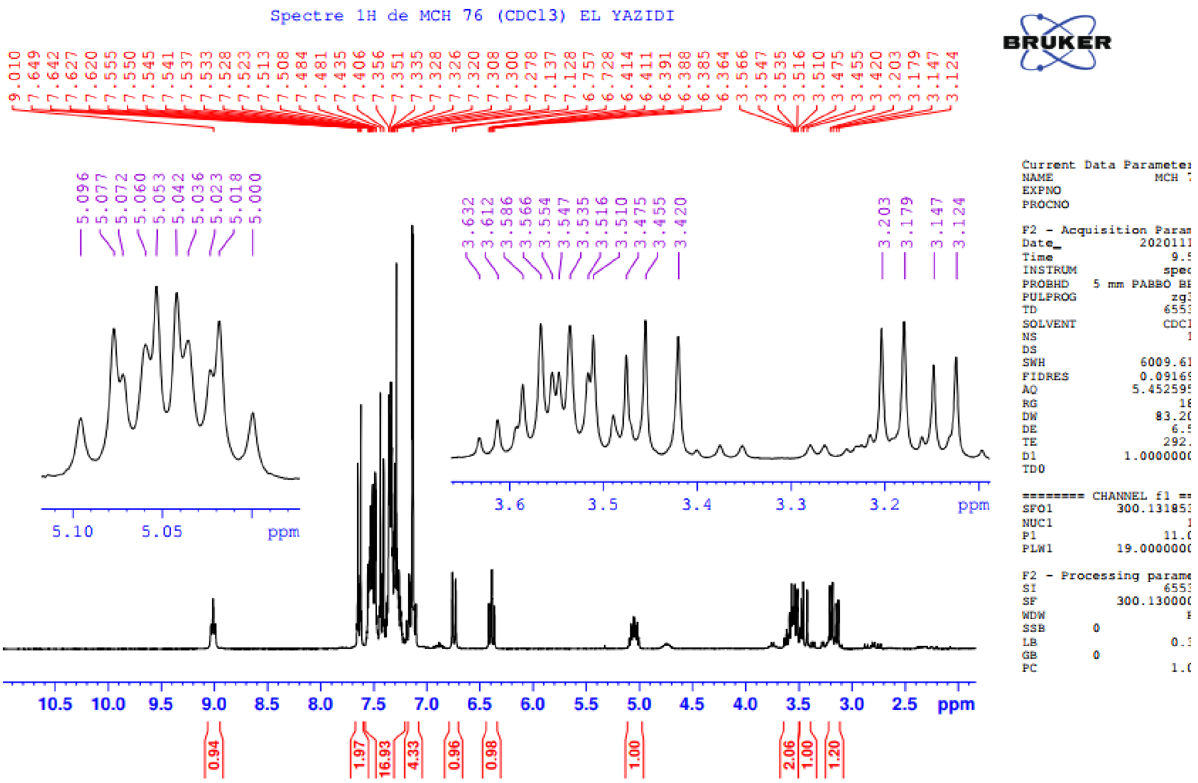

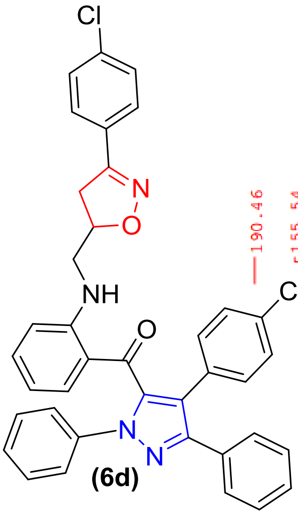

(6d)

Figure S17. ^{1}H NMR spectrum (300 MHz, $CDCl_3$) of compound (**6d**)

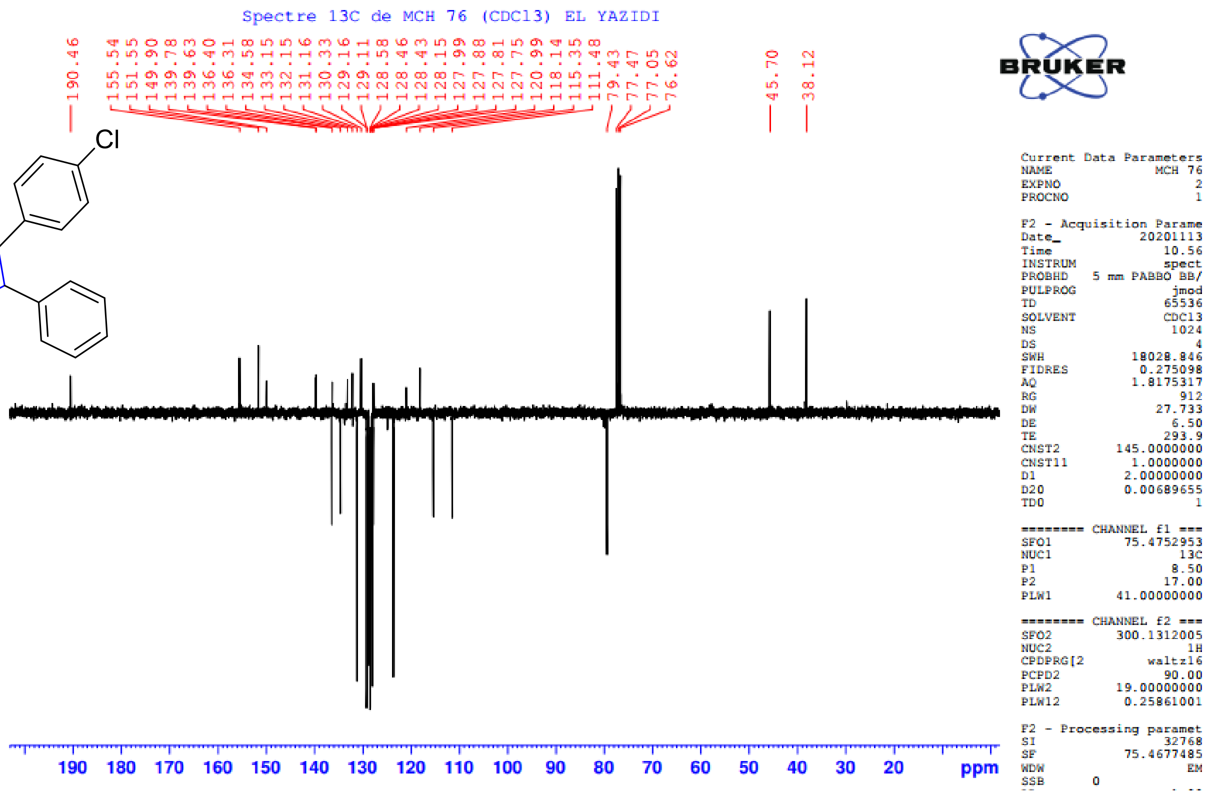

Figure S18. ^{13}C NMR spectrum (75 MHz, $CDCl_3$) of compound (**6d**)

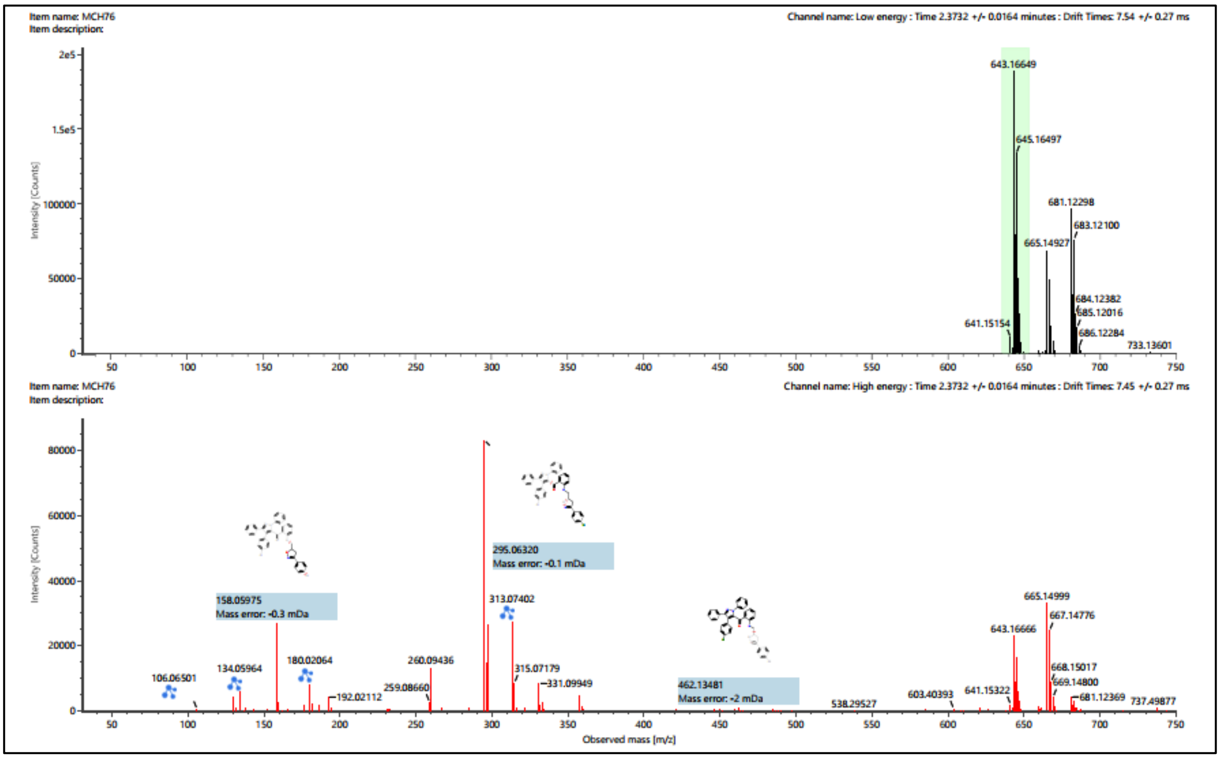

Figure S19. Mass spectrum of compound (**6d**)

e) 4-(4-bromophenyl)-5-(2-(3-(4-nitrophenyl)-4,5-dihydroisoxazol-5-yl)methylamino)benzoyl)-1,3-diphenyl-1H-pyrazole (6e)

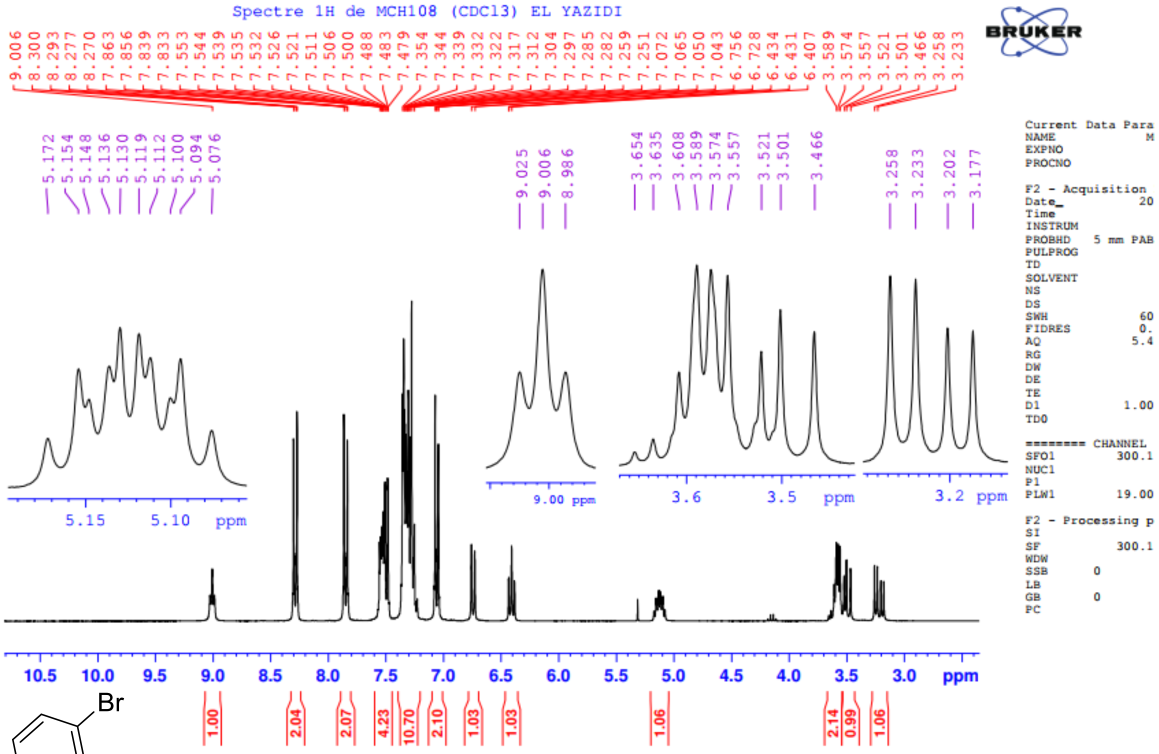

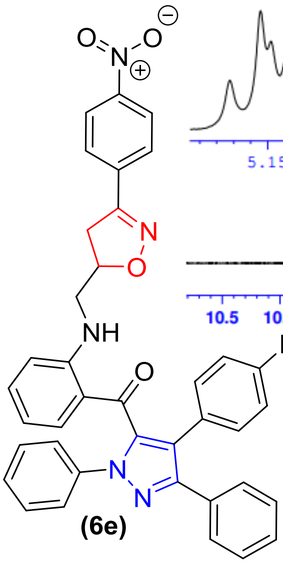

(6e)

Figure S20. ^{1}H NMR spectrum (300 MHz, $CDCl_3$) of compound (**6e**)

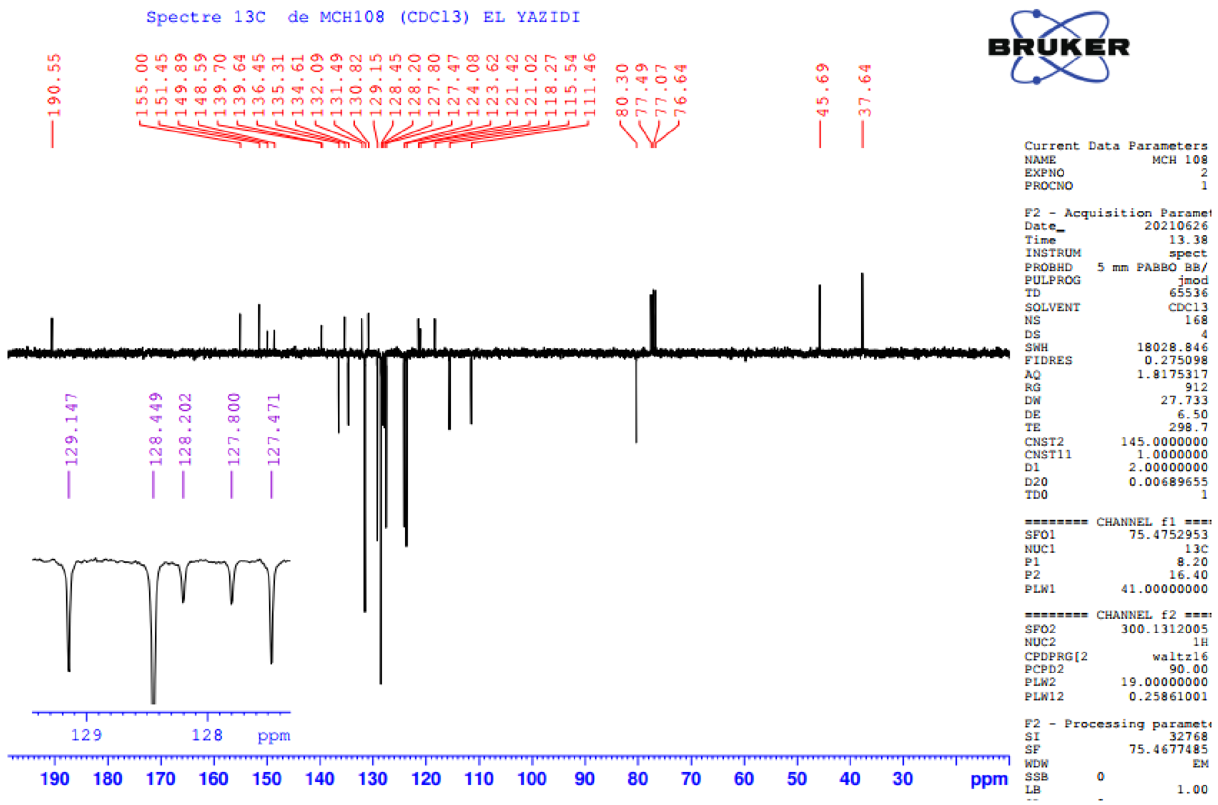

Figure S21. ^{13}C NMR spectrum (75 MHz, $CDCl_3$) of compound (**6e**)

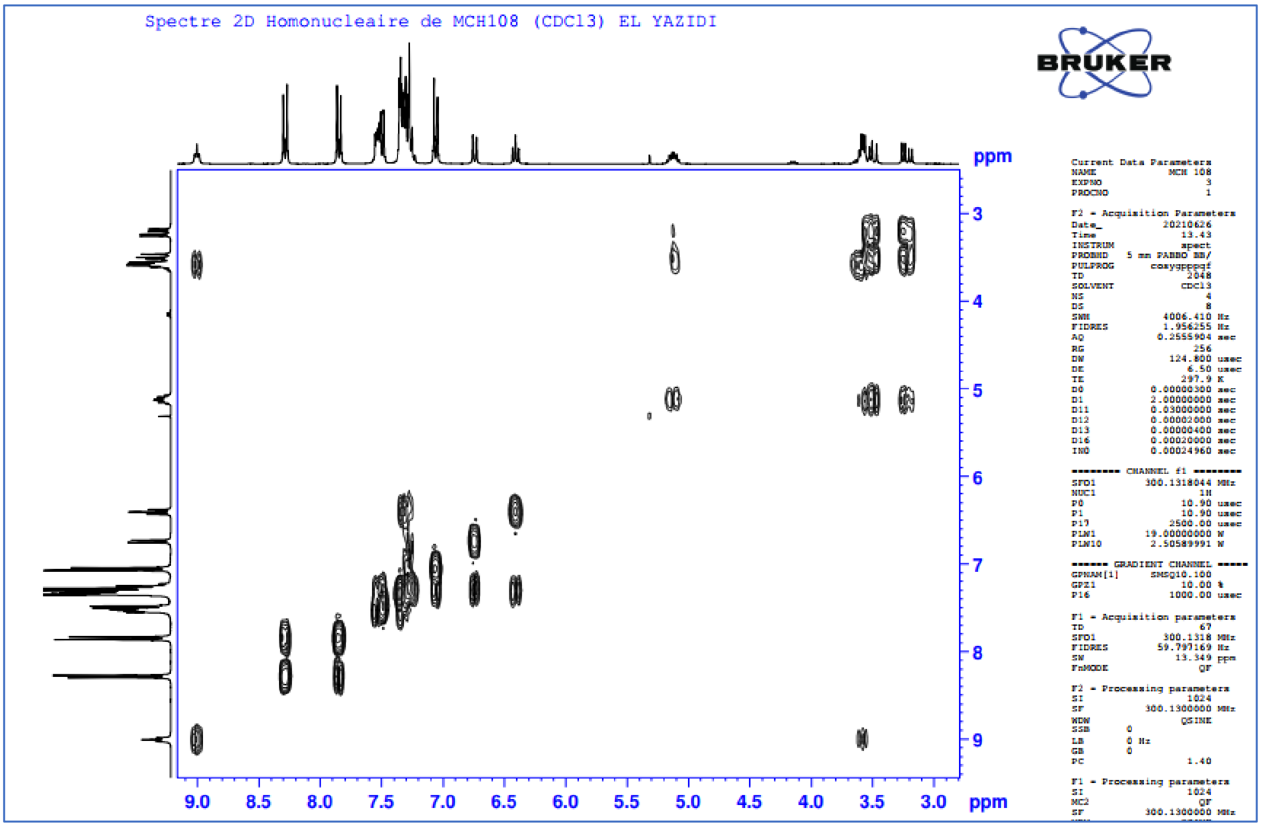

Figure S22. 2D-COSY NMR spectrum of compound (**6e**)

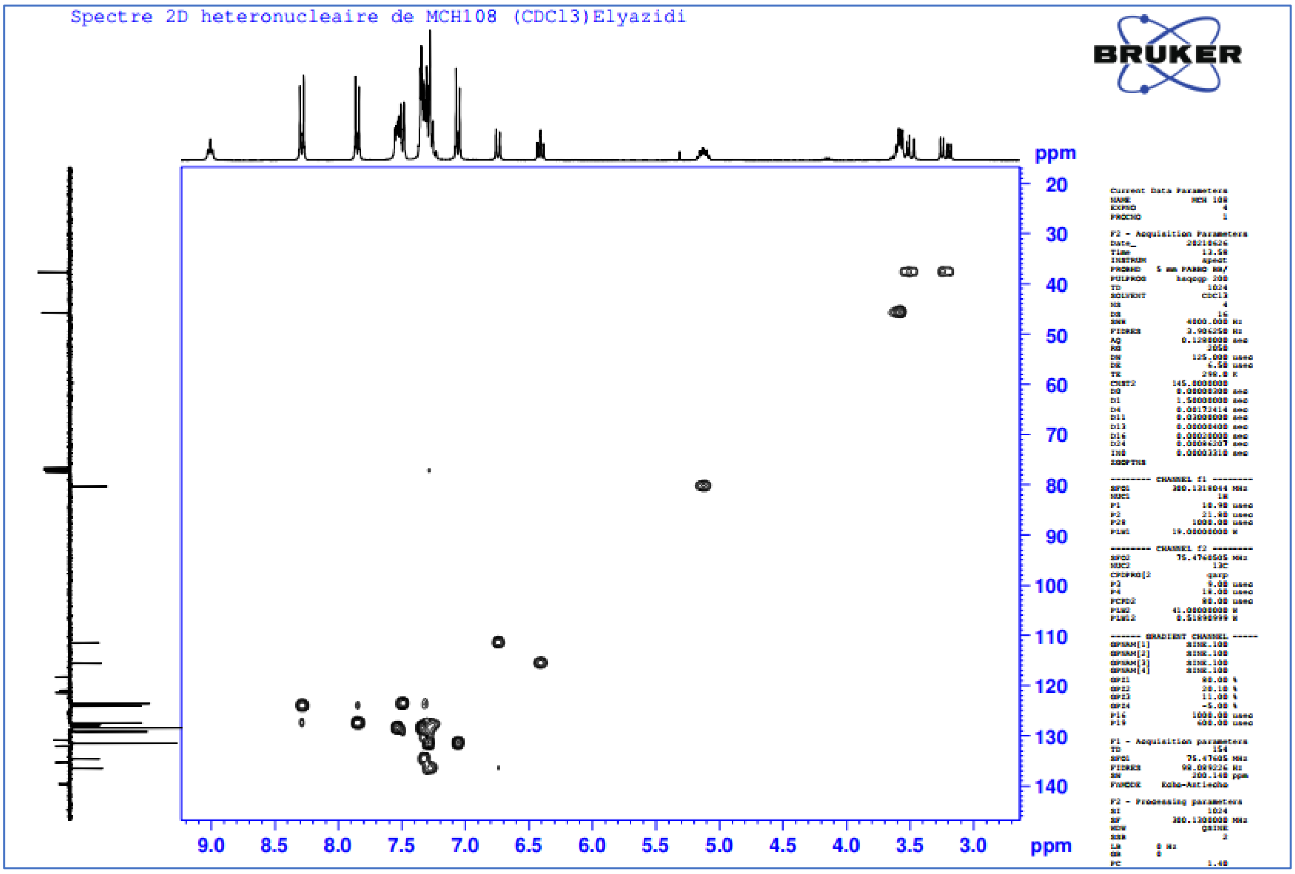

Figure S23. 2D-HSQC NMR spectrum of compound (**6e**)

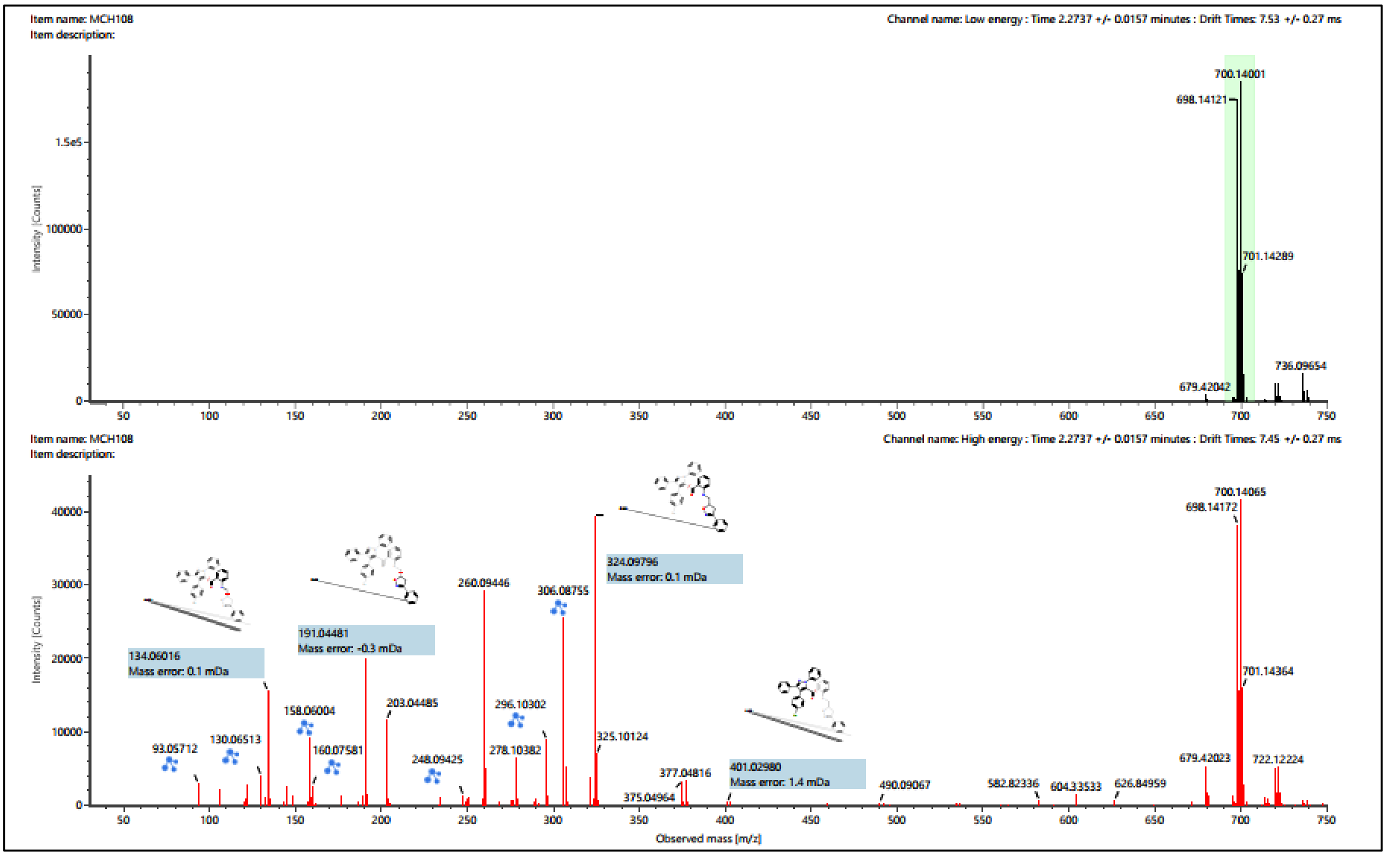

Figure S24. Mass spectrum of compound (**6e**)

f)* *5-(2-(3-(4-nitrophenyl)-4,5-dihydroisoxazol-5-yl)methylamino)benzoyl)-1,3-diphenyl--4-(p-tolyl)-1H-pyrazole (6f)

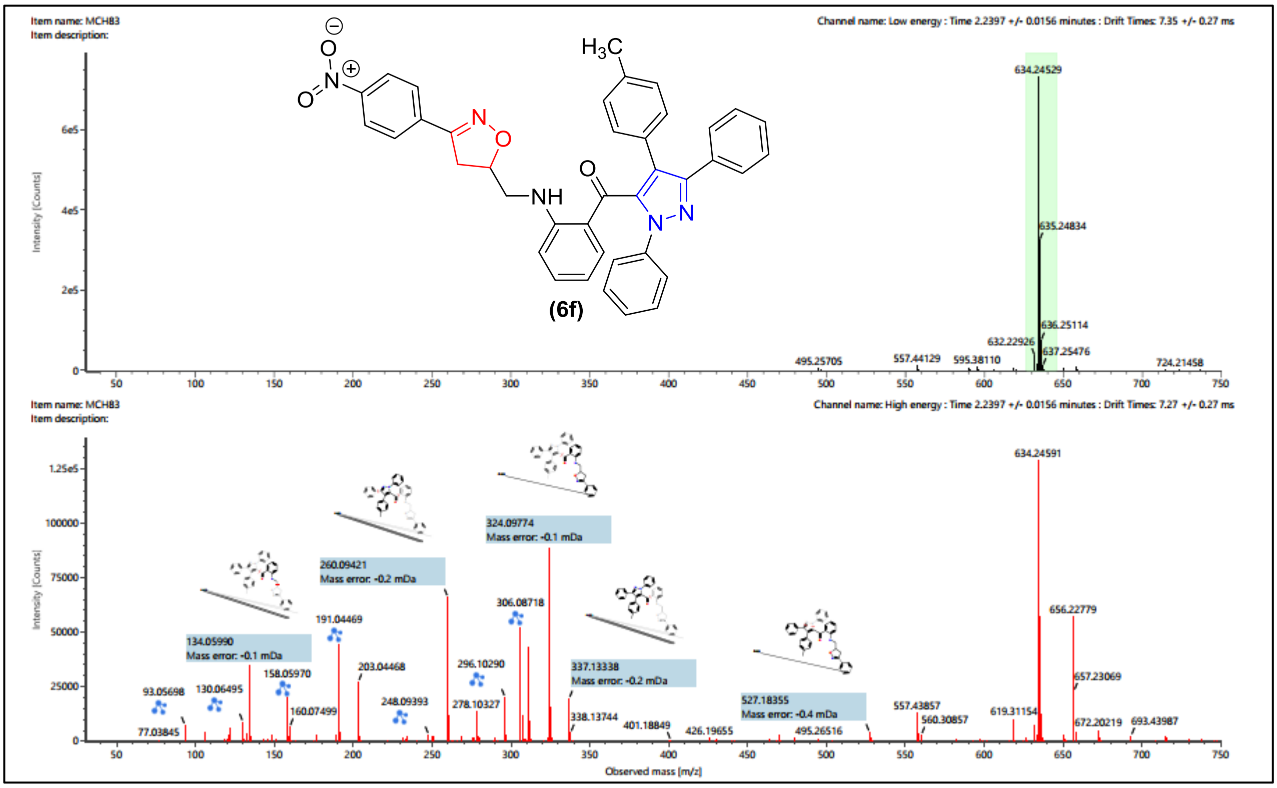

Figure S25. Mass spectrum of compound (**6f**)

g)* *4-(4-methoxyphenyl)-5-(2-(3-(4-nitrophenyl)-4,5-dihydroisoxazol-5-yl)methylamino)benzoyl)-1,3-diphenyl-1H-pyrazole (6g)

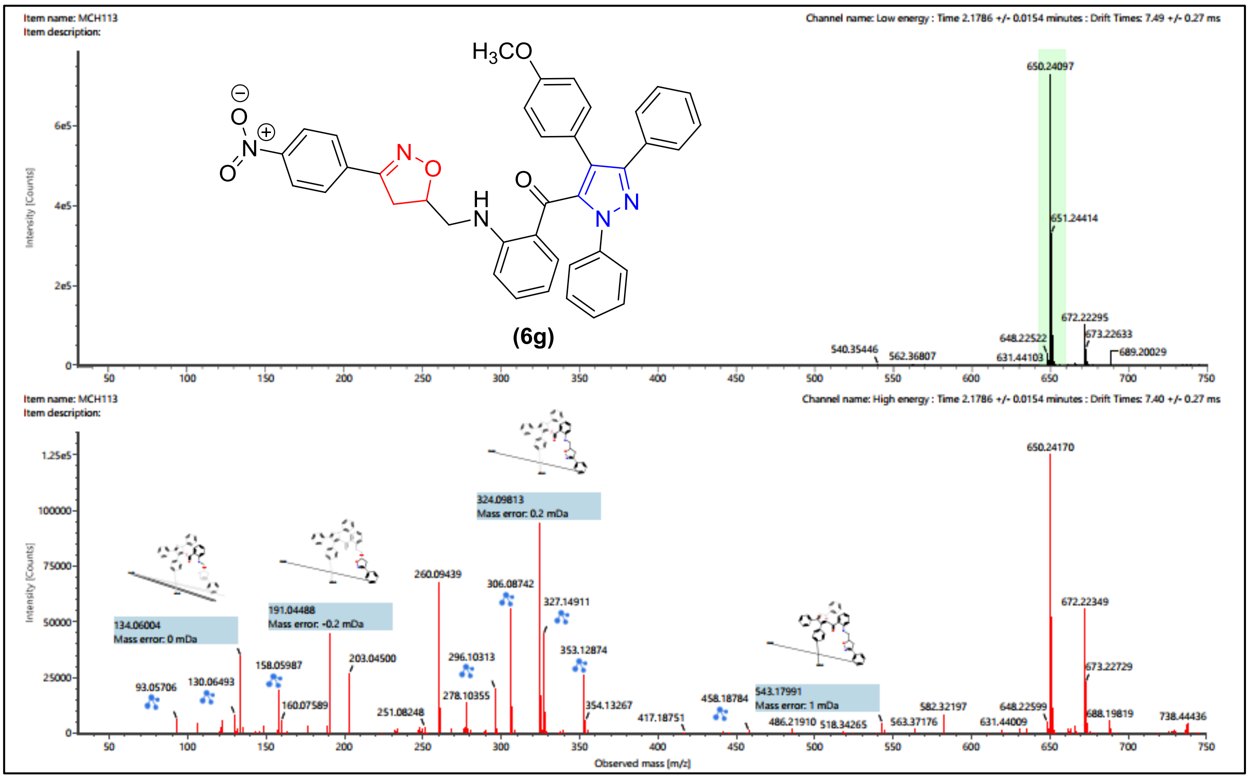

Figure S26. Mass spectrum of compound (**6g**)

h) 4-(4-methoxyphenyl)-5-(2-(3-(4-methoxyphenyl)-4,5-dihydroisoxazol-5-yl)methylamino)benzoyl)-1,3-diphenyl-1H-pyrazole (6i)

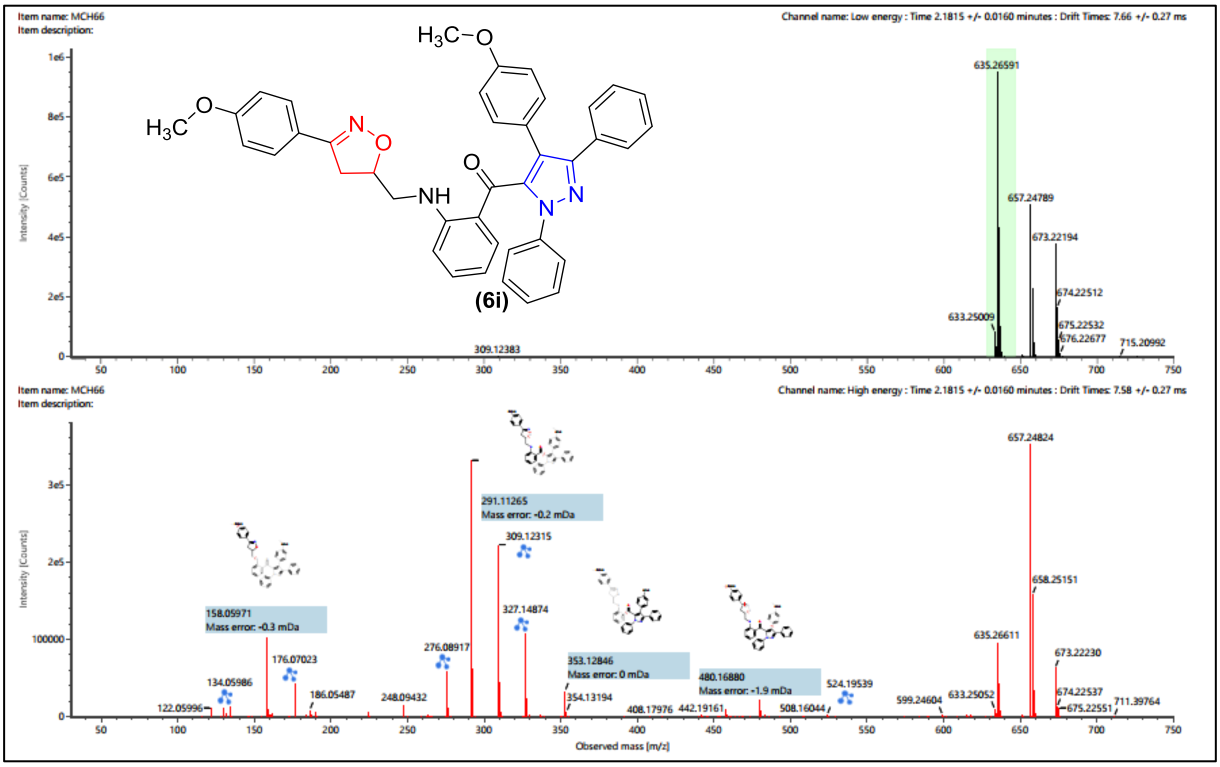

Figure S27. Mass spectrum of compound **(6i)**

i) 1,3-diphenyl-4-(p-tolyl)-5-(2-(3-(p-tolyl)-4,5-dihydroisoxazol-5-yl)methylamino)benzoyl)-1H-pyrazole (6j)

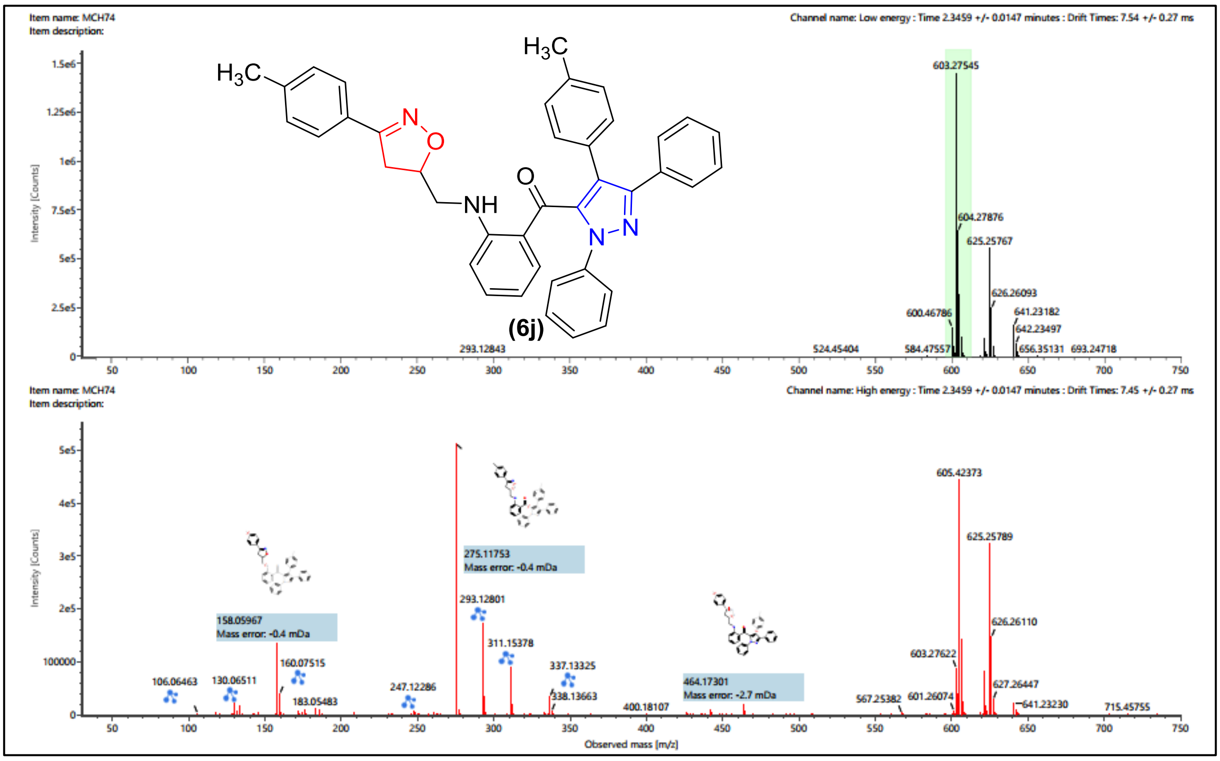

Figure S28. Mass spectrum of compound **(6j)**

j) 5-(2-(3-(4-bromophenyl)-4,5-dihydroisoxazol-5-yl)methylamino)benzoyl)-4-(4-methoxyphenyl)-1,3-diphenyl-1H-pyrazole (6k)

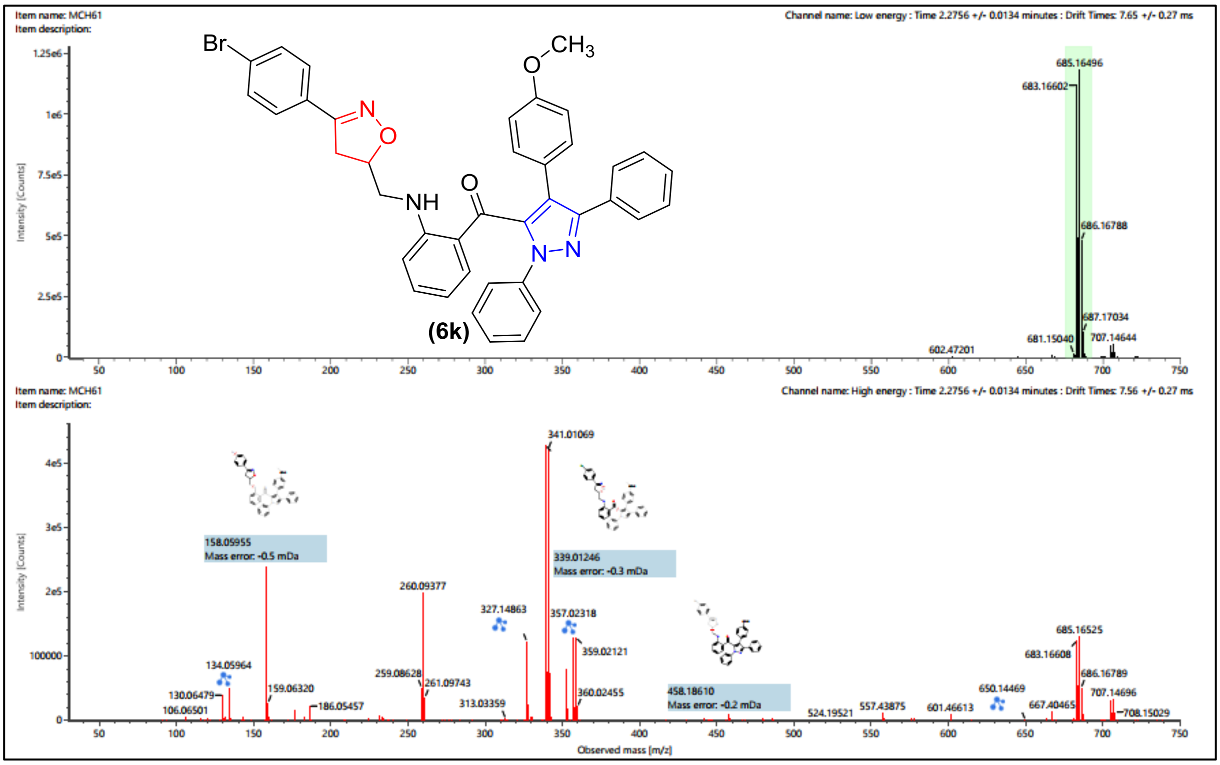

Figure S29. Mass spectrum of compound **(6k)**

k) 4-(4-methoxyphenyl)-1,3-diphenyl-5-(2-(3-(p-tolyl)-4,5-dihydroisoxazol-5-yl)methylamino)benzoyl)-1H-pyrazole (6l)

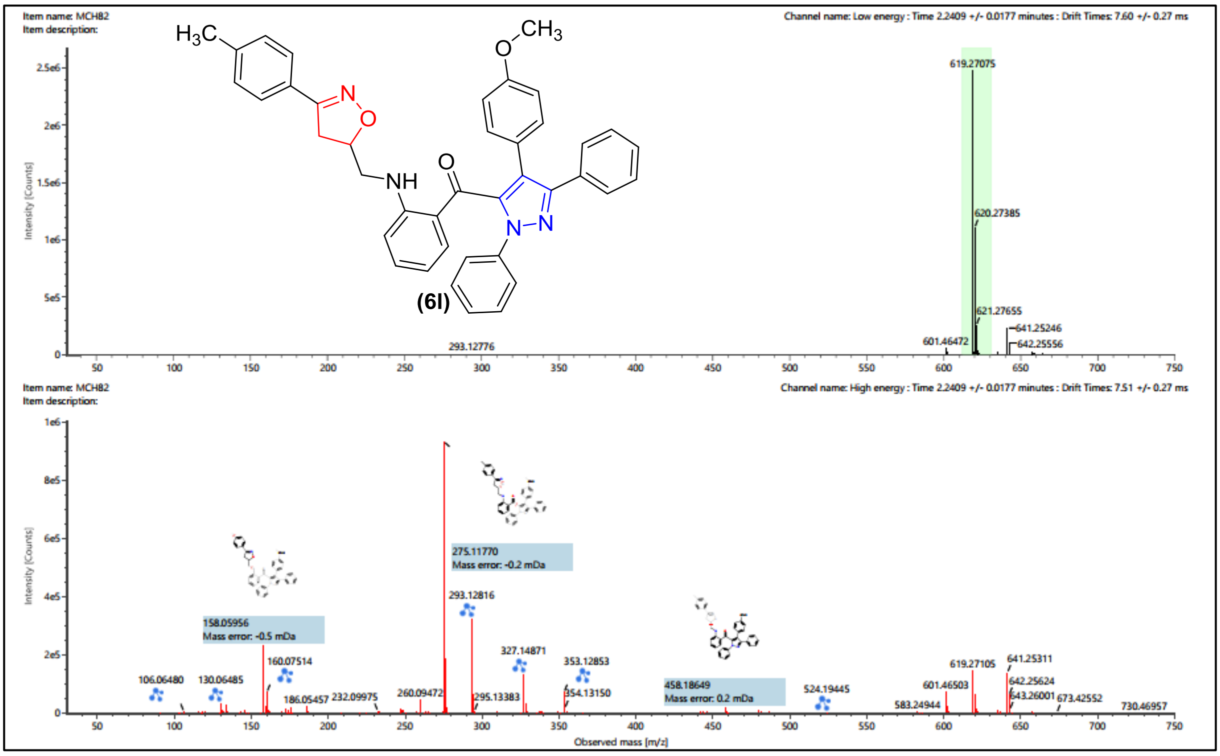

Figure S30. Mass spectrum of compound **(6l)**

l) 4-(4-chlorophenyl)-5-(2-(3-(2-chlorophenyl)-4,5-dihydroisoxazol-5-yl)methylamino)benzoyl)-1,3-diphenyl-1H-pyrazole (6m)

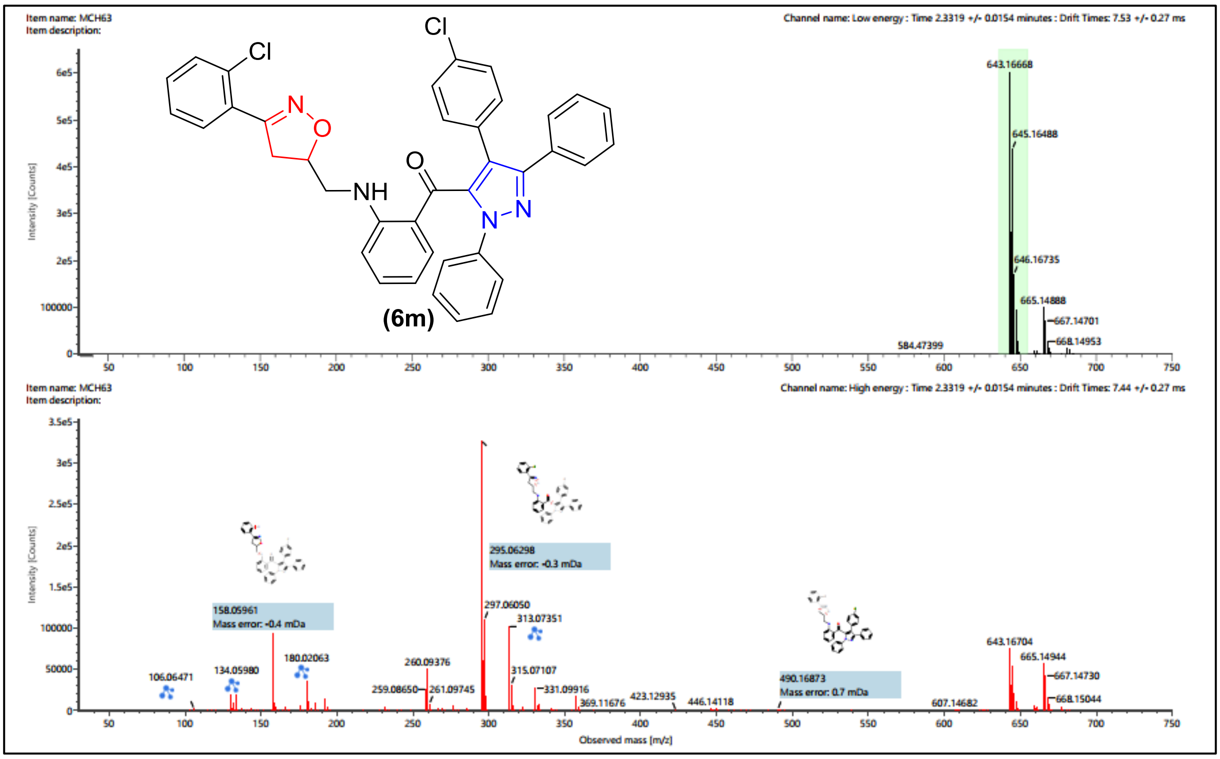

Figure S31. Mass spectrum of compound **(6m)**

S. Aureus

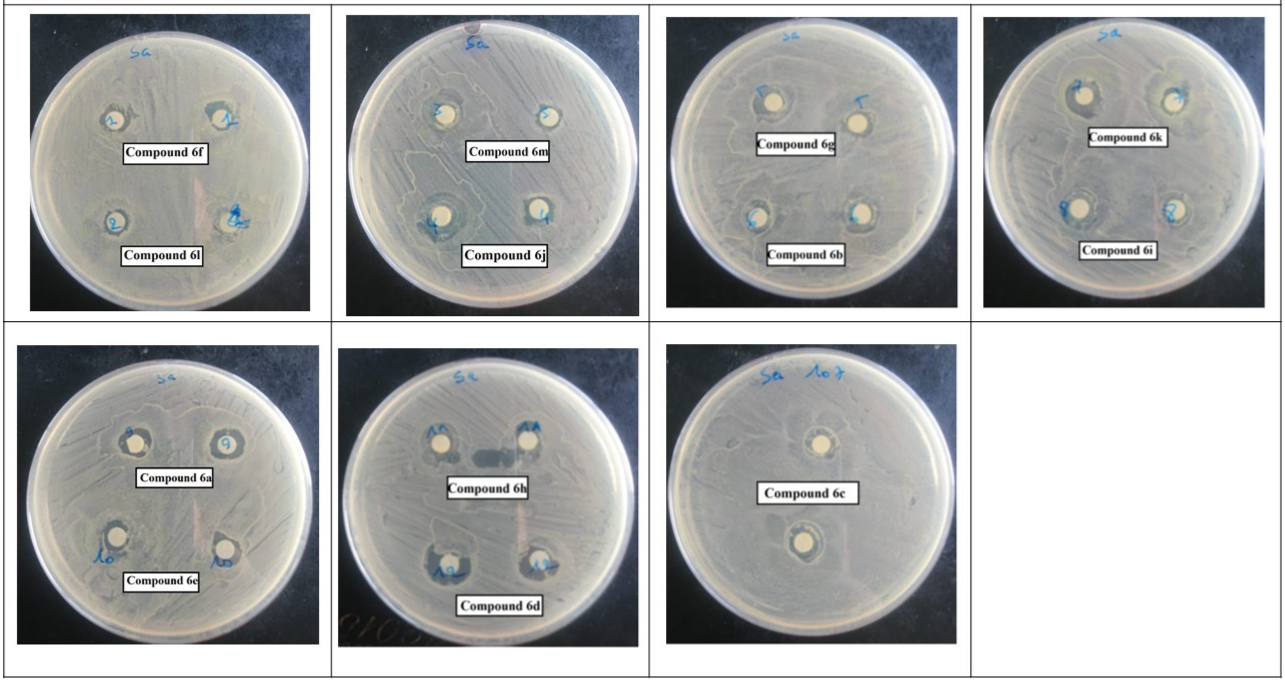

Figure S32. Photographs displaying the effect of the hybrid compounds on the *S. Aureus* strain

E. Coli

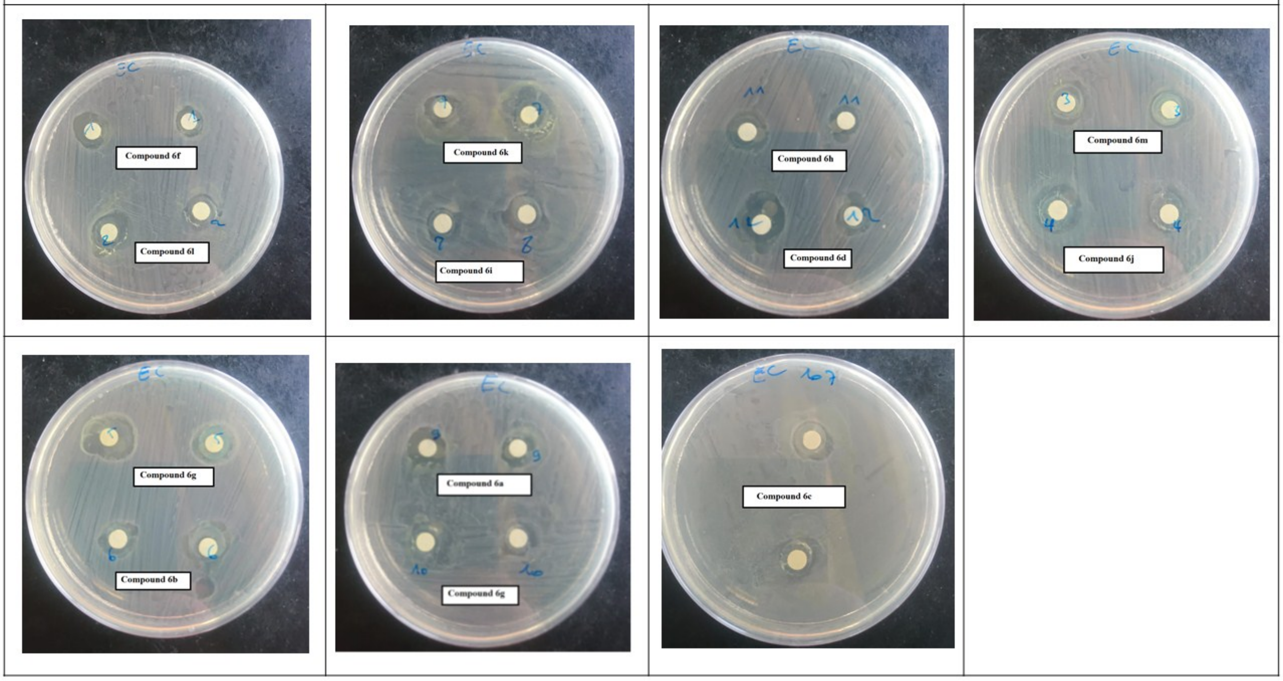

Figure S33. Photographs displaying the effect of the hybrid compounds on the *E. Coli* strain

References

(S1) Ribeiro, C. J. A.; Amaral, J. D.; Rodrigues, C. M. P.; Moreira, R.; Santos, M. M. M. Synthesis and Evaluation of Spiroisoxazoline Oxindoles as Anticancer Agents. *Bioorganic Med. Chem.* **2014**, *22* (1), 577–584. https://doi.org/10.1016/j.bmc.2013.10.048.

(S2) Liu, K.-C.; Shelton, B. R.; Howe, R. K. A Particularly Convenient Preparation of Benzohydroximinoyl Chlorides (Nitrile Oxide Precursors). *J. Org. Chem.* **1980**, *45* (19), 3916–3918. https://doi.org/10.1021/jo01307a039.

(S3) Groundwater, P. W.; Nyerges, M.; Fejes, I.; Hibbs, D. E.; Bendell, D.; Anderson, R. J.; McKillop, A.; Sharif, T.; Zhang, W. Preparation and Reactivity of Some Stable Nitrile Oxides and Nitrones. *Arkivoc* **2000**, *2000* (5), 684–697. https://doi.org/10.3998/ark.5550190.0001.503.

(S4) Chalkha, M.; Akhazzane, M.; Moussaid, F. Z.; Daoui, O.; Nakkabi, A.; Bakhouch, M.; Chtita, S.; Elkhattabi, S.; Iraqi Housseini, A.; El Yazidi, M. Design, Synthesis, Characterization, in Vitro Screening, Molecular Docking, 3D-QSAR, and ADME-Tox Investigations of Novel Pyrazole Derivatives as Antimicrobial Agents. *New J. Chem.* **2022**, *46* (6), 2747–2760. https://doi.org/10.1039/d1nj05621b.

(S5) Ravindar, L.; Bukhari, S. N. A.; Rakesh, K. P.; Manukumar, H. M.; Vivek, H. K.; Mallesha, N.; Xie, Z. Z.; Qin, H. L. Aryl Fluorosulfate Analogues as Potent Antimicrobial Agents: SAR, Cytotoxicity and Docking Studies. *Bioorg. Chem.* **2018**, *81*, 107–118. https://doi.org/10.1016/j.bioorg.2018.08.001.

(S6) Esfahani, S. N.; Damavandi, M. S.; Sadeghi, P.; Nazifi, Z.; Salari-Jazi, A.; Massah, A. R. Synthesis of Some Novel Coumarin Isoxazol Sulfonamide Hybrid Compounds, 3D-QSAR Studies, and Antibacterial Evaluation. *Sci. Rep.* **2021**, *11* (1), 1–15. https://doi.org/10.1038/s41598-021-99618-w.

(S7) Barmak, A.; Niknam, K.; Mohebbi, G.; Pournabi, H. Antibacterial Studies of Hydroxyspiro[Indoline-3,9-Xanthene]Trione against Spiro[Indoline3,9-Xanthene]Trione and Their Use as Acetyl and Butyrylcholinesterase Inhibitors. *Microb. Pathog.* **2019**, *130* (March), 95–99. https://doi.org/10.1016/j.micpath.2019.03.002.

(S8) Chebbac, K.; Ghneim, H. K.; El Moussaoui, A.; Bourhia, M.; El Barnossi, A.; Ouaritini, Z. B.; Salamatullah, A. M.; Alzahrani, A.; Aboul-Soud, M. A. M.; Giesy, J. P.; Guemmouh, R. Antioxidant and Antimicrobial Activities of Chemically-Characterized Essential Oil from Artemisia Aragonensis Lam. against Drug-Resistant Microbes. *Molecules* **2022**, *27* (3), 1136. https://doi.org/10.3390/molecules27031136.

(S9) Abu-Dief, A. M.; Abdel-Rahman, L. H.; Abdel-Mawgoud, A. A. H. A Robust in Vitro Anticancer, Antioxidant and Antimicrobial Agents Based on New Metal-Azomethine Chelates Incorporating Ag(I), Pd (II) and VO (II) Cations: Probing the Aspects of DNA Interaction. *Appl. Organomet. Chem.* **2020**, *34* (2), 1–20. https://doi.org/10.1002/aoc.5373.

(S10) Shehab, W. S.; Aziz, M. A.; Elhoseni, N. K. R.; Assy, M. G.; Abdellattif, M. H.; Hamed, E. O. Design, Synthesis, Molecular Docking, and Evaluation Antioxidant and Antimicrobial Activities for Novel 3-Phenylimidazolidin-4-One and 2-Aminothiazol-4-One Derivatives. *Molecules* **2022**, *27* (3), 767. https://doi.org/10.3390/molecules27030767.

(S11) Chalkha, M.; Moussaoui, A. El; Hadda, T. Ben; Berredjem, M.; Bouzina, A.; Almalki, F. A.; Saghrouchni, H.; Bakhouch, M.; Saadi, M.; Ammari, L. El; Abdellatiif, M. H.; Yazidi, M. El. Crystallographic Study, Biological Evaluation and DFT/POM/Docking Analyses of Pyrazole Linked Amide Conjugates: Identification of Antimicrobial and Antitumor Pharmacophore Sites. *J. Mol. Struct.* **2022**, *1252*, 131818. https://doi.org/https://doi.org/10.1016/j.molstruc.2021.131818.

(S12) Clinical and Laboratory Standards Institute (CLSI). Reference Method for Broth Dilution Antifungal Susceptibility Testing of Yeasts; Approved Standard Third Edition. CLSI Document M27-A3. *Wayne, PA: Clinical and Laboratory Standards Institue*; **2008**.

(S13) Clinical and Laboratory Standards Institute (CLSI). Methods for Dilution Antimicrobial Susceptibility Tests for Bacteria That Grow Aerobically; Approved Standard-Ninth Edition. CLSI Document M07-A9. *Wayne, PA: Clinical and Laboratory Standards Institute*; **2012**.
